# Feasibility of individualized home exercise programs for patients with head and neck cancer–study protocol and first results of a multicentre single-arm intervention trial (OSHO #94)

**DOI:** 10.1371/journal.pone.0301304

**Published:** 2024-08-22

**Authors:** Sabine Felser, Julia Rogahn, Änne Glass, Lars Arne Bonke, Daniel Fabian Strüder, Jana Stolle, Susann Schulze, Markus Blaurock, Ursula Kriesen, Christian Junghanss, Christina Grosse-Thie

**Affiliations:** 1 Department of Internal Medicine, Clinic III–Hematology, Oncology and Palliative Care, Rostock University Medical Center, Rostock, Germany; 2 Institute of Biostatistics and Informatics in Medicine, Rostock University Medical Center, Rostock, Germany; 3 Department of Otorhinolaryngology, Head and Neck Surgery "Otto Koerner", Rostock University Medical Center, Rostock, Germany; 4 Krukenberg Cancer Center Halle, University Hospital Halle, Halle (Saale), Germany; 5 Department of Internal Medicine, Medical Clinic II, Carl-von-Basedow-Klinikum, Merseburg, Germany; 6 Department of Otorhinolaryngology, Head and Neck Surgery, University Medicine Greifswald, Greifswald, Germany; 7 Hematology and Oncology Practice, Rostock, Germany; Fred Hutchinson Cancer Research Center, UNITED STATES OF AMERICA

## Abstract

**Introduction:**

Patients with head and neck cancer (PwHNC) benefit from targeted exercise interventions: symptom relief, compensation for dysfunction, improvement in quality of life (QoL). Data on acceptance physical interventions in PwHNC are rare. The ‘OSHO #94’ trial investigates the short- and medium-term effects of individualized home exercise in PwHNC on QoL, physical activity and functionality. The study includes a feasibility phase (proof of concept) in order to evaluate the acceptance. Here we present the study protocol as well as the feasibility results.

**Methods and analysis:**

This prospective, multicentre, single-arm intervention study includes PwHNC ≥18 years of age in aftercare or palliative care with stable remission under immunotherapy. The study opened in January 01, 2021, with estimated completion by December 31, 2024. The PwHNC receive an individualized home exercise program consisting of mobilization, coordination, strengthening and stretching exercises. This should be carried out at least three times a week over 12 weeks for 15 to 30 minutes, supplemented by aerobic training two to three times a week for 30 minutes (intervention). Once weekly telephone calls with a physiotherapist are performed. Subsequently, there is a 12-week follow-up (FU) without exercise specifications/contact. Outcomes are measured before and after the intervention and following the FU. Primary outcome of the feasibility phase (n = 25) was the determination of the dropout rate during the intervention with a termination cut off if more than 30% PwHNC withdrew premature. The primary outcome of the OSHO #94’ trial (N = 53) is the change in global QoL score from pre- to post-intervention (EORTC QLQ-C30). Secondary outcomes include clinical and patient-reported measures, training details as well as functional diagnostic data (e.g. level of physical activity, training frequency, flexibility, fall risk and aerobic performance).

**Results:**

25 PwHNC were enrolled onto the feasibility cohort. Only16% (4/25 patients) did not complete the study. Therefore, recruitment of PwHNC was continued. The dropout rate was adjusted from 30% (N = 60) to 20% (N = 53, calculated sample size n = 42 PwHNC and 20% (n = 11) to dropout).

**Conclusions:**

Individualized home exercise programs in PwHNC in aftercare seem feasible. Consequently, the aim is now to evaluate the short and medium-term effects of individualized home exercise.

## Introduction

Head and neck cancer (HNC) comprises various types of cancer that occur in the head and neck area, including malignant neoplasms of the oral cavity, pharynx, larynx, nose and paranasal sinuses. Many patients with HNC (PwHNC) have clinically relevant functional deficits, often in the areas of food-intake, breathing, speech, pain, mood and neck and shoulder mobility, due to the location of the tumours and intensive local therapy procedures [1–4], e.g. operation and/or radiotherapy. In addition, there may be visible disfigurements in the face and neck area, weight loss and sarcopenia [5], body image disturbance [6] or other symptoms such as fatigue. These acute and sometimes chronic disease- and therapy-related functional deficits and side effects often impair the health-related quality of life (QoL) of those affected [5–9]. Recent advances in the diagnosis and treatment of PwHNC have significantly improved the survival of PwHNC [10]. As a result, there are more long-term survivors [11], and, especially under new approaches such as immunotherapy (ICT-immune-checkpoint therapy), a small group of PwHNC can reach stable remissions even in a primary incurable situation [12]. Therefore, other dimensions of the treatment outcome, such as physical status and functional abilities, psychological status and wellbeing, are becoming increasingly important [13]. Consequently, the treatment of functional deficits and the improvement of QoL is an essential task in the context of interdisciplinary rehabilitation of PwHNC [1, 2].

The most commonly used supportive interventions in HNC survivors to date have focused on monitoring/treatment of physical effects [14]. Physical exercise is a feasible, safe and promising approach to improve QoL in HNC survivors. In particular, 12-week training programs with aerobic activity (walking) or progressive resistance training for the whole body showed great benefit for improving QoL perception in HNC survivors [15]. In addition, reviews and meta-analyses indicate that PwHNC benefit from exercise interventions during and after medical therapy in terms of physical functionality (muscle strength, cardiorespiratory fitness, flexibility), (shoulder) pain reduction and fatigue relief. The effects described with regard to body composition are heterogeneous [16–19]. Consequently, the American Head and Neck Society’s 2022 statement on exercise therapy calls for, among other things, 1. early screening of PwHNC for rehabilitation needs using, 2. objective assessments, 3. referral of PwHNC to qualified therapists, and 4. motivating and encouraging PwHNC to engage in regular physical activity [20]. The fact that particular attention should be paid to the latter point is illustrated by study results from Taiwan and Sweden, which show that PwHNC are insufficiently physically active [21, 22]. According to the results of Fang et al. [21], only 17% of 108 HNC survivors met the WHO criteria for physical activity. Those reported less fatigue and better QoL compared to PwHNC who did not meet the criteria. Regardless, the PwHNC had poorer overall physical fitness compared to results from normative data of subjects from Taiwanese work fitness measurements. Reviews of the barriers revealed that in addition to physical problems, time pressure, lack of motivation, and lack of knowledge are the main reasons for insufficient physical activity in PwHNC [23, 24]. The results also revealed that the exercise preferences of the PwHNC differ in terms of type, location, company, intensity, frequency and supervision. It is known from surveys in England, the US Midwest and Canada that PwHNC prefer (un)supervised exercise programs, alone or with family members, at moderate intensity, at home or outdoors at different times [25–27]. To summarize the current findings, it can be concluded that individual exercise programs that can be carried out flexibly at home and/or outdoors are an optimal approach to motivate PwHNC to be more physically active. However, this approach has not yet been sufficiently investigated. In addition, there is little knowledge about the sustainability of (home) training interventions in PwHNC.

As a result, our goal is to develop an exercise program that can be carried out 100% independently and flexibly by PwHNC at home or outdoors. This exercise program should be designed in a way that it can be flexibly adapted to the individual needs of PwHNC. The short and medium-term effects on QoL, the level of physical activity, physical functionality and body composition will be investigated. Here we present the study protocol and the results of the feasibility phase (proof of concept).

## Methods and analysis

### Trial design

The OSHO #94 study is a prospective, multicentre, single-arm, intervention trial.

### Participants, eligibility criteria, and settings

The study is conducted by the Department of Haematology, Oncology and Palliative Care of Rostock University Medical Centre (clinic III, UMR, Germany). Recruitment started on January 1, 2021 at the UMR in cooperation with the Department of Otorhinolaryngology, Head and Neck Surgery ‘Otto Koerner’. The Krukenberg Cancer Centre Halle of the University Hospital Halle has been recruiting since September 20, 2022 and the Department of Otorhinolaryngology, Head and Neck Surgery at the University Medicine Greifswald since March 1, 2023. The study team is supported in the application/recruitment process by the self-help network Kopf-Hals-M.U.N.D.-Krebs e. V. Recruitment should be completed on December 31, 2024.

Patients must meet all of the following inclusion criteria: age ≥18 years, a final coded diagnosis according to ICD: C00-C14, C30-C32 (HNC) in aftercare (after antineoplastic therapy or after completion of rehabilitation, if planned) or with stable remission under immunotherapy and medical clearance of the treating physician, able to walk.

The exclusion criteria are inadequate knowledge of the German language, consent not given, clinically relevant heart failure (NYHA III and IV), myocardial infarction within the last 4 weeks, unstable angina pectoris, higher-grade valvular vitia, uncontrolled cardiac arrhythmias, chronic obstructive pulmonary disease (GOLD III and IV), peripheral arterial occlusive disease (≥Stage III according to Fontaine), diseases that could seriously impair cognitive performance (e.g. dementia, stroke, Wernicke-Korsakoff syndrome), known alcohol dependency and score <24 points on the Mini-Mental State Examination (MMSE).

In accordance with the ethical standards established in the 1964 Declaration of Helsinki and its later amendments, the study protocol (version 1 from September 30, 2020) was approved by the Ethic Committee of the University of Rostock (A2020-0274). Version 2 from June, 13, 2022 and version 3 from July, 19, 2022 was approved by the Ethics Committee of the University of Halle-Wittenberg (2022–067)) and University of Greifswald (BB 117/22). Three protocol modifications have been made and approved so far. These included the extension of the recruitment period twice (amendments from December 2021, and March 2024), the inclusion of PwHNC under palliative care (amendment from July 2022), and the change of deputy study leader (amendment from March 2024). The study is registered at the German Clinical Trials Register (DRKS00023883). All participants will have to sign and date an informed consent form.

### Study procedure and intervention

**Fig 1:**
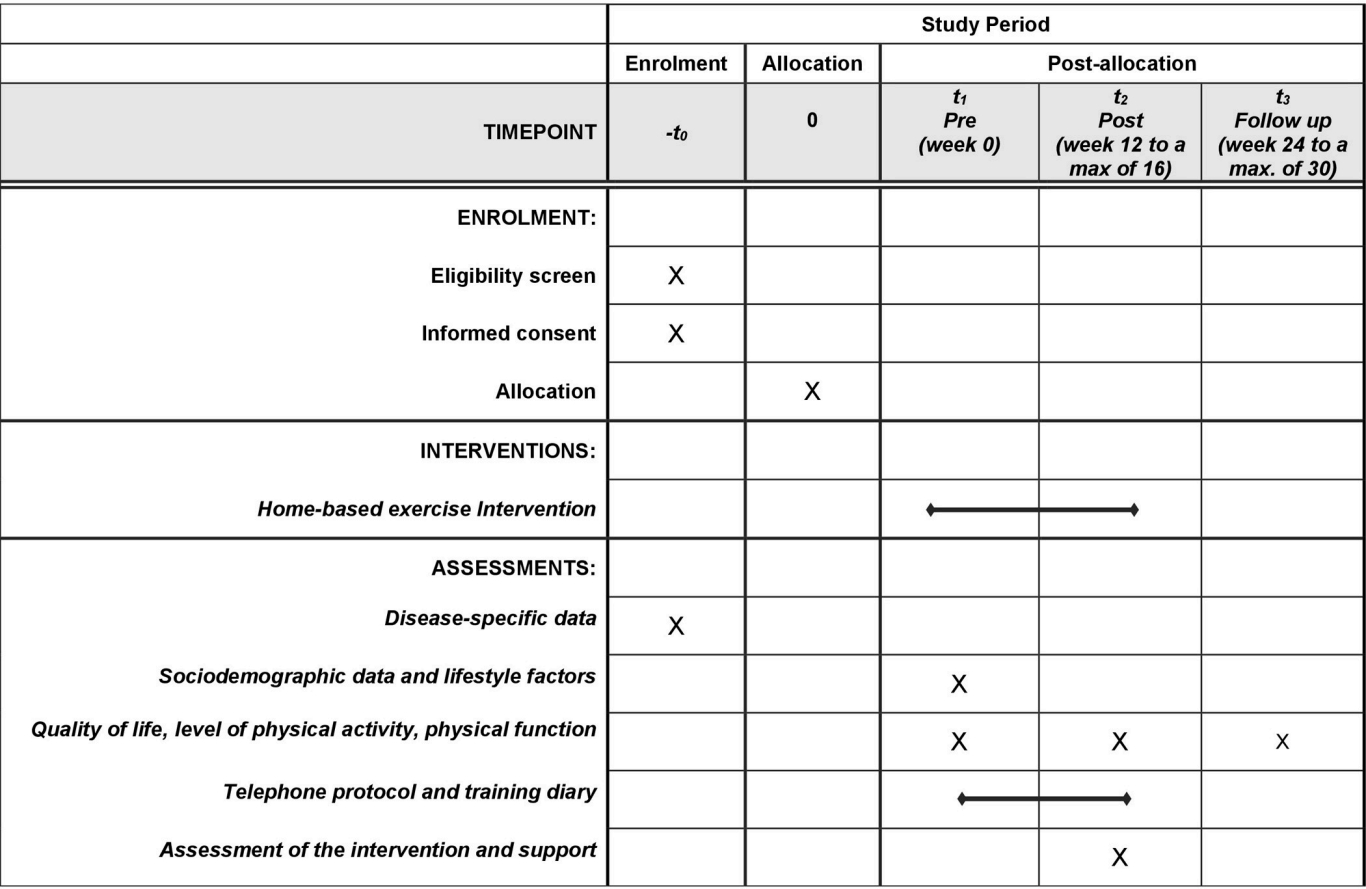
SPIRIT schedule of enrolment, interventions, and assessments.

**Fig 2:**
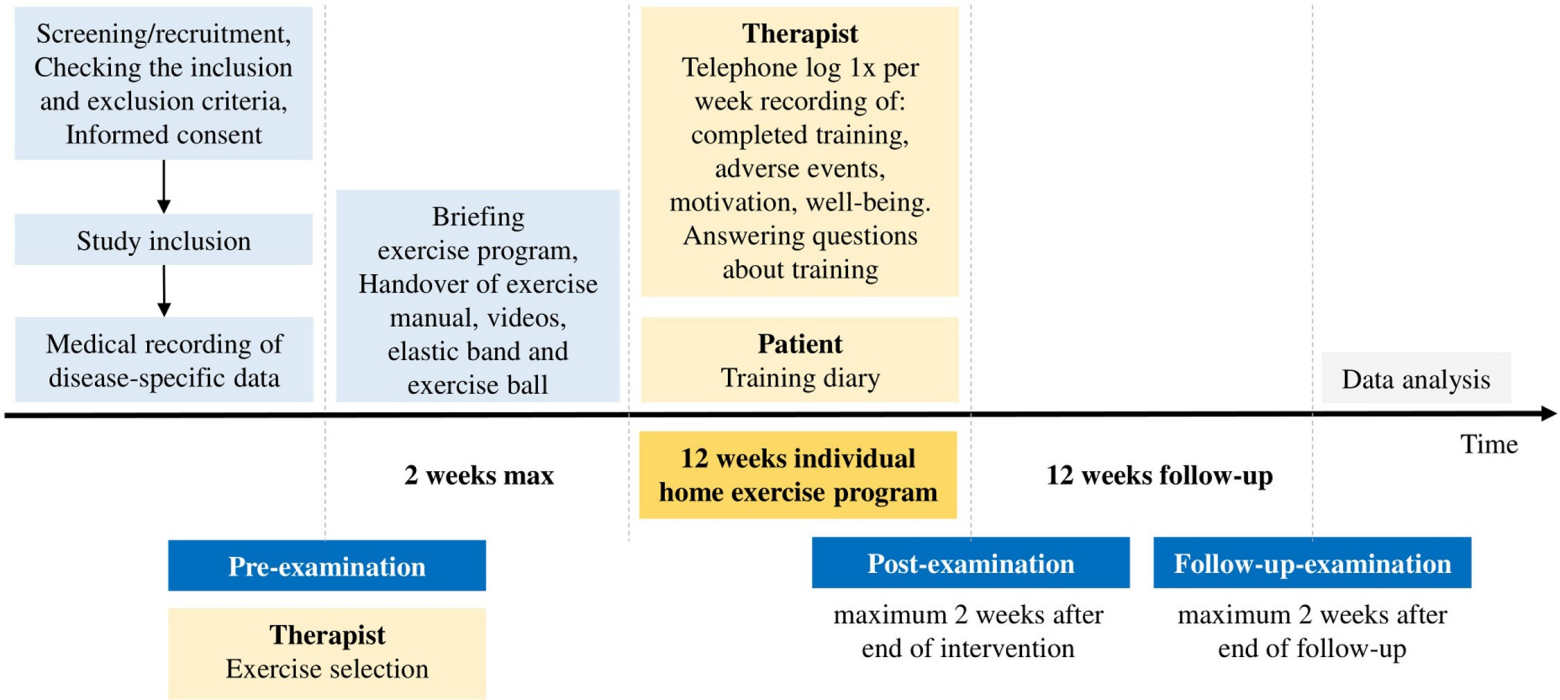
Study procedure OSHO #94.

The SPIRIT schedule of enrolment, interventions, and assessments as well as the schematic study procedure are shown in Figs 1 and 2. Screening of outpatients who come to the consultation is carried out locally at the recruiting study centres. Potential study patients are approached directly by the study team and informed about the study. PwHNC who have become aware of the study through the self-help network Kopf-Hals-M.U.N.D.-Krebs e. V. or through flyers can also contact the study centres. If the patient meets the eligibility criteria, the patient is informed by a physician or sports scientist/physiotherapist. Following written consent by the patient, the pre-examination is administered. Based on the results of this examination and the objectives of the PwHNC, the therapists create an individual training plan. Depending on the patients´ place of residence, time and state of health, they will be introduced to the training program following the pre-examination or at a separate appointment within the next two weeks. Study patients receive the ‴Exercise manual for patients with mouth, jaw, face and throat tumours’” [28] for training at home. This exercise manual containing a total of 90 mobilization, coordination, strengthening and stretching exercises and four exercise programs for training at home. The exercises contained in the manual are based on a previous study conducted at UMR’s Clinic III in 2018/19 [29]. A S1 Table provides an overview of the key elements of the previous study and the current study. In this manual, the exercises recommended by the therapists are marked with a green sticky dot, and contraindicated exercises are marked with a red sticky dot. Video clips for all 90 exercises and the four exercise programs were created in line with the manual. The four exercise program videos have the following content and durations: (1.) Functional gymnastics & balance, time approx. 20 min, (2.) Coordination training, time approx. 12 min, (3.) Mobilization, coordination and strength training with exercise ball, time approx. 22 min, (4.) Training of the shoulder and neck region, time approx. 30 min. If the patients have the appropriate technology (e.g. computer) and wish to do so, the therapists put the recommended individualized exercises in a video clip. This and the four exercise program videos are saved on a USB stick and given to the patients. Patients also receive a free elastic band for strengthening exercises and an inflatable exercise ball (Ø 22 cm) for coordination and strengthening exercises at home. The 12-week individual home exercise program starts immediately after instruction in the exercise program.

The training recommendations/ FITT-criteria (frequency, intensity, time, type) include completion of the individual exercise program or alternatively one of the four exercise videos on at least 3 days per week for 15–30 minutes. The individual training programs consist of a selection of 15–25 exercises. Depending on the participant’s current fitness level and needs, it includes a different number of mobilization, coordination, strengthening and stretching exercises. The recommended duration of each exercise varies between 30 and 60 seconds and from 1 to 3 repetitions/sets. In addition, endurance training, e. g. (Nordic) walking, cycling, swimming, dancing or similar, is recommended at a frequency of 2 to 3 times a week for 30 minutes each time. The training intensity is controlled using the 15-point BORG rating of perceived exertion scale [30], and the recommended range is between 11 (fairly easy) and 15 (hard).

During the 12-week intervention, the therapists contact the patients by telephone once a week and write a telephone protocol. Any questions the patients may have about the training are answered and, if necessary, further training tips are given. In addition to the telephone protocol, patients are asked to keep a training diary for the whole intervention period. Following the 12-week home exercise program, the post-examination takes place. After the post-examination, the patients are left to their own resources on the assumption that they can carry out the exercise program or other physical activities on their own without further instruction (= Follow up, FU). After a further 12 weeks, an FU-examination is carried out in order to be able to make statements about the medium-term effects of the home exercise intervention.

### Outcome measures

The data collection schedule is shown in Table 1.

**Table 1:** Data collection schedule.

Assessments	Pre	Post	FU
**Physician**			
Disease-specific data (questionnaire)	✓		
Tumour location, cancer stage, date of diagnosis, previous medical treatments,			
current therapy phase			
**Patient**			
Sociodemographic data and lifestyle factors (initial questionnaire)	✓		
Age, sex, height, weight, marital status, educational level, professional status,			
tobacco and alcohol consumption, sports history			
Quality of life (QLQ-C30, H&N35)	✓	✓	✓
Level of physical activity (GSLTPAQ)	✓	✓	✓
Assessment of the intervention and support (exit questionnaire)		✓	
Effects of intervention on physical performance, well-being, stress, self-			
esteem, and mood (4-point Likert scale), motivation to train and influence of			
weekly telephone calls (4-point Likert scale), training alone or with others,			
maintaining the training, satisfaction with the training information, use of the			
materials provided			
**Physical function diagnostic**			
Flexibility	✓	✓	✓
temporomandibular joints (incisor distance at maximum mouth opening)			
shoulder joints and cervical spine (active ROM)			
lower back and hamstring muscles (stand and reach test)			
Fall risk (SPPB)	✓	✓	✓
Aerobic performance (6MWT)	✓	✓	✓
Walk distance, RPE (BORG-scale), exercise-induced pain in the legs (CR-10)			
Body composition (BIA)	✓	✓	✓
Fat mass, skeletal muscle mass			
**Therapist and patient: telephone protocol and training diary**			
Weekly completed training (type, frequency and time)	Continuously		
Adverse events in connection with the training			
Weekly motivation of patients to exercise (4-point Likert scale)			
Weekly well-being of the patients (10-point Likert scale)			

Abbreviations: FU, Follow-up; QLQ-C30, Quality of Life questionnaire of cancer patients of European Organization for Research and Treatment of Cancer; H&N35, Quality of Life questionnaire modul of head and neck cancer patients; GSLTPAQ, Godin-Shephard Leisure-Time Physical Activity Questionnaire; ROM, range of motion; SPPB, Short Physical Performance Battery; 6MWT, six-minute-walk-test; RPE, Rating of perceived exertion; CR-10, Category-Ratio-10 scale (0 = no pain at all, 10 = extremely intense pain); BIA, bioimpedance analysis

*Disease-specific data* (tumour location, cancer stage, date of diagnosis, previous medical treatments and current therapy phase) on the study patients are recorded once by the treating/including physicians at the time of study inclusion.

At the three examination appointments–pre (week 0), post (week 12 to a maximum of 16) and FU (week 24 to a maximum of 30)–patient-reported outcomes (PROs) are recorded, and a physical function diagnostic is carried out. The assessment consists of the following measures:

Patient-reported:

- *Sociodemographic data* (age, sex, height, weight, marital status, educational level, professional status), and *lifestyle factors* (tobacco and alcohol consumption, sports history) are recorded using an initial questionnaire. The body mass index (BMI) is calculated (body weight [kg]/height [m^2^]).
- *QoL* is measured using two established questionnaires: (1) EORTC QLQ-C30 version 3.0 questionnaire [31, 32] and (2) the QLQ-HN35 head and neck–specific questionnaire [32]. The EORTC QLQ-C30 is a 30-item cancer-specific questionnaire that has a global QoL scale, 5 functional scales, 3 symptom scales and 6 single items. EORTC QLQ-HN35 is a 35-item module. It contains 7 symptom scales and 6 symptom items. Each scale results in an average score of 0 to 100. A high value on the scale ‘global QoL’ and on the functional scales means a high degree of subjectively perceived health and a high assessment of the QoL or a high degree of performance and function. A high value in the symptom scales correlates with a high degree of complaints and symptoms [31, 33, 34]. No threshold values were set in advance to assess the clinically important difference. The primary outcome is determined by comparing the global QoL score pre- and post-intervention.
- *Level of physical activity* is assessed using the Godin-Shephard Leisure-Time Physical Activity Questionnaire (GSLTPAQ) [35, 36]. The GSLTPAQ is a 4-item self-administered questionnaire. The first three questions seek information on the number of times one engages in mild, moderate and strenuous physical activity bouts of at least 15 min duration in a typical week [37]. Scores derived from the GSLTPAQ include total weekly leisure-time physical activity, called a Leisure Score Index (LSI), in which number of bouts at each intensity is multiplied by 3, 5 and 9 metabolic equivalents and summed. LSI scores can be used for ranking individuals from the lowest to highest physical activity levels [38].
- *Assessment of the intervention and support* is carried out in an exit questionnaire. First, the study participants assess the effects of the intervention on physical performance, well-being, stress, self-esteem, and mood on a 4-point Likert scale. Second, how difficult it was for the PwHNC to motivate themselves to train and what influence the weekly physiotherapy phone calls had on their motivation to train were also recorded on a 4-point Likert scale. Third, it is recorded whether the PwHNC carried out their training alone or with others (e. g. family members). Fourth, the PwHNC are asked whether they will maintain the training. Fifth, the PwHNC were asked whether the information they received about the training was sufficient and, if not, what additional information they would have liked. Sixth, the PwHNC should indicate whether they used the exercise manual, the videos and the small equipment provided for their training.
  Physical function diagnostic:
  Physical function assessments are carried out a maximum of two weeks before the intervention and a maximum of two weeks after the intervention and the FU. These are performed in the same order. The aim is for all tests to be carried out at one location by the same investigator. The assessment consists of the following measures:
- *The flexibility of the temporomandibular joints* is measured using the distance between the incisors (cm) determined with a ruler at maximum mouth opening.
- *The flexibility of the shoulder joints and the cervical spine* is measured by the active range of motion (ROM) in the sagittal, frontal, and transversal planes using a manual goniometer. The measurements are carried out starting from the maximum ROM away from the body to the end position close to the body (°).
- *The flexibility of the lower back and hamstring muscles* is assessed with the stand and reach test. Subjects stand with closed/stretched legs and hold one [39] hand covering the other. The trunk is slowly flexed, and the distance between the hands and the ground (which can be hold for 2 s) is measured (cm) [39].
- *Fall risk* is evaluated by using the short physical performance battery (SPPB). The SPPB has been shown to have predictive value for the assessment of mortality risk, nursing home admission and disability [40]. The SPPB is a group of measures that combines the results of balance tests, gait speed and repeated chair stands. The scores range from 0 (worst performance) to 12 (best performance).
- *Aerobic performance* is assessed using the 6-minute walk test (6MWT) [41]. The primary measure is the walk distance (m) achieved within 6 minutes. The test is carried out on a 40-m long straight track (a mark is placed every 10 m) on solid ground. After the 6MWT, the participants are asked to rate their perceived exertion (RPE) using the 15-point Borg scale (6 = really, really easy, 20 = maximum effort) [30] and their exercise-induced pain in the leg muscles using a Category-Ratio (CR)-10 scale (0 = no pain at all, 10 = extremely intense pain) [42].
  Following completion of the intervention, the telephone protocols and training diaries are evaluated with regard to the following parameters:
- *Compliance with regard to training recommendations*: the documented training sessions are evaluated with regard to the average weekly training frequency and time, separately for individual exercise programs and endurance training.
- *Adverse events* in connection with the intervention that are mentioned by the participants are recorded.
- *The patients’ motivation to train* is recorded over the 12-week intervention using a 4-point Likert scale (1 = not at all, 4 = very much).
- *Weekly well-being* is documented and analysed on a 10-point Likert scale (0 = very poor, 10 = very good).

If possible, the reason for dropping out is asked in the event of early termination of studies.

### Sample size calculation

The sample size calculation for the trial is based on the results of Felser et al. [28]: global QoL pre: 50.1 ± 16.4; post: 58.3 ± 16.2; r = 0.618 resulting in an effect size of 0.5755, which is detectable on a confidence level of 1 − α = .95 (2-sided) with n = 42 patients (paired t-test of mean difference equal to zero based on sd of differences from sd_pre, sd_post and correlation; nQuery® Advisor 7.0 Statistical Solutions Ltd., Boston, MA, USA). Assuming a 20% dropout rate based on our experience (proof of concept), a total of N = 53 patients is required to be included in the study.

### Feasibility ‐ proof of concept

The proof of concept (n = 25) examines the feasibility of the study design including the estimation of the dropout rate during the intervention. The estimated dropout rate is given with the respective 95% confidence interval (95%CI) for feasibility. Feasibility phase involves a maximum dropout rate of 30%. With a dropout rate below 30%, the study should continue recruitment without changing the study design and the sample size calculation was to be adjusted. Otherwise, it was planned to stop the recruitment and modify the study design.

Further outcomes for assessing the feasibility and acceptance of the intervention are patient characteristics, the completed training volume, adverse events associated with the training, satisfaction with the training information, use of the exercise materials and the influence of the weekly telephone calls during the intervention phase on motivation. All data was analysed descriptively.

### OSHO #94 ‐ statistical analysis

Quantitative variables (i.e. QoL) are presented as mean ± standard deviation (sd) or in case of non-normality, as median (Q1, Q3), ranging from minimum to maximum (min to max); qualitative ones as relative frequency of their occurrence % and absolute (n). Missing data are indicated but not included in the calculation of percentage. The normal distribution of the data is checked using the Shapiro-Wilk test.

QoL, physical activity level, physical functionality and body composition will be measured at three time points (pre, post, FU). Subgroup analyses are planned according to the patient characteristics, including men vs. women, time after diagnosis, sports beginners vs. PwHNC with sports experience, low vs. high physical performance/ physical activity level/ QoL and centres. Correlation analyses are carried out to examine the strength and direction of relationships between quantitative variables. QoL score at different time points will be fitted to a Generalized Linear Mixed Model supporting the repeated measurement design. Regression analysis will be used to quantify the influence of several predictors like training volume, symptom burden, centre on QoL, controlled for age, gender and time after diagnosis. Secondary outcomes as physical function diagnostics (flexibility [cm], aerobic performance [m], body composition [kg]) will be analysed on ranks. The test of Friedman, followed pairwise by Wilcoxon tests will be performed. The level of significance will be Bonferroni-adjusted.

A *p*-value of <0.05 is considered significant. All data will be analysed using IBM^®^ SPSS^®^.

### Patient involvement

UMR’s clinic III works closely with the local self-help group and the German self-help network Kopf-Hals-M.U.N.D.-Krebs e. V. to educate PwHNC and their families about physical activity and sports. The local self-help group contributed from the beginning to the design and implementation of the OSHO #94 study by helping to create the exercise manual [28] and videos. Both the local self-help group and the self-help network Kopf-Hals-M.U.N.D.-Krebs e. V. inform PwHNC about OSHO #94 via social media and information events. On “patient days”, often organized by the clinical institutes in cooperation with the self-help group, study participants report on their experiences with the exercise program, and interim results are presented and discussed together. It may therefore be possible to recruit new study participants via patient days.

## Results of feasibility (proof of concept)

Between January 2021 and February 2023, 25 PwHNC were included in the study. The proportion of men was 52% (n = 13). The median age was 66 (61, 72), ranging from 20 to 85 years. A higher education degree (>10 years) was held by 60% (n = 15) of the participants, 56% (n = 14) were non-smokers and 72% (n = 18) stated that they had been active in sports before the disease. The most common tumour location was the oropharynx (32%, n = 8), followed by the oral cavity (28%, n = 7). Participants were first diagnosed between 2006 and 2022. At the time of study participation, 92% (n = 23) were in complete remission. Further details on the socio-demographic, lifestyle and clinical data of the study participants can be found in Table 2.

**Table 2:** Sociodemographic and clinical data (n = 25).

Patients characteristics	Category	Values
Gender	Women	12 (48%)
	Men	13 (52%)
Age [years]		66 (61, 72)
Body Mass Index [kg/m²]		23.8 (21.3, 26.6)
School education [years]	<10	5 (20%)
	10	5 (20%)
	>10	15 (60%)
Family status	Single	8 (32%)
	Married/ living with a partner	17 (68%)
Professional status	Working	4 (16%)
	Retired	20 (80%)
	Other	1 (4%)
Tobacco consumption	Smoker	2 (8%)
	Ex-Smoker	9 (36%)
	Non-Smoker	14 (56%)
Current alcohol consumption	Yes	17 (68%)
	No	8 (32%)
Active in sports before cancer diagnosis	Yes	18 (72%)
	No	7 (28%)
Time after initial diagnosis [months]		36 (15, 99)
Tumour location	Oropharynx	8 (32%)
	Mouth cavity	7 (28%)
	Others	10 (40%)
Cancer stage	I	8 (32%)
	II	2 (8%)
	III	5 (20%)
	IV	10 (40%)
Current therapy situation	Complete remission	23 (92%)
	Under immunotherapy	1 (4%)
	Others*	1 (4%)
Treatment	Surgery only	6 (24%)
	RT or RCT only	3 (12%)
	Surgery and RT / RCT	16 (64%)

Data are presented as the number of participants (%) for categorical variables and as median (Q1, Q3) for continuous variables.

Abbreviations: n, number of patients; RT, radiotherapy; RCT, combined radio-chemotherapy

*Clinically tumour-free, positron emission tomography (PET)-CT still pending on inclusion

### Dropout rate

A total of 22 out of 25 participants completed the intervention. Three participants (12%) discontinued the intervention prematurely for health reasons (operations, psychological stress due to unclear findings). Data from 21 participants were analysed due to the fact that one participant underwent surgery between the end of the intervention and post-examination, giving a dropout rate of 16% in phase A with 95%CI [.016; .304].

### Secondary outcomes

#### Compliance with regard to training recommendations

In median, the participants completed the individual exercise program 3.4 (2.5, 5.6) times per week, ranging from 1.3 to 6.8. The median training time was given as 98 (85, 150) min per week (35 to 304). Endurance training was completed an average of 2.8 (1.9, 4.9) times per week (0 to 6.7), with the median training time per week reaching 167 (86, 247) min (0 to 497 min). Overall, the median weekly training time of the participants was 268 (210, 328) min per week, ranging from 63 to 753 min per week.

#### Adverse events

A total of three participants (12%) reported adverse events that could be related to the exercise. One of these was pain in the Achilles tendon area, one patient complained of knee pain after the first training session and one patient reported pain in the shoulder.

#### Satisfaction with the information about the training

All patients (100%) stated that the information they received about the training was sufficient.

#### Use of the exercise materials

The exercise manual was used by 76% (n = 16) of the participants and the USB stick with the training videos by 62% (n = 13), with 32% (n = 8) stating that they used both. The small equipment (elastic band and inflatable exercise ball) was used by 86% (n = 18) of the participants.

#### Influence of telephone calls on motivation to train

A total of 86% (n = 18) of participants stated that the weekly calls from the therapists had a positive influence (29% very, 24% quite, 33% somewhat) on motivation to train.

## Discussion

To our knowledge, the OSHO #94 study is the first study to investigate the short- and medium-term effects of 100% individualized home training in PwHNC after completion of cancer therapy or in a stable situation under immunotherapy. In contrast to previous studies [29, 43–61], participants do not receive an exercise program with defined FITT criteria that apply equally to all intervention participants, but rather exercise recommendations based on the current physical activity guidelines for cancer survivors [62]. The participants can perform the endurance training according to their preferences, and the recommendations regarding strength and mobility are adapted to the individual’s needs/deficits. OSHO #94 is thus pursuing the approach of transferring the knowledge previously generated primarily in randomized controlled trials (RCT) regarding the effectiveness of targeted exercise interventions in PwHNC [43, 45, 47–60, 63, 64] to ‘real-world’ care.

The home-based training approach was chosen (i) because, in our view, it comes closest to the preferences of PwHNC [25–27]. (ii) In addition, home-based training programs potentially offer all PwHNC the opportunity to participate, regardless of their place of residence. (iii) In contrast to temporary rehabilitation programs, home-based exercise programs can be continued indefinitely, which can increase QoL and physical activity levels not only in the short but also in the medium term. (iv) The recruiting centres (Rostock, Halle, Greifswald) are located in the northeast of Germany, a rather sparsely populated region with poor infrastructure. Specific exercise programs for people with cancer are scarce in this region, making it difficult to refer PwHNC to community-based programs. (v) Home exercise programs are cost-effective, as there are no membership fees and no travel costs, and they also relieve the burden on the healthcare system/caregiver. (vi) By eliminating the need to travel, home exercise programs are less time consuming and can potentially reduce barriers to exercise [23]. Although home exercise programs for PwHNC offer various advantages over group training, feasibility, effectiveness and sustainability have been insufficiently studied, and further research, especially with greater attention to implementation science aspects, seems warranted.

Previous studies with home exercise approaches [65–67] and the feasibility results of OSHO #94 –low dropout and low number of reported adverse events–showed that the chosen home exercise approach is safe to implement. The dropout observed in the feasibility study was clearly below our expectations, which were influenced by the results of Cnossen et al. [68]. Among other things, the authors investigated the treatment adherence of PwHNC who participated in a guided home-based prophylactic exercise program during treatment. Adherence was 38% after 12 weeks. The authors saw the main reason for the decrease in adherence in the increasing negative effects of the treatment, which is only to be expected in exceptional cases due to the inclusion criteria in OSHO #94. Since the estimated dropout rate of the OSHO #94 trial is identical to that of our previous study, which we conducted in a group setting, we consider the assumption of 20% dropout to be realistic. Therefore, we adjusted our original calculation of the sample size with a calculated dropout of 30% (N = 60 PwHNC) to 20% (N = 53 PwHNC).

Although, in line with our expectations, the range in terms of training volume (frequency and time) in the feasibility phase is very large, the majority of the PwHNC included are adhering to the exercise recommendations or even exceeding them. One reason for this could lie in the included cohort itself, as the demographic data show that so far mainly PwHNC with a high level of education and a history of sport have been included. The fact that the willingness to participate in an exercise intervention is higher here is consistent with the results of Buffart et al. [69]. In addition, it is well known that education/social status and health (behaviour) correlate with each other in the general population [70–72]. Another reason could be the study design, which involves weekly telephone calls between therapists and study participants. According to the feasibility results, these telephone calls increase the motivation to exercise in four out of five participants. The results of the FU study will show the influence of the absence of telephone calls on physical activity. Conducting the FU study three months after the end of the intervention will allow an initial assessment of whether the guided home exercise program has an impact on the physical activity behaviour and QoL of PwHNC and can increase the physical activity level in the medium term. It should be noted at this point that the findings of this study relate to the enrolled sample. In order to properly evaluate the benefits of such an intervention (individualized home training), larger sample sizes and studies on other populations are required.

Representatives of the target population were involved in the development of the OSHO #94 study by jointly developing and creating the exercise manual and the training videos. Since the small devices have also been used to a high degree by the current participants, it was decided that the study design would be adhered to, even if the demographic data/lifestyle parameters of the PwHNC included so far do not reflect the typical PwHNC (male, smoker, low level of education). As access to the study was deliberately chosen to be low-threshold–direct approach of the PwHNC or contact via telephone or email from the patients, three on-site appointments over a period of six months with travel costs covered, no costs for materials and support–the results of the study will provide information on which PwHNC are addressed with the approach of guided home exercise.

The selected assessment in the OSHO #94 study corresponds to international standards and includes instruments that were/are regularly used in studies with PwHNC and thus enable comparability of the results. The EORTC QLQ-C30 is used to assess the primary outcome, the QoL, as recommended by Burgos-Mansilla et al. [15]. This global instrument appears to be more sensitive to changes in general QoL than to more specific instruments. The GSLTPAQ, an instrument widely used in oncology research, is also used to record and classify physical activity. In accordance with the recommendations of Amireault et al. [36] the original form of the GSLTPAQ is used, and the LSI is used for interpretation.

### Limitations

The design of the OSHO #94 study has some limitations, which are first related to the single-arm design, which does not allow a comparison of the results with a control group (usual care). Second, due to the diverse dissemination of information about the study at three study centres, via regional self-help groups as well as the Germany-wide patient network, social media and flyers, no statement on the recruitment rate is possible. In previous studies with PwHNC, recruitment rates ranged from 20% to 32.3% [17]. With regard to recruitment, it should be mentioned that the number of recruiting centres is lower than originally planned due to the COVID-19 pandemic. As a result, recruitment is slower and the recruitment period had to be extended. Third, the LSI of the GSLTPAQ is used to interpret physical activity levels [36]. Since PROs for physical activity only correspond to objective measurement data, for example, that collected with accelerometers, to a limited extent [73], this can lead to incorrect classifications of PwHNC. Since the GSLTPAQ is used at all three measurement times, it can be assumed that the ‘errors’ are constant, and thus changes in the activity level can be reliably mapped. The OSHO #94 study will allow us to better understand the effectiveness of guided home exercise programs at the individual level and to evaluate processes to support future implementation and sustainability.

## Supporting information

S1 Table Key elements previous and current study. (PDF)

S1 Dataset Feasibility OSHO #94. (XLSX)

S1 File Study protocol OSHO #94_german. (PDF)

S2 File Study protocol OSHO #94_english. (PDF)

S1 Checklist SPIRIT 2013 checklist: Recommended items to address in a clinical trial protocol and related documents*. (DOC)
