# Supplementary material for: Feasibility of individualized home exercise programs for patients with head and neck cancer–study protocol and first results of a multicentre single-arm intervention trial (OSHO #94)
Source: PLoS One. 2024 Aug 22;19(8):e0301304. doi: 10.1371/journal.pone.0301304 (PMC11341025; doi:10.1371/journal.pone.0301304)
Supplement: S1 Table — (PDF) [file pone.0301304.s001.pdf]

Table S1. Comparison of the previous study and the current study (OSHO #94)

	**Previous study**	**Current study (OSHO #94)**
Primary objective	*Development* and evaluation of a specific home exercise program for PwHNC[1], the basis for an exercise manual[2]	*Evaluation* of an individualized home exercise training for PwHNC
Trial design	prospective, single arm intervention	prospective, single arm intervention
Number of recruiting centres	1	3
Setting	group training	individual training
Location and time	fixed	flexible
FITT-Criteria (recommendations) for the specific exercise program		
Frequency [times per week]	2	at least 3
Intensity [BORG-scale]	11 to 15	11 to 15
Time [min per session]	50	at least 15 – 30
Type	multimodal	multimodal
Training program	exclusively exercises that the PwHNC could carry out independently at home: Ø 14 min mobilization, Ø 9 min coordination, Ø16 min strengthening, Ø 5 min stretching, Ø 4 min relaxation	selection of 15 – 25 exercises according to the restrictions and the goals (for mobilization, coordination, strengthening, stretching) + endurance training 2 to 3 times a week for 30 minutes each time[3]
Duration [weeks]	12	12
Follow up	no	yes, 12 weeks

Abbreviation: PwHNC, Patients with head and neck cancer

[1] The results of the previous study are published at: https://doi.org/10.1177/1534735420918935

[2] The exercise manual has the ISBN 978-109909608 and can be ordered via Amazon

[3] In accordance with the exercise guidelines for cancer survivors of the American College of Sports Medicine (Campbell KL, et al. Exercise Guidelines for Cancer Survivors: Consensus Statement from International Multidisciplinary Roundtable. Med Sci Sports Exerc 2019; 51(11):2375–90.)
