# Supplementary material for: Feasibility of individualized home exercise programs for patients with head and neck cancer–study protocol and first results of a multicentre single-arm intervention trial (OSHO #94)
Source: PLoS One. 2024 Aug 22;19(8):e0301304. doi: 10.1371/journal.pone.0301304 (PMC11341025; doi:10.1371/journal.pone.0301304)
Supplement: S1 File — (PDF) [file pone.0301304.s003.pdf]

Checkliste
(nicht AMG-Studie)

---

1. Basisdaten

1.1 Vollständiger Titel der Studie

Individuelles Heimtraining für Patienten mit Kopf-Halstumoren – eine multizentrische Studie zur Verbesserung der Lebensqualität (HeiKo-Studie)

1.2 Studienleiter/Studienleiterin vor Ort
(Vor- und Zuname, Beruf, Akademischer Titel, Anschrift, Telefon, Fax, Email)

Dr. phil. Sabine Felser, xxx
Stellvertretende Studienleiterin: Dr. med. Christina Große-Thie, xxx

1.3 vor Ort an der Studie beteiligte Institute/Kliniken/Praxen (Anschrift, Telefon)

Klinik III (Hämatologe, Onkologie, Palliativmedizin) Universitätsmedizin Rostock, xxx
Klinik und Poliklinik für Mund-, Kiefer- und Plastische Gesichtschirurgie, UMR (Ansprechpartner: Dr. med. Jan Liese)
Klinik und Poliklinik für Hals-Nasen-Ohrenheilkunde, Kopf- und Halschirur-gie, UMR (Ansprechpartner: Dr. Daniel Strüder)
Studienzentrale der Klinik III, Hämatologie, Onkologie und Palliativmedizin, UMR (Leiterin: Dr. med. Brigitte Kragl)

1.4 Wie wird die Studie finanziert? Kostenträger? Sponsor?

Zuwendung der OSHO e. V. (Anschubfinanzierung), Drittmittel des Projektes „Bewegungstherapie während und nach der Krebstherapie – ein Projekt zur Stärkung der Lebensqualität“ gefördert durch die Krebsgesellschaft M-V e. V. sowie aus Drittmitteln der ZIM III

1.5 Handelt es sich um eine multizentrische Studie?

ja

1.6 Voten anderer Ethikkommissionen

Wurde bereits bei einer anderen Ethikkommission in Deutschland ein Antrag gleichen Inhalts gestellt?

nein

1.7 Welche speziellen Gesetze/Verordnungen müssen Sie bei Ihrer Studie beachten?

☐ Medizinproduktegesetz	☐ Strahlenschutzverordnung
☐ Röntgenverordnung	☐ Transfusionsgesetz
☐ Transplantationsgesetz	☐ Embryonenschutzgesetz
☐ Gentechnikgesetz	☑ Bundesdatenschutzgesetz / Landesdatenschutzgesetz

Andere:

1.8 Angaben zu wirtschaftlichen und anderen konfligierenden Interessen der Studienleitung im Zusammenhang mit der Studie

keine

2. Kurzcharakterisierung der Studie

2.1 Art der Prüfung

- ☐ bevölkerungsbezogene Epidemiologie
- ☐ klinische Epidemiologie
- ☐ Prüfung eines diagnostischen Testes / Verfahren
- ☐ Prüfung eines Behandlungsverfahrens
- ☐ Erprobung/Entwicklung medizinischer Geräte
- ☐ Forschung an/mit Biomaterialien
- ☐ Prognostische Studie
- ☑ Sonstige, und zwar: Trainingsinterventionstudie

2.2 Studiendesign

- ☐ Querschnittstudie
- ☐ Fall-Kontrollstudie
- ☑ Kohortenstudie
- ☐ Experimentelle Studie
- ☐ Kontrolliert
- ☐ Randomisiert
- ☐ Verblindet

2.3 Studienteilnehmerinnen/-teilnehmer

Patienten der UMR und kooperierender Kliniken mit Mund-, Kiefer-, Gesichts- und Halstumoren

Anzahl insgesamt: 60 davon weiblich: 30 mänlich: 30

Begründen Sie bitte, inwieweit die gewählte Geschlechterverteilung zur Feststellung möglicher geschlechtsspezifischer Unterschiede (z.B. in der Wirksamkeit der Behandlung, der Unbedenklichkeit des Medizinproduktes) angemessen ist.

3. Schutz und Sicherheit der Studienteilnehmerinnen und –teilnehmer

3.1 Beschreibung des Verfahrens zur Rekrutierung von Studienteilnehmerinnen und –teilnehmer

Ansprechen von Patienten in der Sprechstunde und über Plakate, Flyer

Dauer der Rekrutierung (Materialien wie Anzeigen, Flyer etc. beilegen)

12 Monate

3.2 Schilderung des Vorgehens zur Information und Erlangung der informierten Einwilligung zur Studienteilnahme der Studienteilnehmerinnen/-teilnehmer, ggf. der Eltern oder des gesetzlichen Vertreters(wer wird informieren und wann, Erfordernis eines gesetzlichen Vertreters / Zeugen)

Patienten werden vor der Befragung mündlich und schriftlich mittels Patienteninformation aufgeklärt, evtl. auftretende Fragen werden beantwortet

3.3 Besonders schutzbedürftige Personen:

Nehmen an der Studie Personen unter 18 Jahren teil? Ja ☐ Nein ☑

Nehmen nicht-einwilligungsfähige Erwachsene teil? Ja ☐ Nein ☑

3.4 Begründung für den Einschluss von besonders schutzbedürftigen Personen

(wie z.B. gesunde bzw. kranke Minderjährige, nichteinwilligungsfähige Erwachsene)

Warum kann die Studie nicht an einwilligungsfähigen Erwachsenen durchgeführt werden?

Es werden keine besonders schutzbedürftigen Personen eingeschlossen

3.5 Von wem werden die Studienteilnehmerinnen/ - teilnehmer vor, während und nach der Studie ärztlich betreut?

(z.B. Abstimmung mit dem Hausarzt /oder Hausärztin? Kontrolle anderer Medikationen? Kontrolle von Blutspiegeln?)

Die Studienteilnehmer befinden sich in der Tumornachsorge und werden diesbezüglich von ihrem onkologischen Behandler-Team betreut (kein Einfluss durch die Studie).

3.6 Erhalten die teilnehmenden Personen eine Aufwandsentschädigung bzw. eine Bezahlung?

☑ Nein ☐ Ja

Wenn ja, warum und in welcher Höhe?

3.7 Ist zugunsten der Studienteilnehmerinnen/ - teilnehmer eine Versicherung (evt. auch Wegeversicherung) abgeschlossen worden?

nein

4. Dokumentation, Auswertung, Berichterstattung

4.1 Welche art der Dokumentation ist vorgesehen? (Dokumentationsbögen ggf. beifügen)

Frage- bzw. Dokumentationsbögen (siehe Anhang)

4.2 Ist ein Monitor vorgesehen?

Nein ☑ Ja ☐

Wenn ja, wie soll durch ihn die Kontrolle der Daten erfolgen unter gleichzeitiger Einhaltung des Datenschutzes/ der Schweigepflicht?

4.3. Ist die Mitarbeit einer Statistikerin/ eines Statistikers vorgesehen?

Nein ☐ Ja ☑

4.4 Vorgesehene Maßnahmen zum Schutze der erhobenen Daten

Patienten werden pseudonymisiert

4.5 Sind Abbruchkriterien festgelegt worden?

- für die einzelnen Studienteilnehmerinnen / - teilnehmer Nein ☐ Ja ☑

- für die Beendigung der gesamten Studie Nein ☑ Ja ☐

Wenn ja, welche?

- Wenn der Studienteilnehmer körperlich und psychosozial nicht dazu in der Lage ist, an der Studie weiter teilzunehmen (z. B. Auftreten absoluter Kontraindikationen) oder seine Einwilligung zurückzieht.

5. Abwägung des Schaden/Nutzenpotentials

5.1 Schaden und Risiken

Welche Arten sind die möglichen Risiken, Beeinträchtigungen, Belastungen oder sonstige Nachteile für die an der Studie teilnehmenden Personen?

Es werden keine Nachteile durch die Studie erwartet, da das Trainingsprogramm dem Belastungsprofil des Patienten angepasst wird. Im Protokoll sind die mögliche Risiken und Belastungen in Kap. 7 aufgeführt.

Inwieweit weichen ggf. Studienbezogene Maßnahmen von der üblichen Routinebehandlung ab?
Gegenwärtig erhalten onkologische Patienten routinemäßig keine strukturierte Onkologische Bewegungstherapie. Die eigentliche routinemäßige onkologische Therapie und Nachsorge wird durch die Studie nicht beeinträchtigt.

5.2 Nutzen und Chancen

Welche Art ist der zu erwartende Nutzen?

☑ Eigennutzen (für die teilnehmenden Personen selbst)

☑ Gruppennutzen (zukünftig für gleich erkrankte Personen)

☑ Fremdnutzen (für die Heilkunde bzw. die Wissenschaft)

5.3 Begründen Sie bitte, warum der mögliche Schaden im Verhältnis zu dem zu erwartenden Nutzen Ihrer Ansicht nach vertretbar ist.

Der Nutzen des allgemeinen Wohlbefindens und der Stärkung der Muskulatur und Koordination überwiegt den möglichen Belastungen wie z. B. Muskelkater.

6. Unterschriften und Erklärung

6.1 Stellungnahme und Unterschrift der Direktorin/ des Direktors der Klinik bzw. des Institutes (mit Stempel)

Mit der Durchführung der unter 1.1 genannten Studie bin ich einverstanden. Ich bestätige, dass die Ressourcen (Anzahl und Qualifikation der Mitarbeiter, Infrastruktur, Geräte und Räumlichkeiten) für eine erfolgreiche Durchführung vorhanden sind.

Außerdem bestätige ich, dass die Rekrutierung der Studienteilnehmerinnen / -teilnehmer nicht durch konkurrierende Studien gefährdet wird.

(Stempel)

Rostock, den

____

Unterschrift

Individuelles <u>Hei</u>mtraining für Patienten mit <u>Ko</u>pf-Halstumoren – eine multizentrische Studie zur Verbesserung der Lebensqualität

HeiKo-Studie (OSHO #94)

Version 1 vom 30.09.2020

**Sponsor:**	Prof. Dr. Christian Junghanß<br>xxxx
**Studienleiterin:**	Dr. phil. Sabine Felser<br>xxx
**Stellvertretende Studienleiterin:**	Dr. med. Christina Große-Thie<br>xxx
**Datenmanagement:**	Leiterin: Dr. med. Brigitte Kragl<br>xxx

Die Informationen in diesem Prüfplan sind streng vertraulich zu behandeln. Sie dienen nur zur Information des Sponsors, der Prüfer, der Studienmitarbeiter, der Ethikkommission, der Behörden und der Patienten. Dieser Prüfplan darf ohne Zustimmung des Sponsors oder der Projektleiterin nicht an Dritte weitergegeben werden.

I. Unterschriften

Dr. phil. Sabine Felser<br>(Studienleiterin)	______________________<br>Unterschrift	08.10.2020<br>Datum
Dr. med. Christina Große-Thie<br>(stellvertretende Studienleiterin)	______________________<br>Unterschrift	08.10.2020<br>Datum

II. Synopse

**Titel der Studie**	Individuelles Heimtraining für Patienten mit Kopf-Halstumoren – eine multizentrische Studie zur Verbesserung der Lebensqualität (HeiKo-Studie)
**Studienleiter**	**Studienleiterin: Dr. phil. Sabine Felser**<br>xxx<br>**Stellvertretende Studienleiterin**: **Dr. med. Christina Große-Thie**<br>xxx
**Beteiligte Einrichtungen**	• Klinik III (Hämatologie, Onkologie, Palliativmedizin) (Direktor: Prof. Dr. med. Christian Junghanß)<br>• Klinik und Poliklinik für Mund-, Kiefer- und Plastische Gesichtschirurgie, UMR (Ansprechpartner: Dr. med. Jan Liese)<br>• Klinik und Poliklinik für Hals-Nasen-Ohrenheilkunde, Kopf- und Halschirurgie, UMR (Ansprechpartner: Dr. Daniel Strüder)<br>• Studienzentrale der Klinik III, Hämatologie, Onkologie und Palliativmedizin, UMR (Leiterin: Dr. med. Brigitte Kragl)<br>• Institut für Biostatistik und Informatik in Medizin und Alternsforschung (IBIMA) der UMR (Ansprechpartnerin: PD Dr. Änne Glass)<br>• Kooperationspartner: Kliniken/Praxen der OSHO e. V.
**Fragestellungen**	HNSCC-Patienten leiden oft aufgrund der Lage des Tumors und der intensiven lokalen Therapieverfahren an einer hohen chronischen Symptombelastung, welche die Lebensqualität (QoL) beeinträchtigt. Es gibt starke Belege für die Vorteile von körperlicher Aktivität in Bezug auf die Symptomlinderung und den Ausgleich von Funktionsstörungen. So kann durch körperliche Aktivität die Körperzusammensetzung, die muskuläre Fitness, Schmerzen, die Flexibilität und die QoL positiv beeinflusst werden. Aus Befragungen ist bekannt, dass viele HNSCC-Patienten ein Training zu Hause, allein und mit moderater Intensität bevorzugen. Um die Machbarkeit und die Effekte eines individuellen Heimtrainings bei HNSCC Patienten auf verschiedenen Graden der QoL zu evaluieren, sollen in dieser Studie folgend Fragen beantwortet werden:<br><br>**Primäres Outcome:**<br>Phase I (Machbarkeit)<br>• prozentualer Anteil der Patienten, die die Trainingsintervention im Home-based Setting über 12 Wochen durchführen<br>Phase II (Lebensqualität)<br>• Veränderung des globalen QoL-Scores (EORTC-QLQ-30) nach einer 12-wöchigen Trainingsintervention im Home-based Setting<br><br>**Sekundäre Outcomes:**<br>• Gründe für einen vorzeitigen Abbruch der 12-wöchigen Trainingsintervention<br>• Häufigkeit und Dauer des Trainings im Home-based Setting im Durchschnitt pro Woche (Trainingsumfang)<br>• Mittelfristige Veränderung der QoL nach einer 12-wöchigen Trainingsintervention im Home-based Setting

	• Kurzfristige Veränderung der Beweglichkeit nach einer 12-wöchigen Trainingsintervention im Home-based Setting<br>• Mittelfristige Veränderung der Beweglichkeit nach einer 12-wöchigen Trainingsintervention im Home-based Setting<br>• Kurzfristige Veränderung der körperliche Aktivität nach einer 12-wöchigen Trainingsintervention im Home-based Setting<br>• Mittelfristige Veränderung der körperlichen Aktivität nach einer 12-wöchigen Trainingsintervention im Home-based Setting<br>• Kurzfristige Veränderung der Gleichgewichtsfähigkeit /Gangsicherheit nach einer 12-wöchigen Trainingsintervention im Home-based Setting<br>• Mittelfristige Veränderung der Gleichgewichtsfähigkeit /Gangsicherheit nach einer 12-wöchigen Trainingsintervention im Home-based Setting<br>• Kurzfristige Veränderung der aeroben Ausdauerleistungsfähigkeit nach einer 12-wöchigen Trainingsintervention im Home-based Setting<br>• Mittelfristige Veränderung der aeroben Ausdauerleistungsfähigkeit nach einer 12-wöchigen Trainingsintervention im Home-based Setting<br>• Kurzfristige Veränderungen in der Körperzusammensetzung nach einer 12-wöchigen Trainingsintervention im Home-based Setting<br>• Mittelfristige Veränderungen in der Körperzusammensetzung nach einer 12-wöchigen Trainingsintervention im Home-based Setting<br>• Zusammenhänge zwischen dem Trainingsumfang während der 12-wöchigen Trainingsintervention und den Effekten hinsichtlich QoL<br>• Zusammenhänge zwischen dem Trainingsumfang während der 12-wöchigen Trainingsintervention und den Effekten hinsichtlich der Funktionalität<br>• Zusammenhänge zwischen dem Trainingsumfang während der 12-wöchigen Trainingsintervention und den Effekten hinsichtlich der Körperzusammensetzung<br>• Zusammenhänge zwischen Alltagsaktivität und QoL<br>• Zusammenhänge zwischen Alltagsaktivität und Funktionalität<br>• Zusammenhänge zwischen Alltagsaktivität und Körperzusammensetzung<br>• Unterschiede bei der Wahrnehmung der subjektiven Effekte des Heimtrainings in Abhängigkeit vom Geschlecht
**Studiendauer**	21 Monate (12 Monate Rekrutierungsdauer)
**Studientyp, Studiendesign**	• einarmige prospektive Längsschnittstudie<br>• multizentrische Interventionsstudie inklusive Fragebogen- und Funktionsdiagnostik vor und nach Abschluss der Intervention sowie 3 Monate Follow Up
**Methodische Vorgehensweise**	• lokale Rekrutierung der Studienteilnehmer an den Standorten der teilnehmenden Kliniken/Praxen<br>• bei Erfüllung der Ein-/Ausschlusskriterien (Arzt) Studienaufklärung und Einholung der Einwilligung (Arzt/Therapeut/Dokumentar)<br>• Eingangsdiagnostik: Allgemeiner Fragebogen zu Erfassung personenspezifischer Daten, Erfassung der QoL mittels Fragebögen EORTC QLQ-30 und EORTC H&N35, Erfassung der körperlichen Aktivität mittels Fragebogen GSLTPAQ<br>• Erfassung der krankheitsspezifischen Daten (Arzt)

	• Leistungsdiagnostik pre: Erfassung des momentanen IST-Zustandes mittels Funktionsdiagnostik<br>• Datenweiterleitung an Studienzentrale der UMR<br>• zentrale Trainingsplanung und Übungszusammenstellung unter Berücksichtigung der individuellen Leistungsfähigkeit der Patienten<br>• Übermittlung der individuellen Trainingspläne inkl. Übungsvideos, Übungshandbuch und Kleingeräte an lokale Therapeuten<br>• lokale Einweisung der Patienten ins Trainingsprogramm und Ausgabe der Materialien, des Übungshandbuches, der individuellen Trainingsvideos sowie des Trainingstagebuchs<br>• Durchführung des Heimtrainingsprogrammes über 12 Wochen inkl. Dokumentation des Trainings (Erfassung des Trainingsumfanges) und wöchentlichen Anruf des lokalen Therapeuten (Erfragung Status quo, Motivation, Erfassung Adverse Events)<br>• Nach Interventionsabschluss: Ausgangsfragebogen zur Erfassung der Motivation sowie Fragebogenerhebung (EORTC QLQ-30, EORTC H&N35 und GSLTPAQ) und Leistungsdiagnostik post analog der Diagnostik pre<br>• Follow Up Untersuchung nach weiteren 12 Wochen analog pre/post<br>• Erfassung der in die Studie eingeschlossenen Teilnehmer und der Anzahl derer, die das 12-wöchige Training vollständig absolviert haben<br>• Erfragung/Erfassung des Abbruchgrundes
**Datenbasis**	Trainingsumfang, Patientenbefragung und Funktionsdiagnostik
**Patienten**	**Einschlusskriterien:**<br>• Alter ≥ 18 Jahre<br>• Patienten mit Tumorerkrankung im Mund-, Kiefer-, Gesichts- und Halsbereich in der Nachsorge, mind. 4 Monate nach antineoplastischer Therapie (Radiatio, Chirurgie, Chemo- oder Immuntherapie) bzw. nach Abschluss der Anschlussheilbehandlung<br>• gehfähig ohne Gehhilfe<br>• beherrschen der deutschen Sprache in Wort und Schrift<br>• Patient muss körperlich und psychosozial in der Lage sein, an der Studie teilzunehmen (ärztliche Einschätzung)<br><br>**Ausschlusskriterien:**<br>Patienten, die mindestens eines der folgenden Kriterien erfüllen, werden nicht in diese Studie eingeschlossen:<br>• nicht-einwilligungsfähige Patienten<br>• nicht alle Einschlusskriterien erfüllt<br>• klinisch relevante Herzinsuffizienz (NYHA III und IV),<br>• frischer Myokardinfarkt (< 4 Wochen),<br>• instabile Angina pectoris,<br>• höhergradige Klappenvitien (in Anamnese),<br>• nicht kontrollierte Herzrhythmusstörungen,<br>• chronisch obstruktive Lungenerkrankung Krankheitsstadium III oder höher gemäß GOLD,<br>• paVK ab Stadium III nach Fontaine

	• Krankheiten, die die kognitive Leistungsfähigkeit ernsthaft beeinträchtigen könnten (z. B. Demenz, Schlaganfall, Wernicke-Korsakoff-Syndrom)<br>• < 24 Punkte Mini-Mental State Examination (MMSE)<br>• bekannte Alkoholabhängigkeit
**Stichprobengröße**	N = 60 (davon 25 Phase I)
**Datenanalyse**	Personenbezogene Daten: Geschlecht, Alter, Größe, Gewicht, Familienstand, Schulabschluss, Berufsstatus, Besuch Selbsthilfegruppe<br>Nikotin- und Alkoholkonsum<br>Krankheitsspezifische Daten: Diagnose inkl. Stadium, Jahr der Diagnose, medizinische Therapie(n)<br>Daten zur Sportvergangenheit<br>Daten zur Intervention: Advers Events, Motivation, Zufriedenheit mit Betreuung und Unterlagen, Trainingsumfang, eventueller Abbruchsgrund und -zeitpunkt<br>→Schätzung der Dropout Rate<br>→Deskriptive Statistik<br><br>Fragebögen (pre/post/follow up): Angaben zur QoL (EORTC QLQ-30, EORTC H&N35), körperliche Aktivität (GSLTPAQ), Erfassung der aktuellen Müdigkeit (POMS-F) am Tag der Leistungsdiagnostik<br>→Berechnung der mittleren individuellen Differenzen und Testung auf Null; t-Test für abhängige Stichproben/ Wilcoxon/ McNemar<br>→ Varianzanalyse mit Messwiederholung<br><br>Funktionsdiagnostik (pre/post/follow up):<br>Interzahnabstand bei maximaler Mundöffnung, Bewegungsumfang (ROM) der Schultergelenke und der Halswirbelsäule, Flexibilität des Rumpfes (stand and reach test), Short Physical Performance Battery (Gleichgewicht, Gehgeschwindigkeit, Beinkraft), 6-Minuten-Geh-Test (kardiovaskuläre und pulmonale Leistungsfähigkeit) inkl. BORG- und CR-10-Skala (Anstrengungsempfinden und Belastungsschmerz), optional Bioimpedanzanalyse (BIA)<br>→ Berechnung der mittleren individuellen Differenzen und Testung auf Null; t-Test für abhängige Stichproben/ Wilcoxon/ McNemar)<br>→ Verteilungsprüfung auf Gleichheit χ^2-Test; Risikobewertung bzw. Ermittlung von Assoziationsmaßen und Testung auf Null;<br>→ Varianzanalyse mit Messwiederholung<br>→ Korrelations- /Regressionsanalysen<br><br>Die Datenauswertung erfolgt in Zusammenarbeit mit dem IBIMA

III. Inhaltsverzeichnis

I. Unterschriften 3
II. Synopse 4
III. Inhaltsverzeichnis 8
IV. Abkürzungsverzeichnis 10
1. Einleitung 11
2. Ziele der Studie 11
2.1. Rationale 11
2.2. Eigene Vorarbeiten 11
2.3. Ziel der Studie 12
2.4. Primäres Ziel 12
2.5. Sekundäre Ziele 13
3. Organisationsstruktur 14
3.1. Sponsor 14
3.2. Beteiligte Einrichtungen 14
3.3. Finanzierung 14
4. Studiendurchführung 15
4.1. Allgemeines Studiendesign 15
4.2. Diskussion des Studiendesigns 17
4.3. Auswahl der Studienpopulation 17
4.3.1. Ein- und Ausschlusskriterien 17
4.4. Nachträglicher Ausschluss von Studienteilnehmern 18
4.5. Intervention 18
4.6. Dokumentation 19
4.6.2. Datenmanagement 20
4.6.3. Datenauswertung 20
4.6.4. Archivierung 21
5. Ethische und regulatorische Aspekte 22
5.1. Unabhängige Ethikkommissionen 22
5.2. Ethische Durchführung der klinischen Prüfung 22
5.2.1. Berücksichtigte gesetzliche Bestimmungen und Leitlinien 22
5.3. Aufklärung und Einwilligung der Prüfungsteilnehmer 22
5.4. Probandenversicherung 23
5.5. Datenschutz 23
5.5.1. Schutz persönlicher Daten 23
5.5.2. Speicherung und Weitergabe pseudonymisierter Daten 23

6. Statistische Methoden und Ermittlung der Fallzahl 24
6.1. Statistischer und analytischer Plan 24
6.1.1. Studienpopulationen 24
6.1.2. Beschreibung des Patientenkollektivs 24
6.1.3. Primäre Zielvariable 24
6.1.4. Sekundäre Zielvariablen 24
6.1.5. Subgruppenanalysen 25
6.2. Ermittlung der Fallzahl 25
7. Sicherheit 26
7.1.1. Unerwünschtes Ereignis (AE) 26
8. Verwendung der Daten und Publikation 28
8.1. Berichte 28
8.1.1. Zwischenberichte 28
8.1.2. Abschlussbericht 28
8.2. Publikation 28
9. Änderungen des Prüfplans 28
10. Anhang 29
11. Literatur 30

IV. Abkürzungsverzeichnis

**Abkürzung**	**Bedeutung**
AE	Adverse Event
BIA	Bioimpedanzanalyse
BORG-Skala	Skala zur Erfassung des subjektiven Belastungsempfindens
CIPN	Chemotherapie-Induzierte Periphere Neuropathie
EORTC QLQ-30	European Organization for Research and Treatment of Cancer Quality of Life Questionnaire (Fragebogen zur Erfassung der Lebensqualität)
GSLTPAQ	Godin Leisure-Time Physical Activity Questionnaire
HNSCC	Head Neck Sarcoma Cell Cancer
IBIMA	Institut für Biostatistik und Informatik in Medizin und Alternsforschung der UMR
MMSE	Mini-Mental State Examination
OSHO	Ostdeutsche Studiengruppe Hämatologie und Onkologie e.V.
POMS-F	Profile of Moods States Fatigue (Fragebogen zur Erfassung der aktuellen Stimmung)
PRO	Patient Reported Outcome
QoL	Lebensqualität
ROM	Range of Motion
SAE	Serious Adverse Event
SPPB	Short Physical Performance Battery
TTB	Trainingstagebuch
UMR	Universitätsmedizin Rostock
VAS	Visuelle Analog-Skala

1. Einleitung

Weltweit machen Kopf-Hals-Tumore (HNSCC) mehr als 685.000 Fälle mit 375.000 Todesfällen pro Jahr aus [1]. Die Prognose des Robert-Koch-Instituts zeigt für 2020 zirka 20.000 Neuerkrankungen in Deutschland [2]. Die therapeutischen Ansätze sind durch die enge Zusammenarbeit von Chirurgen, Strahlentherapeuten, Onkologen und anderen Disziplinen interdisziplinär. Aufgrund der Lage des Tumors und der intensiven lokalen Therapieverfahren haben diese Patienten oft eine hohe chronische Symptombelastung. Viele Patienten leiden unter Dysphonie, Dysphagie, Lymphödem, Gewichtsverlust, Muskelschwund, Müdigkeit, Chemotherapie-induzierter peripherer Neuropathie (CIPN), Schmerzen und Bewegungseinschränkungen, welche die Lebensqualität (QoL) beeinträchtigen [3–6]. Daher wird für HNSCC-Patienten eine interdisziplinäre Rehabilitation einschließlich der Behandlung durch Ärzte, Psychologen, Sprach- und Bewegungstherapeuten, Ernährungswissenschaftler und Ergotherapeuten angestrebt [7]. Im Allgemeinen zielt die Rehabilitation auf die Symptomlinderung und den Ausgleich von Funktionsstörungen [3]. Es gibt starke Belege für die Vorteile von Bewegung/körperlicher Aktivität in Bezug auf die Körperzusammensetzung, die muskuläre Fitness, die Schmerzen, die Flexibilität und die QoL für HNSCC-Patienten [7–14]. Somit ist die Bewegungstherapie ein Schlüsselfaktor für das Wohlbefinden/Outcome der Patienten.

2. Ziele der Studie

2.1. Rationale

Viele bisherige Interventionsstudien mit HNSCC-Patienten konzentrierten sich auf ein standardisiertes Training in der Nachsorge. Dabei wurden häufig große Trainingsgeräte verwendet, die kostspielig sind oder spezielle Trainingseinrichtungen erfordern. Darüber hinaus konzentrierten sich die meisten Studien auf eine einzige Trainingsmethode (z. B. progressives Krafttraining), bei der nur ein Symptom im Mittelpunkt stand [8,10–13]. Da die meisten Patienten jedoch unter verschiedenen Beeinträchtigungen leiden (Bewegungseinschränkung, Schmerzen, Muskelschwund, CIPN, Müdigkeit), besteht Bedarf an umfassenderen ganzheitlichen Trainingsprogrammen. Zudem zeigen Befragungen, dass viele HNSCC-Patienten ein Training zu Hause, allein und mit mittlerer Intensität bevorzugen [15,16]. Folglich benötigen HNSCC-Patienten ein Trainingsprogramm, das zu Hause durchführbar ist, ohne große/teure Trainingsgeräte und unter Berücksichtigung ihrer körperlichen sowie psychischen Beeinträchtigungen. Heimtrainingsprogramme bieten zudem den Vorteil, dass theoretisch alle HNSCC-Patienten davon profitieren können. Die Trainingsprogramme können individuell auf die jeweiligen Bedürfnisse des Patienten abgestimmt werden und räumlich/zeitlich flexibel durchgeführt werden. Zudem sind diese zeitlich nicht begrenzt wie übliche Rehabilitationsmaßnahmen.

2.2. Eigene Vorarbeiten

Um ein großgerätefreies Heimtrainingsprogramm für HNSCC zu entwickeln und zu evaluieren wurde 2018/19 an der UMR (ZIM III) eine erste Pilotstudie (A 2018-0153) durchgeführt. Im ersten Schritt wurde ein Trainingsprogramm mit Übungen, die für ein eigenständiges Training zu Hause bei HNSCC-Patienten geeignet sind, zusammengestellt. Im Anschluss wurde dieses Trainingsprogramm mittels einer 12-wöchigen Trainingsintervention hinsichtlich der Compliance der Patienten, der Effekte auf die physische Funktionalität und QoL evaluiert. In dieser Studie absolvierten die Patienten das Training unter Anleitung in Kleingruppen. Insgesamt umfasste die Intervention 24 Trainingseinheiten á 50 min. Im Durchschnitt setzte sich jede Einheit

aus 15 min Erwärmung/Mobilisation (Funktionsgymnastik), 10 min Koordinations-, 15 min Kräftigungs- und 10 min Dehnungs-/Entspannungsübungen zusammen. Das Training wurde mit niedriger bis mittlere Intensität durchgeführt.

Insgesamt wurden 12 Patienten (6 männlich, 6 weiblich, 68 ± 9 Jahre) in die Studie eingeschlossen. Zehn (83%) schlossen diese erfolgreich ab. Die Anwesenheitsrate der Teilnehmer lag bei 83% (20 ± 3 von 24 Einheiten). Bei den Funktionstests zeigten sich diverse signifikante Verbesserungen im Bewegungsausmaß (ROM) der Schultergelenke und der Halswirbelsäule. Nach Abschluss der Intervention erreichten alle Teilnehmer in der Short Physical Performance Battery (SPPB, Test umfasst Gleichgewicht, Beinkraft und Gehgeschwindigkeit) die volle Punktzahl. Beim 6-Minuten-Gehtest legten die Patienten nach der Intervention eine um 43 m (+8,3%) weitere Strecke zurück, wobei das subjektive Belastungsempfinden signifikant geringer war. Um Veränderung in der QoL zu evaluieren, wurden die Fragebögen EORTC-QLQ-30 und EORTC H&N35 vor und nach Intervention von den Teilnehmern ausgefüllt. Die Analyse zeigte eine tendenzielle Verbesserung in der globalen QoL ($p = 0,059$). Wobei die Effektgröße einem mittleren Effekt ($d = 0,626$) entspricht. Positive Veränderungen zeigten sich in drei der fünf Funktionsskalen des EORTC-QLQ-30: körperliche Funktion (+6,7 Punkte, $p = 0,008$, $d = 1,077$), kognitive Funktion (+48,3 Punkte, $p = 0,015$, $d = 0,829$) und soziale Funktion (+23,4 Punkte, $p = 0,031$, $d = 0,604$). Es gab keine statistisch signifikanten Veränderungen in den drei Symptomskalen und sechs Single-Items. In den Symptomskalen des H&N35 zeigte nur die Sexualität eine signifikante Verbesserung (-16,5 Punkte, $p = 0,031$, $d = 0,928$). Die Skala der "Sozialkontakte" zeigt eine Tendenz zur Verbesserung (-8,6 Punkte, $p = 0,68$, $d = -0,542$) [17,18].

Die gute Compliance der Teilnehmer, die positiven Effekte hinsichtlich physischer Funktionalität und QoL bieten eine hervorragende Grundlage für die Überführung in ein Heimtrainingsprogramm (Home-based Setting).

Im Rahmen der Pilotstudie wurde ein „Übungshandbuch für Patienten mit Mund-, Kiefer-, Gesichts- und Halstumoren“ verfasst (ISBN: 978-1099096082). Es umfasst insgesamt 90 Mobilisations-, Koordinations-, Kräftigungs- und Dehnungsübungen für das Training zu Hause. Analog zum Buch wurden Videos zu den Übungen erstellt. Sowohl Buch wie auch Videos werden den studienteilnehmenden HNSCC-Patienten für das eigenständige Üben zu Hause zur Verfügung gestellt.

2.3. Ziel der Studie

Die vorliegende Studie setzt sich zum Ziel, den Ansatz des individuellen Heimtrainings bei HNSCC-Patienten in einer multizentrischen Studie bezüglich seiner Machbarkeit und seiner Effekte auf verschiedenen Grade der QoL zu evaluieren.

Um alle Zielstellungen der Studie adäquat bedienen zu können, werden folgende primäre und sekundäre Outcomes definiert:

2.4. Primäres Ziel

Phase I (Machbarkeit)
Bestimmung des prozentualen Anteils der Patienten, die die Trainingsintervention im home based setting über 12 Wochen durchführen.

Phase II (Lebensqualität)

Veränderung des globalen QoL-Scores (EORTC-QLQ-30) nach einer 12-wöchigen Trainingsintervention im Home-based Setting

2.5. Sekundäre Ziele

- Gründe für einen vorzeitigen Abbruch der 12-wöchigen Trainingsintervention
- Häufigkeit und Dauer des Trainings im Home-based Setting im Durchschnitt pro Woche (Trainingsumfang)
- Mittelfristige Veränderung der QoL nach einer 12-wöchigen Trainingsintervention im Home-based Setting
- Kurzfristige Veränderung der Beweglichkeit nach einer 12-wöchigen Trainingsintervention im Home-based Setting
- Mittelfristige Veränderung der Beweglichkeit nach einer 12-wöchigen Trainingsintervention im Home-based Setting
- Kurzfristige Veränderung der Alltagsaktivität nach einer 12-wöchigen Trainingsintervention im Home-based Setting
- Mittelfristige Veränderung der Alltagsaktivität nach einer 12-wöchigen Trainingsintervention im Home-based Setting
- Kurzfristige Veränderung der Gleichgewichtsfähigkeit /Gangsicherheit nach einer 12-wöchigen Trainingsintervention im Home-based Setting
- Mittelfristige Veränderung der Gleichgewichtsfähigkeit /Gangsicherheit nach einer 12-wöchigen Trainingsintervention im Home-based Setting
- Kurzfristige Veränderung der aeroben Ausdauerleistungsfähigkeit nach einer 12-wöchigen Trainingsintervention im Home-based Setting
- Mittelfristige Veränderung der aeroben Ausdauerleistungsfähigkeit nach einer 12-wöchigen Trainingsintervention im Home-based Setting
- Kurzfristige Veränderungen in der Körperzusammensetzung nach einer 12-wöchigen Trainingsintervention im Home-based Setting
- Mittelfristige Veränderungen in der Körperzusammensetzung nach einer 12-wöchigen Trainingsintervention im Home-based Setting
- Zusammenhänge zwischen dem Trainingsumfang während der 12-wöchigen Trainingsintervention und den Effekten hinsichtlich QoL
- Zusammenhänge zwischen dem Trainingsumfang während der 12-wöchigen Trainingsintervention und den Effekten hinsichtlich der Funktionalität
- Zusammenhänge zwischen dem Trainingsumfang während der 12-wöchigen Trainingsintervention und den Effekten hinsichtlich der Körperzusammensetzung
- Zusammenhänge zwischen Alltagsaktivität und QoL
- Zusammenhänge zwischen Alltagsaktivität und Funktionalität
- Zusammenhänge zwischen Alltagsaktivität und Körperzusammensetzung
- Unterschiede bei der Wahrnehmung der subjektiven Effekte des Heimtrainings in Abhängigkeit vom Geschlecht

3. Organisationsstruktur

3.1. Sponsor

Prof. Dr. med. Christian Junghanß
xxx

3.2. Beteiligte Einrichtungen

- Klinik III (Hämatologie, Onkologie, Palliativmedizin) (Direktor: Prof. Dr. med. Christian Junghanß)
- Klinik und Poliklinik für Mund-, Kiefer- und Plastische Gesichtschirurgie, UMR (Ansprechpartner: Dr. med. Jan Liese)
- Klinik und Poliklinik für Hals-Nasen-Ohrenheilkunde, Kopf- und Halschirurgie, UMR (Ansprechpartner: Dr. Daniel Strüder)
- Studienzentrale der Klinik III, Hämatologie, Onkologie und Palliativmedizin, UMR (Leiterin: Dr. med. Brigitte Kragl)
- Institut für Biostatistik und Informatik in Medizin und Alternsforschung (IBIMA) der UMR (Direktor Prof. Dr. G. Füllen, Ansprechpartnerin: PD Dr. Änne Glass)
- Kooperationspartner: Zentren/Kliniken/Praxen der OSHO e. V.
  - Helios Kliniken Schwerin
  - Universitätsmedizin Halle (Saale) / Krukenberg-Krebszentrum Halle
  - Ernst von Bergmann Klinikum Potsdam
  - Städtisches Klinikum Dresden
  - Universitätsklinikum Leipzig
  - Ggf. weitere Zentren der OSHO e. V.

3.3. Finanzierung

Die Finanzierung der Studie erfolgt aus Zuwendungen der OSHO e.V. (Anschubfinanzierung), zum Teil aus Drittmitteln des Projektes „Bewegungstherapie während und nach der Krebstherapie – ein Projekt zur Stärkung der Lebensqualität“ gefördert durch die Krebsgesellschaft M-V e. V. sowie aus Drittmitteln der Klinik III (Hämatologie, Onkologie, Palliativmedizin).

4. Studiendurchführung

4.1. Allgemeines Studiendesign

Bei der Studie handelt es sich um eine multizentrische Interventionsstudie inklusive Fragebogen- und Funktionalitätserhebung vor und nach Abschluss der Intervention sowie drei Monate Follow Up (prospektive Längsschnittstudie). Der Studienablauf ist schematisch in Abb. 1 dargestellt.

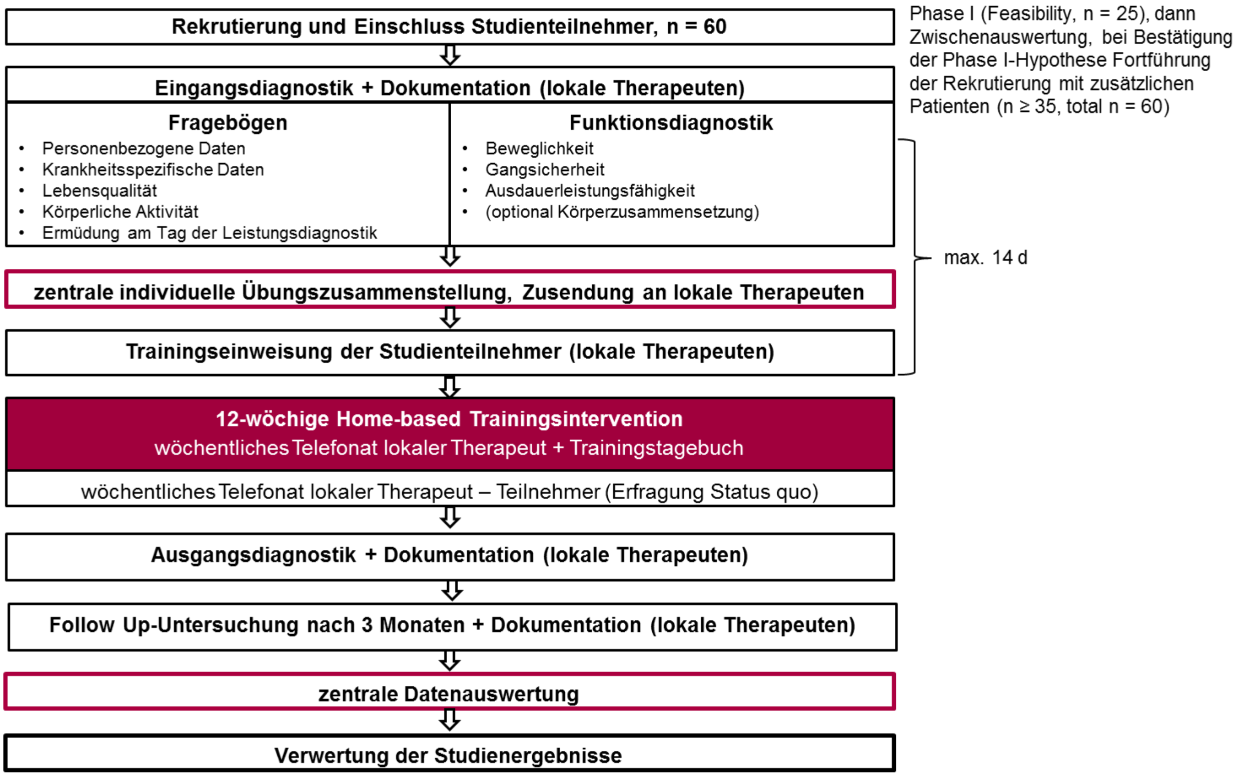

Abb. 1 Flussdiagramm zum Studienablauf

Vor Rekrutierung der Studienteilnehmer an den einzelnen Standorten werden alle Therapeuten, die für die Eingangs- und Ausgangsdiagnostik sowie für die Einweisung des Patienten in das Trainingsprogramm verantwortlich sind von einem Projektmitarbeiter am Standort UMR in die Fragebögen, Messungen, Protokolle und Übungsmaterialien eingewiesen. Die Therapeuten erhalten eine Übersicht über alle Aufgaben und Dokumente, die zu den jeweiligen Untersuchungszeitpunkten ausgeteilt bzw. ausgefüllt werden sollen. Während des gesamten Studienzeitraums wird bei Bedarf (z. B. bei Fragen) der Kontakt zwischen lokalen Therapeuten (Ärzten) und der Studienleitung der UMR (per Mail, telefonisch, per Videokonferenz) hergestellt.

Die Rekrutierung der Studienteilnehmer erfolgt lokal an den Standorten der teilnehmenden Zentren/Kliniken/Praxen. Bei Erfüllung der Ein-/Ausschlusskriterien erfolgt die Patientenaufklärung durch einen Arzt / Therapeuten oder Dokumentar. Willigt der Patient in die Studie ein, schließt sich die Eingangsdiagnostik (pre-Test) an. Diese besteht aus zwei Teilen:

- Teil 1 „Patient Reported Outcomes“ (PROs) und
- Teil 2 Funktionsdiagnostik.

Zu den PROs zählen:
a) Erfassung krankheitsspezifischer Daten hinsichtlich der Tumorerkrankung (behandelnder/einschließender Arzt)
b) Erfassung personenspezifischer Daten (z. B. Nebenwirkungen)
c) Erfassung der QoL mittels der Fragebögen EORTC QLQ-30 und EORTC H&N35
d) Erfassung der körperlichen Aktivität mittels Fragebogen GSLTPAQ.

Die Funktionsdiagnostik zur Erfassung des momentanen IST-Zustandes beinhaltet:
a) Messung des Interzahnabstand bei maximaler Mundöffnung
b) Messung des ROM der Schultergelenke und der Halswirbelsäule
c) Messung der Flexibilität des Rumpfes mittels „stand and reach test"
d) Durchführung der „Short Physical Performance Battery" (Gleichgewicht, Gehgeschwindigkeit, Beinkraft),
e) Absolvieren des 6-Minuten-Geh-Tests inkl. BORG- und CR-10-Skala (Anstrengungsempfinden und Belastungsschmerz) sowie
f) optional die Durchführung einer Bioimpedanzanalyse (BIA).

Die erhobenen Daten werden im Anschluss pseudonymisiert an die Studienzentrale der UMR weitergeleitet. Hier erfolgt zentral die individuelle Trainingsplanung und Übungszusammenstellung unter Berücksichtigung der aktuellen Leistungsfähigkeit/Einschränkungen der Patienten. Nach Übermittlung des Trainingsplans inkl. Übungszusammenstellung (Video) an den lokalen Therapeuten, erfolgt lokal die Einweisung des Patienten in das Trainingsprogramm und die Materialien. Dem Patienten werden Hinweise zum Heimtraining (Häufigkeit, Dauer, Intensität, Informationen dazu, wann kein Training absolviert werden sollte) gegeben, er erhält ein Übungshandbuch, ein individuelles Trainingsvideo und die benötigten Materialien (Gummiband, Gymnastikball). Zusätzlich erklärt ihm der Therapeut, wie das Trainingstagebuch (TTB) auszufüllen ist.
Es wird angestrebt, dass zwischen Eingangsdiagnostik und Trainingseinweisung maximal 14 Tage liegen.
Nach der Einweisung erfolgt die Durchführung des Trainings im Home-based Setting über einen Zeitraum von 12 Wochen. Der Patient dokumentiert täglich die Trainingsdauer im TTB. Einmal wöchentlich erfolgt ein Anruf vom lokalen Therapeuten, um den Status quo (Wohlbefinden, Trainingshäufigkeit und -dauer, Auftreten unerwünschter Ereignisse beim Training (AEs/SAEs), Motivation) zu erfragen. Der Therapeut hält die Angaben in einem Telefonprotokoll fest. Gleichzeitig soll der Anruf der Motivation dienen. Sollten Patienten die Intervention vorzeitig abbrechen, wird der Grund des Abbruchs erfragt.
Im Anschluss an die 12-wöchige Trainingsintervention erfolgt die Ausgangsdiagnostik (post-Test). Diese erfolgt analog zur Eingangsdiagnostik. Einzig der allgemeine Fragebogen wird gegen einen Ausgangsfragebogen ersetzt. In diesem wird die Zufriedenheit mit der Einweisung und den Materialen sowie die Motivation zur Beibehaltung des Trainings erfasst. Am Tag der Ausgangsdiagnostik übergibt der Patient sein TTB dem Therapeuten. Alle Daten inkl. TTB werden im Anschluss pseudonymisiert an die Studienzentrale der UMR weitergeleitet.
Drei Monate nach der Ausgangsdiagnostik erhält der Patient einen Termin für die Follow Up Untersuchung. Die Fragebögen werden dem Patienten zirka eine Woche vor dem Termin zugesandt. PROs und Leistungsdiagnostik erfolgen analog den pre- und post-Tests. Nach der Untersuchung werden alle Daten pseudonymisiert an die Studienzentrale der UMR weitergeleitet, wo die zentrale Datenerfassung und -auswertung erfolgt.

Patientenbezogen:

1. Aufklärung und Einwilligung
2. Eingangsdiagnostik (pre-Test)
3. Intervention: 12 Wochen im Home-based Setting nach Einweisung
4. Ausgangsdiagnostik (post-Test)
5. Follow Up Untersuchung nach 3 Monaten

Studienbezogen:

Rekrutierungsdauer: 12 Monate
Gesamtzeitdauer Intervention inkl. Follow Up (Datenerhebung): 18 Monate
Gesamtdauer inkl. Abschlussbericht: 21 Monate

Einschluss erster Patient	01.01.2021
Einschluss letzter Patient	31.12.2021
Prüfungsende des letzten Patienten	30.06.2022
Integrierter Abschlussbericht	30.09.2022

Tab. 1 Zeitplan der Studie

4.2. Diskussion des Studiendesigns

Primär wird der kurz- und mittelfristige Effekt des Heimtrainings auf die QoL der Patienten betrachtet. Das heißt, die intraindividuelle Veränderung der QoL zwischen den Zeitpunkten pre (vor der Intervention), post (nach der Intervention) und Follow Up steht im Fokus. Eine Kontrollgruppe ist somit nicht zwingend erforderlich.

4.3. Auswahl der Studienpopulation

Es werden nur Patienten eingeschlossen, die eine endstellig kodierte Diagnose gemäß ICD: C00 - C14, C30 - C32 Bösartige Neubildungen der Lippe, der Mundhöhle, des Rachens, der oberen Atemwege („Kopf-Hals-Tumore") erhalten haben. Es werden keine besonders schutzbedürftigen Personen eingeschlossen.

Um den Trainingsumfang und die Effekte des Heimtraining auch in Abhängigkeit von Geschlecht beurteilen zu können, sollen nach Möglichkeit Männer und Frauen im gleichen Verhältnis in die Studie eingeschlossen werden. Allerdings muss berücksichtigt werden, dass dreimal so viele Männer wie Frauen an Kopf-Hals-Tumoren erkranken [2], die Teilnahme an der Studie freiwillig ist und u. a. von der Motivation der Patienten aber auch vom Gesundheitszustand (Ein- und Ausschlusskriterien) abhängt.

4.3.1. Ein- und Ausschlusskriterien

Die Rekrutierung von Studienteilnehmern ist an bestimmte Ein- und Ausschlusskriterien gebunden, die nachfolgend aufgelistet werden:

Einschlusskriterien:

- Alter ≥ 18 Jahre
- Patienten mit Tumorerkrankung im Mund-, Kiefer-, Gesichts- und Halsbereich in der Nachsorge, mind. 4 Monate nach antineoplastischer Therapie (Radiatio, Chirurgie, Chemo- oder Immuntherapie) bzw. nach Abschluss der Anschlussheilbehandlung
- gehfähig ohne Gehhilfe

- beherrschen der deutschen Sprache in Wort und Schrift
- Patient muss körperlich und psychosozial in der Lage sein, an der Studie teilzunehmen (ärztliche Einschätzung)

Ausschlusskriterien:
Patienten, die mindestens eines der folgenden Kriterien erfüllen, werden nicht in diese Studie eingeschlossen:

- nicht-einwilligungsfähige Patienten
- nicht alle Einschlusskriterien erfüllt
- klinisch relevante Herzinsuffizienz (NYHA III und IV),
- frischer Myokardinfarkt (< 4 Wochen),
- instabile Angina pectoris,
- höhergradige Klappenvitien (in Anamnese),
- nicht kontrollierte Herzrhythmusstörungen,
- chronisch obstruktive Lungenerkrankung Krankheitsstadium III oder höher gemäß GOLD,
- paVK ab Stadium III nach Fontaine
- Krankheiten, die die kognitive Leistungsfähigkeit ernsthaft beeinträchtigen könnten (z. B. Demenz, Schlaganfall, Wernicke-Korsakoff-Syndrom)
- < 24 Punkte Mini-Mental State Examination (MMSE)
- bekannte Alkoholabhängigkeit

4.4. Nachträglicher Ausschluss von Studienteilnehmern

Ein nachträglicher Studienausschluss erfolgt, sofern ein Patient seine Einwilligung widerruft.

4.5. Intervention

Die Trainingsintervention erfolgt im Home-based Setting über einen Zeitraum von 12 Wochen. Den Patienten wird empfohlen die individuell zusammengestellten Übungen an mindestens drei Tagen pro Woche zu trainieren. Die Trainingsdauer pro Tag sollte 15 - 30 min betragen. Zudem wird dem Patienten empfohlen (zusätzlich zum Training der Übungen) 2- bis 3-mal pro Woche für zirka 30 min ein Ausdauertraining zu absolvieren, z. B. in Form eines Spazierganges, (Nordic-)Walking, Rad fahren, Schwimmen, Tanzen oder ähnlichem. Um eine Überlastung durch das Training zu vermeiden, wird zur Steuerung des Trainings die BORG-Skala (siehe Anhang, Anlage 6) empfohlen. Die Borg-Skala von 6 („sehr, sehr leicht") bis 20 („sehr, sehr anstrengend") misst den individuell empfundenen Grad der Anstrengung beim Kraft- und Ausdauertraining. Die optimale Anstrengung liegt zwischen den Werten 11 und 15.

Die individuelle Übungszusammenstellung erfolgt durch Sportwissenschaftler und/oder Physiotherapeuten auf Grundlage der Ergebnisse der Eingangsdiagnostik. Entsprechend der Leistungsfähigkeit/Einschränkungen des Patienten kommen verschiedene Mobilisations-, Koordinations-, Kräftigung- und Dehnungsübungen zum Einsatz, die vermeintlich positive Effekte auf die körperliche Leistungsfähigkeit, Funktionalität und QoL haben. Alle zu empfehlenden Übungen finden sich im „Übungshandbuch für Patienten mit Mund-, Kiefer-, Gesichts- und Halstumoren", welches die Studienteilnehmer erhalten. Zusätzlich werden die ausgewählten Übungen als Video auf einen Datenträger (DVD/Stick) gespeichert, den der Patient ebenfalls erhält. Je nach Rückmeldung der Teilnehmer, besteht die Möglichkeit nach den ersten Trainings weitere Übungen zu empfehlen und als Videodateien zur Verfügung stellen (z. B. anspruchsvol-

lere oder schwerere Übungen bei Verbesserung der Leistungsfähigkeit). Materialen wie Gummibänder und Gymnastikbälle, die für die Übungen benötigt werden, werden dem Patienten kostenlos bereitgestellt.

Der Patient protokolliert sein durchgeführtes Training in einem Trainingstagebuch. Einmal wöchentlich erfragt ein lokaler Therapeut telefonisch den Status quo und protokolliert diesen.

4.6. Dokumentation

Bei der Eingangs-, Ausgangs- und Follow Up-Untersuchung kommen folgende Fragebögen zur Anwendung:

- Fragebogen zur Erfassung krankheitsspezifischer Daten (Anlage 1)
- Allgemeiner Fragebogen zur Erfassung personenbezogener Daten (nur Eingangsuntersuchung) (Anlage 2)
- EORTC-QLQ-30 und EORTC Modul H&N 35: Erfassung der QoL (Anlage 3)
- Godin Leisure-Time Physical Activity Questionnaire (GSLTPAQ): Erfassung der körperlichen Aktivität (Anlage 4)
- Abschlussfragebogen: Erfassung der Zufriedenheit mit Trainingsprogramm und bereitgestellten Medien/Kleingeräten und der Betreuung, aktuelle Motivation der Studienteilnehmer hinsichtlich Fortführung des Trainingsprogrammes (nur Abschlussuntersuchung) (Anlage 5)

Zur objektiven Beurteilung der physischen Leistungsfähigkeit, der Funktionalität und der Körperzusammensetzung kommen verschiedene Untersuchungen zum Einsatz. Zu diesen zählen:

- Profile of moods states fatigue-Scale (POMS-F): Erfassung der aktuellen Stimmung
- Messung der Schneidekantendifferenz (SKD): Erfassung der Kiefergelenksbeweglichkeit
- Messung Range of motion (ROM) der Halswirbelsäule und der Schultergelenke mittels manueller Goniometer (objektive Messungen) und Beurteilung des Bewegungsausmaßes/der Bewegungseinschränkungen aus Sicht des Patienten und des Therapeuten (subjektive Angaben)
- Stand and reach Test: Erfassung der Beweglichkeit der rückwärtigen Muskulatur
- SPPB: Testbatterie zur Beurteilung der Gleichgewichtsfähigkeit, Beinkraft und Gehgeschwindigkeit
- 6-Minuten-Gehtetst inkl. BORG- und CR-10-Skala: Beurteilung der aeroben Ausdauerleistungsfähigkeit, Erfassung des subjektiven Belastungsempfindens und des subjektiven Belastungsschmerzes
- Optional BIA: Erfassung der Körperzusammensetzung

Die Gesamtdauer der Leistungsdiagnostik beträgt zirka 30 min. Die Ergebnisse werden in einem Protokoll dokumentiert (Anlage 6).

Die aufgelisteten Untersuchungen werden sowohl vor (pre-Test) und nach der Home-based Intervention (post-Test) sowie drei Monate Follow Up durchgeführt.

Bei allen Patienten wird der Trainingsumfang in einem TTB (Anlage 7) dokumentiert. Im Falle eines Drop-outs wird der Grund des Abbruchs erfasst.

Die einmal wöchentlich durchgeführten Telefonate zum Status quo (Trainingsumfang, Motivation) werden schriftlich protokolliert (Anlage 8).

4.6.1. Datenerhebung

Im Rahmen dieser Studie werden sowohl personenbezogene wie auch krankheitsspezifischen Daten erhoben. Informationen zur QoL werden mittels Fragebögen erfasst. Hinzu kommen Daten der Leistungsdiagnostik und Angaben zur Intervention. Im Folgenden wird aufgeschlüsselt, welche Daten im Einzelnen erhoben werden:

Personenbezogene Daten: Geschlecht, Alter, Größe, Gewicht, Familienstand, Schulabschluss, Berufsstatus, Besuch Selbsthilfegruppe
Nikotin- und Alkoholkonsum
Krankheitsspezifische Daten: Diagnose inkl. Stadium, Jahr der Diagnose, medizinische Therapie(n)
Daten zur Sportvergangenheit
Daten zur Intervention: Motivation, Zufriedenheit mit Trainingsunterlagen und Betreuung, Trainingsumfang, eventueller Abbruchsgrund und -zeitpunkt

Fragebögen (pre/post/follow up): Angaben zur QoL (EORTC QLQ-30, EORTC H&N35), körperliche Aktivität (GSLTPAQ), Erfassung der aktuellen Müdigkeit (POMS-F) am Tag der Leistungsdiagnostik

Funktionsdiagnostik (pre/post/follow up):
Schneidekantendifferenz bei maximaler Mundöffnung [cm], Bewegungsumfang (ROM) der Schultergelenke und der Halswirbelsäule [°], Flexibilität des Rumpfes (stand and reach test) [cm], SPPB (Gleichgewicht, Gehgeschwindigkeit, Beinkraft) [Score 0 – 12], 6-Minuten-Geh-Test [m] (kardiovaskuläre und pulmonale Leistungsfähigkeit) inkl. BORG- und CR-10-Skala (Anstrengungsempfinden und Belastungsschmerz), optional BIA [fettfreie Masse bzw. Skelettmuskelmasse, Körperfettanteil bzw. Fettmasse].
Die Datenauswertung erfolgt in Zusammenarbeit mit dem IBIMA.

4.6.2. Datenmanagement

Die Daten werden in den teilnehmenden Zentren/Kliniken/Praxen erhoben und pseudonymisiert online über eine verschlüsselte Verbindung an die Studienzentrale der UMR versendet. Dabei werden keine personenbezogenen Daten (z. B. Name, Geburtsdatum, Anschrift, Telefonnummer etc.) versendet. Folglich ist auch bei versehentlich falschem Empfänger keine Zuordnung zu der Person möglich.

4.6.3. Datenauswertung

Bei dieser Studie handelt es sich um eine prospektive Längsschnittstudie. Für die Datenauswertung kommen die Schätzung der Drop-out Rate, deskriptive Statistiken, Zusammenhangsprüfungen (Korrelations-, Regressionsanalyse), mehrfaktorielle Verfahren zur Unterschiedsprüfungen (z. B. Varianzanalyse mit Messwiederholung) zum Einsatz. Die Datenauswertung wird in Zusammenarbeit mit dem IBIMA der UMR erfolgen.

Die Auswertung hinsichtlich Phase I (Machbarkeit) erfolgt nach 25 in die Studie eingeschlossenen Patienten. Zusätzlich werden ad hoc Analysen durchgeführt. Bei schneller Rekrutierung wird diese bei 60 in die Studie eingeschlossenen Patienten pausiert und je nach Analyseergebnissen ggfs. fortgeführt.

4.6.4. Archivierung

Die Patientenidentifikationslisten sowie alle Dokumentationsbögen, Einverständniserklärungen und weitere wichtige Prüfungsunterlagen werden gemäß §13 Abs. 10 GCP-V mindestens 15 Jahre an den jeweiligen Prüfzentren aufbewahrt.

5. Ethische und regulatorische Aspekte

5.1. Unabhängige Ethikkommissionen

Für die Studie wird eine zustimmende Bewertung der zuständigen Ethikkommission eingeholt. Erst dann wird die Studie begonnen. In jedem weiteren Prüfzentrum wird die Studie erst durchgeführt, nachdem die zuständige beteiligte Ethikkommission die Eignung der Prüfstelle und die Qualifikation der Prüfer festgestellt hat.

5.2. Ethische Durchführung der klinischen Prüfung

Der vorliegende Prüfplan sowie ggf. nachfolgende Änderungen des Prüfplans wurden bzw. werden in Übereinstimmung mit der Deklaration von Helsinki in der Fassung vom Oktober 1996 (48th General Assembly of the World Medical Association, Somerset West, Republic of South Africa) verfasst.

5.2.1. Berücksichtigte gesetzliche Bestimmungen und Leitlinien

Die vorliegende Studie wird in Übereinstimmung mit den veröffentlichten Grundsätzen der Good Clinical Practice (ICH-GCP)-Leitlinie und den zutreffenden gesetzlichen Bestimmungen (insbesondere der GCP-Verordnung) durchgeführt. Diese Grundsätze betreffen unter anderem Ethikkommissions-Vorgänge, Patientenaufklärung und Einwilligungserklärung, Befolgen des Protokolls, administrative Dokumente, Datenerhebung sowie Aufbewahrung von Unterlagen. Zum Schutz der personenbezogenen Daten wird die vorliegende Studie in Übereinstimmung mit der DSGVO durchgeführt.

5.3. Aufklärung und Einwilligung der Prüfungsteilnehmer

Ein Patient kann nur in die Studie aufgenommen werden, wenn er die Einwilligung hierzu erteilt hat, nachdem er durch einen Arzt, Therapeuten bzw. Dokumentar mündlich und schriftlich über Wesen, Bedeutung und Tragweite der Studie in angemessener und verständlicher Weise aufgeklärt worden ist. Er muss mit der Einwilligung zugleich erklärt haben, dass er mit der im Rahmen der Studie erfolgenden Aufzeichnung von Daten und ihrer Überprüfung die zuständige Überwachungs- oder Bundesbehörde einverstanden ist. Es muss ihm klar sein, dass er seine Einwilligung jederzeit und ohne Angabe von Gründen zurückziehen kann, ohne dass ihm hieraus Nachteile erwachsen.

Das Original der schriftlichen Einwilligung wird im Studienordner des Prüfzentrums verwahrt. Dem Patienten wird eine Kopie der schriftlichen Patientenaufklärung sowie der Einwilligungserklärung ausgehändigt.

Patienteninformation und Einwilligungserklärung sind als Anlage beigefügt.

Patienteninformation und Einwilligungserklärung, alle weiteren Unterlagen, die Teilnehmer erhalten sowie evtl. Rekrutierungsanzeigen werden vor Verwendung der zuständigen Ethikkommission zur zustimmenden Bewertung vorgelegt.

Über öffentliche Medien, Aushänge und Flyer in HNO-, MKG-Kliniken/-Praxen, Onkologische Zentren und über Veröffentlichungen auf der Homepage der teilnehmenden Kliniken/Praxen wird auf die Studie hingewiesen und diese publik gemacht (Anlage 10). Weiterhin wird über Poster und Flyer, welche auch an Selbsthilfegruppen versendet werden, die Studie beworben.

Patienten in der Nachsorge, welche für die Studie qualifizieren, werden direkt vom behandelnden Arzt/von der behandelnden Ärztin auf die Studie angesprochen. Alle Patienten, die potentiell als Studienteilnehmer in Frage kommen und an der Studie teilnehmen möchten, werden über die Studie informiert und aufgeklärt. Die Teilnahme an der Studie ist freiwillig. Die Vertraulichkeit der Daten wird stets beachtet und garantiert.

5.4. Probandenversicherung

Bei der vorliegenden Studie handelt es sich um keine versicherungspflichtige Studie im Sinne des AMG, des MPG oder der Strahlenschutz- bzw. Röntgenverordnung. Es wird keine gesonderte (Wege-)Unfallversicherung abgeschlossen.

5.5. Datenschutz

5.5.1. Schutz persönlicher Daten

Der Studienleiterleiter schützt das Recht von Patienten auf ihre persönlichen Daten und gewährleistet einen verantwortungsvollen Umgang damit. Alle Personen, die im Rahmen der Studie Patientenkontakt haben oder Zugang zu Krankheitsdaten oder anderen Daten bekommen, sind zur Verschwiegenheit verpflichtet. Eine Weitergabe von Daten erfolgt ausschließlich in pseudonymisierter Form, eine Veröffentlichung erfolgt in aggregierter Form. Damit ist eine Rückverfolgung zu einzelnen Personen weitestgehend ausgeschlossen. Alle Studienbeteiligten müssen dafür Sorge tragen, dass Dokumente mit Namensangaben (z. B. die unterzeichneten Einwilligungserklärungen und die Patienten-Identifikationsliste) vertraulich behandelt und vor unbefugtem Zugriff geschützt aufbewahrt werden. Auf Dokumentationsbögen und ggf. anderen Dokumenten, die an Studienbeteiligte weitergeleitet werden, wird lediglich die pseudonymisierte Patienten-Identifikation angegeben.

5.5.2. Speicherung und Weitergabe pseudonymisierter Daten

Die Patienten werden im Rahmen der Aufklärung für die Studie darüber aufgeklärt, dass die erhobenen Daten an die Studienzentrale der Klinik III, Hämatologie, Onkologie und Palliativmedizin der UMR weitergeleitet werden. Die Patienten werden darüber informiert, dass im Rahmen der Studie alle Daten ausschließlich pseudonymisiert an die Studienzentrale der UMR weitergeleitet, dort gespeichert und für wissenschaftliche Auswertungen und Publikationen verwendet werden. Es werden keine personenbezogenen Daten versendet. Personen, die der Weitergabe ihrer Daten nicht zustimmen, können nicht an der Studie teilnehmen.

6. Statistische Methoden und Ermittlung der Fallzahl

6.1. Statistischer und analytischer Plan

Die Schätzung der Drop-out Rate erfolgt zur Bestimmung des primären Outcomes der Phase I (Machbarkeit).

Die Statistik der primären und sekundären Outcomes (Phase II) umfasst: deskriptive Statistiken, Berechnung der mittleren individuellen Differenzen und Testung auf Null; t-Test für abhängige Stichproben/ Wilcoxon/McNemar), Verteilungsprüfung auf Gleichheit χ^2-Test; Risikobewertung bzw. Ermittlung von Assoziationsmaßen und Testung auf Null; Korrelation/Regression und Varianzanalyse mit Messwiederholung.

6.1.1. Studienpopulationen

Es werden nur Patienten eingeschlossen, die eine endstellig kodierte Diagnose gemäß ICD: C00 - C14, C30 - C32 Bösartige Neubildungen der Lippe, der Mundhöhle, des Rachens, der oberen Atemwege („Kopf-Hals-Tumore") erhalten haben. Es werden keine besonders schutzbedürftigen Personen eingeschlossen.

6.1.2. Beschreibung des Patientenkollektivs

Es sollen nach Möglichkeit Männer und Frauen im selben Verhältnis in die Studie eingeschlossen werden. Die antineoplastische Therapie (Radiatio, Chirurgie, Chemo- oder Immuntherapie) muss mind. seit 4 Monaten abgeschlossen sein (bzw. nach Abschluss der Anschlussheilbehandlung).

6.1.3. Primäres Ziel

Diese Studie besteht aus zwei Phasen.

Phase I (Machbarkeit & Lebensqualität, n = 25):
Es wird die Hypothese überprüft, dass der Anteil der Patienten, die die Trainingsintervention im Home-based Setting über 12 Wochen durchführen, ≥ 70% ist.

Phase II (Lebensqualität, n = 35, gesamt n = 60):
Es wird die Hypothese überprüft, dass sich der globale QoL-Score (EORTC-QLQ-30) nach einer 12-wöchigen Trainingsintervention im Home-based Setting signifikant erhöht.

6.1.4. Sekundäre Ziele

Auf Grund der Komplexität der Studie gibt es mehre sekundäre Outcomes, die im Folgenden aufgelistet sind:

- Gründe für einen vorzeitigen Abbruch der 12-wöchigen Trainingsintervention
- Häufigkeit und Dauer des Trainings im Home-based Setting im Durchschnitt pro Woche (Trainingsumfang)
- Mittelfristige Veränderung der QoL nach einer 12-wöchigen Trainingsintervention im Home-based Setting

- Kurzfristige Veränderung der Beweglichkeit nach einer 12-wöchigen Trainingsintervention im Home-based Setting
- Mittelfristige Veränderung der Beweglichkeit nach einer 12-wöchigen Trainingsintervention im Home-based Setting
- Kurzfristige Veränderung der körperlichen Aktivität nach einer 12-wöchigen Trainingsintervention im Home-based Setting
- Mittelfristige Veränderung der körperlichen Aktivität nach einer 12-wöchigen Trainingsintervention im Home-based Setting
- Kurzfristige Veränderung der Gleichgewichtsfähigkeit /Gangsicherheit nach einer 12-wöchigen Trainingsintervention im Home-based Setting
- Mittelfristige Veränderung der Gleichgewichtsfähigkeit /Gangsicherheit nach einer 12-wöchigen Trainingsintervention im Home-based Setting
- Kurzfristige Veränderung der aeroben Ausdauerleistungsfähigkeit nach einer 12-wöchigen Trainingsintervention im Home-based Setting
- Mittelfristige Veränderung der aeroben Ausdauerleistungsfähigkeit nach einer 12-wöchigen Trainingsintervention im Home-based Setting
- Kurzfristige Veränderungen in der Körperzusammensetzung nach einer 12-wöchigen Trainingsintervention im Home-based Setting
- Mittelfristige Veränderungen in der Körperzusammensetzung nach einer 12-wöchigen Trainingsintervention im Home-based Setting
- Zusammenhänge zwischen dem Trainingsumfang während der 12-wöchigen Trainingsintervention und den Effekten hinsichtlich QoL
- Zusammenhänge zwischen dem Trainingsumfang während der 12-wöchigen Trainingsintervention und den Effekten hinsichtlich der Funktionalität
- Zusammenhänge zwischen dem Trainingsumfang während der 12-wöchigen Trainingsintervention und den Effekten hinsichtlich der Körperzusammensetzung
- Zusammenhänge zwischen Alltagsaktivität und QoL
- Zusammenhänge zwischen Alltagsaktivität und Funktionalität

Zusammenhänge zwischen Alltagsaktivität und Körperzusammensetzung

6.1.5. Subgruppenanalysen

Effekte der Home-based Intervention hinsichtlich QoL, Funktionalität und körperliche Aktivität sollen mittels Subgruppenanalysen in Abhängigkeit von Geschlecht und Trainingsumfang analysiert werden.

6.2. Ermittlung der Fallzahl

Die Fallzahlkalkulation der Phase I basiert auf der Berechnung, dass bei n = 24 eine 30%ige Dropout Rate mit einer Genauigkeit von ± 18% auf einem Konfidenzlevel 1-α = .95 (2-seitig) geschätzt werden kann (nQuery® Advisor 7.0 Statistical Solutions Ltd., Boston, MA, USA).

Für die Fallzahlkalkulation der Phase II wurden die Ergebnisse unserer Pilotstudie (siehe Kapitel 2.2) als Referenzdaten herangezogen (globale QoL pre: 50,1 ± 16,4; post: 58,3 ± 16,2; r = 0,618) und ergab für die Effektstärke von 0,57549 und einem Konfidenzlevel 1-α = .95 (2-seitig) n = 42 (G*Power). Unter Berücksichtigung einer 30%igen Drop-out Rate ist somit mind. eine Fallzahl von n = 60 erforderlich.

7. Sicherheit

Zum Auftreten von Zwischenfällen, sogenannten „Advers Events (AE)" in der Routineversorgung mit onkologischen Patienten liegen bislang nur wenige Daten vor. Bisher wurde das Auftreten von AEs im Training von onkologischen Patienten überwiegend im Rahmen von klinischen Studien erhoben. Auf Grundlage bisheriger Ergebnisse gilt das Training mit onkologische Patienten als gut durchführbar und sicher [19,20], da die Wahrscheinlichkeit für das Auftreten eines „unerwünschten Ereignisses" als niedrig eingeschätzt werden kann [21].

Im Rahmen dieser Studie wird keine Medikation verabreicht. Alle Untersuchungen im Rahmen der Leistungsdiagnostik sind nicht-invasiv. Das Training erfolgt im Home-based Setting. Die empfohlenen Übungen werden dem individuellen Gesundheitszustand und der aktuellen Leistungsfähigkeit der einzelnen Patienten angepasst. Die Trainingszeiten, der Trainingsumfang und die Trainingsintensität werden von den Studienteilnehmern eigenständig festgelegt. Es wird ausdrücklich darauf hingewiesen, dass bei Übelkeit/Erbrechen, starken Schmerzen, Bewusstseinseinschränkungen und Verwirrtheit, bei Schwindel, Kreislaufbeschwerden, bei Fieber > 38° bzw. bei starkem Infekt kein Training absolviert werden sollte. Trotzdem sind auf Grund der ungewohnten Belastung Zwischenfälle (z. B. Übelkeit/Erbrechen, muskuläre Beschwerden, Kreislaufbeschwerden) nicht auszuschließen.

Um eventuell auftretende Zwischenfälle zu erfassen, werden während der Home-based Intervention die Patienten einmal wöchentlich vom lokalen Therapeuten angerufen und der Status quo erfragt und Zwischenfälle die vermeintlich im Zusammenhang mit dem Home- based Training standen dokumentiert. Dabei wird zwischen AEs und SAEs unterschieden.

7.1. Definitionen für unerwünschte Ereignisse

Aktuell gibt es für Zwischenfälle beim Training mit onkologischen Patienten keine eindeutige Definition. Der ursprünglich in der Medizin etablierte Begriff „Adverse Events" wird benutzt, um unerwünschte Zwischenfälle während Medikamentenstudien zu erfassen und zu analysieren. Im Bereich der onkologischen Bewegungstherapie sind die Definitionen Adverse Events anders zu definieren als in Medikamentenstudien. Um verschiedene Schweregrade zu klassifizieren werden Serious Adverse Events (SAEs) und Non-Serious Adverse Events (AEs) wie folgt definiert:

7.1.1. Unerwünschtes Ereignis (AE)

„Es handelt sich um gesundheitliche Zwischenfälle, die zu einer kurzfristigen Beeinträchtigung der körperlichen Funktionsfähigkeit oder Gesundheit führen und höchstens ärztlich abgeklärt werden, aber nicht behandlungsbedürftig sind" [21].

Dabei muss das AE nicht notwendigerweise in ursächlichem Zusammenhang mit dem Training stehen. Als AEs werden alle Ereignisse erfasst, die während des Trainings (inkl. Leistungsdiagnostik) auftreten.

Zu den AEs zählen beispielsweise auftretender Schwindel, Übelkeit oder Erbrechen während des Trainings bzw. der Leistungsdiagnostik. Ebenso zählen durch das Training verursachte Verstauchungen, Bänderüberdehnungen, Muskelfaserrisse und ähnliches dazu.

7.1.2. Schwerwiegendes unerwünschtes Ereignis (SAE)

„Es handelt sich um gesundheitliche Zwischenfälle im Training, die eine mittel- oder langfristige Beeinträchtigung der körperlichen Funktionsfähigkeit oder Gesundheit zur Folge haben und eine ärztliche Behandlung nach sich ziehen (Tod, reanimierungspflichtiger Herzkreislaufstillstand, Knochenbrüche, Narbenbrüche und Verletzungen).“ [21]

SAEs während der Ausübung der Studie sind Vorkommnisse, die

a) tödlich oder lebensbedrohend sind,

b) eine außerplanmäßige stationäre Behandlung erforderlich machen und/oder

c) eine mittelfristige gesundheitliche Beeinträchtigung mit ärztlicher Behandlung erforderlich machen (z. B. konventionell therapierter Knochenbruch)

Dabei muss das SAE nicht notwendigerweise in ursächlichem Zusammenhang mit dem Training stehen. Als lebensbedrohlich im obigen Zusammenhang werden Ereignisse betrachtet, bei denen die Gefahr zu sterben zum Zeitpunkt des Ereignisses bestand.
Als Krankenhausaufnahme wird jeder stationäre Aufenthalt eines Studien-Teilnehmers angesehen, der mindestens eine Nacht (22 – 6 Uhr) umfasst hat.

7.2. Dokumentation und Nachverfolgung von AEs/SAEs

Während der 12-wöchigen Trainingsintervention, die im Home-based Setting erfolgt, werden alle Studienteilnehmer einmal wöchentlich von ihrem lokalen Therapeuten angerufen. Dieser erfragt aufgetretene unerwünschte Ereignisse (AEs und SAEs) während der Trainingsausübung und dokumentiert diese in einem „Telefonprotokoll“ (Anlage 8). Dabei soll auch erfasst werden, wobei das Ereignis aufgetreten ist (Ausdauertraining, Krafttraining, Gleichgewichtstraining etc.).

Nach Abschluss der Trainingsintervention erfolgt die Zusendung der Protokolle an die Studienleitung/Studienzentrale der UMR, wo die Zusammenführung aller dokumentierten AEs/SAEs erfolgt.

In der Nachbeobachtungszeit (12-wöchiges Follow Up) informieren die Studienteilnehmer ihren Therapeuten über aufgetretene Zwischenfälle beim Training. Der Therapeut benachrichtigt in diesen Fällen die Studienzentrale der Klinik III, Hämatologie, Onkologie und Palliativmedizin der UMR per FAX oder E-Mail, nachdem er informiert wurde.

8. Verwendung der Daten und Publikation

8.1. Berichte

8.1.1. Zwischenberichte

Ein Zwischenbericht wird bei 25 in die Studie eingeschlossenen Patienten erstellt und dem Projektleiter und allen Prüfern der teilnehmenden Prüfzentren vorgelegt.

8.1.2. Abschlussbericht

Ein Abschlussbericht wird bis drei Monate nach Studienende verfasst und dem Projektleiter und allen Prüfern der teilnehmenden Prüfzentren vorgelegt.

8.2. Publikation

Es ist vorgesehen, die Ergebnisse der Studie zu gegebener Zeit in wissenschaftlichen Fachzeitschriften und/oder bei deutschen und internationalen Kongressen vorzustellen. Für alle Veröffentlichungen gilt, dass der Datenschutz sowohl für alle Daten von betroffenen Personen als auch für die Daten der teilnehmenden Prüfer gewahrt bleibt.

Auch eine Registrierung der Studie in einem öffentlichen Register entsprechend der Empfehlungen des „International Committee of Medical Journal Editors“ (ICMJE) ist vorgesehen.

Die Veröffentlichung oder ein Vortrag der Ergebnisse aus dieser Studie, einschließlich einer Veröffentlichung oder eines Vortrags eines einzelnen Prüfzentrums, bedürfen der vorherigen Kenntnisnahme und einer vorgehenden Kommentierung und Genehmigung durch den Sponsor. Die Anzahl und Reihenfolge der Autoren bei Publikationen wird/ist im Kooperationsvertrag geregelt.

9. Änderungen des Prüfplans

Zum Zweck der Sicherstellung weitgehend vergleichbarer Bedingungen im Interesse einer einwandfreien Datenauswertung ist eine Änderung der vereinbarten und im Prüfplan niedergelegten Prüfungsbedingungen nicht vorgesehen. In Ausnahmefällen sind jedoch Änderungen der Prüfungsbedingungen möglich. Diese erfolgen nur nach gegenseitiger Abstimmung zwischen dem Sponsor, dem Sponsorvertreter, sowie allen Unterzeichnenden (Autoren) dieses Prüfplans. Jede Änderung der im Prüfplan vorgesehenen Studienprozeduren muss schriftlich unter Angabe der jeweiligen Gründe erfolgen und von allen Autoren dieses Prüfplans unterschrieben werden (Amendment).

Nach § 10 Abs. 1 und 4 GCP-V genehmigungspflichtige nachträgliche Änderungen werden der Ethikkommission zur Genehmigung vorgelegt und erst nach deren Genehmigung umgesetzt. Hiervon unbenommen sind Änderungen, die zur Abwendung unmittelbarer Gefahr notwendig sind.

10. Anhang

Anlage 1	Fragebögen MMSE und Erfassung krankheitsspezifischer Daten
Anlage 2	Allgemeiner Fragebogen: Erfassung personenbezogener Daten
Anlage 3	Fragebögen EORTC QLQ-30 und EORTC H&N35
Anlage 4	Fragebogen GSLTPAQ
Anlage 5	Abschlussfragebogen
Anlage 6	Protokoll Funktionsdiagnostik inkl. POMS-F, BORG- und CR-10-Skala
Anlage 7	Trainingstagebuch mit Informationen zum Training
Anlage 8	Telefonprotokoll
Anlage 9	Übersicht der Aufgaben der Therapeuten
Anlage 10	Flyer und Poster

11. Literatur

[1] B. W. Stewart and C. P. Wild, "World Cancer Report 2014," EBL-Schweitzer, International Agency for Research on Cancer/World Health Organization, Lyon, 2014.

[2] Robert Koch-Institut (RKI), "Bericht zum Krebsgeschehen in Deutschland 2016,"

[3] E. J. Borghardt, "Rehabilitation bei Patienten mit Kopf-Halstumoren," In: H.-J. Schmoll, Ed., *Indikationen, Therapiekonzepte und spezielle Therapiemodalitäten*. 4, Springer Medizin Verl., Heidelberg, 2006, pp. 1363–1372.

[4] M. Couch, V. Lai, T. Cannon, D. Guttridge, A. Zanation, J. George et al., "Cancer cachexia syndrome in head and neck cancer patients: part I. Diagnosis, impact on quality of life and survival, and treatment," *Head & neck*, Vol. 29, No. 4, 2007, pp. 401–411. doi:10.1002/hed.20447.

[5] J. B. Epstein, M. Robertson, S. Emerton, N. Phillips and P. Stevenson-Moore, "Quality of life and oral function in patients treated with radiation therapy for head and neck cancer," *Head & neck*, Vol. 23, No. 5, 2001, pp. 389–398.

[6] J. A. Langendijk, P. Doornaert, I. M. Verdonck-de Leeuw, C. R. Leemans, N. K. Aaronson and B. J. Slotman, "Impact of late treatment-related toxicity on quality of life among patients with head and neck cancer treated with radiotherapy," *Journal of clinical oncology*, Vol. 26, No. 22, 2008, pp. 3770–3776. doi:10.1200/JCO.2007.14.6647.

[7] M. Eades, J. Murphy, S. Carney, S. Amdouni, J. Lemoignan, M. Jelowicki et al., "Effect of an interdisciplinary rehabilitation program on quality of life in patients with head and neck cancer: review of clinical experience," *Head & neck*, Vol. 35, No. 3, 2013, pp. 343–349. doi:10.1002/hed.22972.

[8] L. C. Capozzi, K. R. Boldt, H. Lau, L. Shirt, B. Bultz and S. N. Culos-Reed, "A clinic-supported group exercise program for head and neck cancer survivors: managing cancer and treatment side effects to improve quality of life," *Sports medicine*, Vol. 23, No. 4, 2015, pp. 1001–1007. doi:10.1007/s00520-014-2436-4.

[9] L. C. Capozzi, K. C. Nishimura, M. L. McNeely, H. Lau and S. N. Culos-Reed, "The impact of physical activity on health-related fitness and quality of life for patients with head and neck cancer: a systematic review," *British journal of sports medicine*, Vol. 50, No. 6, 2016, pp. 325–338. doi:10.1136/bjsports-2015-094684.

[10] S. Lønbro, U. Dalgas, H. Primdahl, J. Overgaard and K. Overgaard, "Feasibility and efficacy of progressive resistance training and dietary supplements in radiotherapy treated head and neck cancer patients-the DAHANCA 25A study," *Acta oncologica*, Vol. 52, No. 2, 2013, pp. 310–318. doi:10.3109/0284186X.2012.741325.

[11] M. L. McNeely, M. Parliament, K. S. Courneya, H. Seikaly, N. Jha, R. Scrimger et al., "A pilot study of a randomized controlled trial to evaluate the effects of progressive resistance exercise training on shoulder dysfunction caused by spinal accessory neurapraxia/neurectomy in head and neck cancer survivors," *Head & neck*, Vol. 26, No. 6, 2004, pp. 518–530. doi:10.1002/hed.20010.

[12] M. L. McNeely, M. B. Parliament, H. Seikaly, N. Jha, D. J. Magee, M. J. Haykowsky et al., "Effect of exercise on upper extremity pain and dysfunction in head and neck cancer survivors: a randomized controlled trial," *Cancer*, Vol. 113, No. 1, 2008, pp. 214–222. doi:10.1002/cncr.23536.

[13] N. Pauli, B. Fagerberg-Mohlin, P. Andréll and C. Finizia, "Exercise intervention for the treatment of trismus in head and neck cancer," *Acta oncologica*, Vol. 53, No. 4, 2014, pp. 502–509. doi:10.3109/0284186X.2013.837583.

[14] L. Sammut, M. Ward and N. Patel, “Physical activity and quality of life in head and neck cancer survivors: a literature review,” *International journal of sports medicine*, Vol. 35, No. 9, 2014, pp. 794–799. doi:10.1055/s-0033-1363984.
[15] A. W. Midgley, D. Lowe, A. R. Levy, V. Mepani and S. N. Rogers, “Exercise program design considerations for head and neck cancer survivors,” *European archives of oto-rhino-laryngology*, Vol. 275, No. 1, 2018, pp. 169–179. doi:10.1007/s00405-017-4760-z.
[16] L. Q. Rogers, J. Malone, K. Rao, K. S. Courneya, A. Fogleman, A. Tippey et al., “Exercise preferences among patients with head and neck cancer: prevalence and associations with quality of life, symptom severity, depression, and rural residence,” *Head & neck*, Vol. 31, No. 8, 2009, pp. 994–1005. doi:10.1002/hed.21053.
[17] S. Felser, M. Behrens, Strüder D, Liese J, Rohde K, Junghanss C et al., “Feasibility and Effects of a Supervised Exercise Program Suitable for Independent Training at Home on Physical Function and Quality of Life in Head and Neck Cancer Patients: A Pilot Study,” *Integrative cancer therapies*, Vol. 19, 2019, 1-12. doi:10.1177/15347354209189.
[18] S. Felser, M. Behrens, K. Rhode, Liese J, D. Strüder, C. Junghanss et al., “Effects of a 12-week low to moderate intensity exercise intervention on physical function and quality of life in head and neck cancer survivors.,” *P388, Abstract-USB-Stick Jahrestagung der Deutschen, Österreichischen und Schweizerischen Gesellschaften für Hämatologie und Medizinische Onkologie*, 2019, ISSN 1863-1819.
[19] R. Heywood, A. L. McCarthy and T. L. Skinner, “Safety and feasibility of exercise interventions in patients with advanced cancer: a systematic review,” *Supportive care in cancer official journal of the Multinational Association of Supportive Care in Cancer*, Vol. 25, No. 10, 2017, pp. 3031–3050. doi:10.1007/s00520-017-3827-0.
[20] B. Singh, R. R. Spence, M. L. Steele, C. X. Sandler, J. M. Peake and S. C. Hayes, “A Systematic Review and Meta-Analysis of the Safety, Feasibility, and Effect of Exercise in Women With Stage II+ Breast Cancer,” *Archives of physical medicine and rehabilitation*, Vol. 99, No. 12, 2018, pp. 2621–2636. doi:10.1016/j.apmr.2018.03.026.
[21] D. Clauss, F. Quirmbach, J. Wiskemann and F. Rosenberger, “Adverse Events beim Training mit onkologischen Patienten: Wie sicher ist das Training außerhalb klinischer Studien?,” Vol. 2019, No. 04, 35, pp. 194–201. doi:10.1055/a-9057-1883.

Mini-Mental State Examination (MMSE)
Handlungsanweisung und Ergebnisinterpretation

Zur Abgrenzung einer Altersvergesslichkeit, einer Demenz oder Depression wird häufig die Mini-Mental State Examination (MMSE) durchgeführt. Dieser Test ist Teil einer ausführlichen Diagnostik zur Überprüfung von Fähigkeiten und kognitiven Einschränkungen.

Er besteht aus einem Interview mit Handlungsaufgaben und praxisnahen Fragen, die von kognitiv nicht beeinträchtigten Personen im Regelfall problemlos zu beantworten sind, von Personen mit Verdacht auf Alzheimer oder eine andere Demenz dagegen nur zum Teil bewältigt werden.

Mit den Aufgaben werden räumliche Orientierung, Merkfähigkeit, Erinnerungsfähigkeit, Aufmerksamkeit, Rechenfähigkeit und Sprache geprüft.

Die MMSE dauert etwa 10 - 15 Minuten. Jede richtige Antwort wird als ein Punkt gezählt. Maximal werden 30 Punkte vergeben.

[in Anlehnung an die Alzheimer Forschung Initiative e.V.]
https://www.alzheimer-forschung.de/alzheimer/diagnose/psychometrische-tests/mmst/

Allgemeine Vorbemerkungen zur MMSE

- Vor der Testung sollte das Einverständnis des Patienten in mündlicher Form eingeholt werden
- Der Test ist an einem ruhigen, ungestörten Ort durchzuführen
- Die Items sind in der vorgeschriebenen Reihenfolge durchzugehen
- Da die Fragen zum Teil sehr einfach sind, sollte bei kognitiv scheinbar wenig gestörten Probanden eine Vorbemerkung erfolgen, wie z.B.: „Einiges von dem, was ich Sie jetzt frage, ist für Sie wahrscheinlich zu einfach, aber es gehört zur routinemäßigen Untersuchung“
- Bei Hör- und Verständnisschwierigkeiten dürfen die Fragen wiederholt werden, es darf jedoch keine Hilfe bei der Beantwortung geleistet werden
- Der Patient soll sich wohlfühlen
- Eine positive Verstärkung ist erlaubt und erwünscht („das machen Sie gut“), Hinweise auf die Richtigkeit der Antwort sind jedoch zu unterlassen („ja, das ist richtig“)

Zur Durchführung des Tests Folgendes bereithalten:

- Eine Armbanduhr & einen Bleistift/Kugelschreiber

MMSE-Ergebnisinterpretation:

- ***Zwischen 30 und 25 Punkten:***
  Es ist keine kognitive Einschränkung anzunehmen.

- ***Zwischen 24 und 18 Punkten:***
  Es kann eine leichte kognitive Einschränkung angenommen werden.

- ***Zwischen 17 und 0 Punkten:***
  Eine schwere bis schwerste kognitive Einschränkung ist wahrscheinlich.

Im Rahmen der HeiKo-Studie wird ein Patient zur Teilnahme zugelassen, sofern er ein MMSE-Ergebnis von ≥ 24 Punkten erreicht.

Literatur:
M. F. Folstein, S. E. Folstein, P. R. McHugh: Mini-Mental State. A practical method for grading the state of patients for the clinician. In: Journal of Psychiatric Research. 12, 1975, S. 189–198.

Handlungsanleitung: Mini-Mental State Examination

Punkte
(1-5) (0/1) 1. Was für ein Datum ist heute?
(0/1) 2. Welche Jahreszeit?
(0/1) 3. Welches Jahr haben wir?
(0/1) 4. Welcher Wochentag ist heute?
(0/1) 5. Welcher Monat?

Zuerst nach dem Datum fragen, dann gezielt nach den noch fehlenden Punkten (z. B. „Können Sie mir auch sagen, welche Jahreszeit jetzt ist?").

(6-10) (0/1) 6. Wo sind wir jetzt?
Welches Bundesland?
(0/1) 7. Welcher Landkreis/welche Stadt?
(0/1) 8. Welche Stadt/welcher Stadtteil?
(0/1) 9. Welches Krankenhauswelche Klinik/Praxis?
(0/1) 10.Welche Station/welche Etage?

Zuerst nach Stadt, dann nach Stadtteil usw. fragen. In Großstädten sollte nicht nach Stadt und Landkreis, sondern nach Stadt und Stadtteil gefragt werden, in jedem Fall nach dem aktuellen Aufenthaltsort und nicht nach dem Wohnort.

(11-13) Bitte merken Sie sich:
(0/1) 11. Apfel
(0/1) 12. Pfennig
(0/1) 13. Tisch

Der Untersucher muss zuerst fragen, ob der Patient mit einem kleinen Gedächtnistest einverstanden ist. Er wird darauf hingewiesen, dass er sich 3 Begriffe merken soll.

Die Begriffe langsam und deutlich – im Abstand von jeweils ca. einer Sekunde nennen. Direkt danach die 3 Begriffe wiederholen lassen, der erste Versuch bestimmt die Punktzahl. Ggf. wiederholen, bis der Untersuchte alle 3 Begriffe gelernt hat.

Die Anzahl der notwendigen Versuche wird notiert (max. 6 Versuche sind zulässig). Wenn nicht alle 3 Begriffe gelernt wurden, kann der Gedächtnistest nicht durchgeführt werden.

(14-18) Ziehen Sie von 100 jeweils 7 ab oder – falls nicht durchführbar – buchstabieren Sie Stuhl rückwärts:

(0/1)	14.	93	L
(0/1)	15.	86	H
(0/1)	16.	79	U
(0/1)	17.	72	T
(0/1)	18.	65	S

Beginnend bei 100 muss fünfmal jeweils 7 subtrahiert werden. Jeden einzelnen Rechenschritt unabhängig vom vorhergehenden bewerten, damit ein Fehler nicht mehrfach bewertet wird.

Alternativ (z. B. wenn der Untersuchte nicht rechnen kann oder will) kann in Ausnahmefällen das Wort STUHL rückwärts buchstabiert werden. Das Wort sollte zunächst vorwärts buchstabiert und – wenn nötig – korrigiert werden.

Die Punktzahl ergibt sich aus der Anzahl der Buchstaben, die in der richtigen Reihenfolge genannt werden (z. B. ,,LHTUS“ = 3 Punkte, da „L“, „H“ und „S“ an korrekter Stelle genannt wurden).

(19-21) Was waren die Dinge, die Sie sich vorher gemerkt haben?

(0/1) 19. Apfel
(0/1) 20. Pfennig
(0/1) 21. Tisch

Der Untersuchte muss die drei Begriffe nennen, die er sich unter (11-13) merken sollte.

(22,23) Was ist das?

(0/1) 22. Armbanduhr
(0/1) 23. Bleistift/Kugelschreiber

Eine Armbanduhr und ein Stift werden gezeigt, der Untersuchte muss diese richtig benennen.

(24) Sprechen Sie nach:
(0/1) 24. „Sie leiht ihm kein Geld mehr.“

Der Satz muss unmittelbar nachgesprochen werden.

(25-27) Machen Sie bitte Folgendes:

(0/1) 25. Nehmen Sie bitte das Papier in die Hand,
(0/1) 26. falten Sie das Papier in der Mitte und
(0/1) 27. lassen Sie das Papier auf den Boden fallen.

Der Untersuchte erhält ein Blatt Papier, der dreistufige Befehl wird nur <u>einmal</u> erteilt. Einen Punkt gibt es für jeden Teil, der korrekt befolgt wird.

(28) Lesen Sie und machen Sie es bitte:

(0 /1) 28. „Bitte schließen Sie die Augen“

Die schriftliche Anweisung („Bitte schließen Sie die Augen“) muss so groß sein, dass sie auch bei eingeschränktem Visus noch lesbar ist (s. Anhang). Ein Punkt wird nur dann gegeben, wenn die Augen wirklich geschlossen werden.

(29) (0/1) 29. Schreiben Sie bitte einen vollständigen Satz (mind. Subjekt und Prädikat)!

Es darf kein Satz diktiert werden. Die Ausführung muss spontan erfolgen. Der Satz muss Subjekt und Prädikat enthalten und sinnvoll sein. Korrekte Grammatik und Interpunktion ist nicht gefordert. Das Schreiben von Namen und Anschrift ist nicht ausreichend.

(30) (0 /1) 30. Kopieren Sie bitte die Zeichnung (zwei Fünfecke).

Auf einem Blatt sind zwei sich überschneidende Fünfecke dargestellt (s. Anhang). Der Untersuchte soll diese so exakt wie möglich abzeichnen. Alle 10 Ecken müssen widergegeben sein und zwei davon sich überschneiden, nur dann wird ein Punkt gegeben.

Mini-Mental State Examination (MMSE)

Code: ____________________

Datum: __________________ Erhebung durchgeführt von: __________________

I. Orientierung

Hinweis	Nr.	Punkte (richtig: 1 | falsch: 0)	1	0
Beispielfragen: Welches Datum haben wir heute? Welche Jahreszeit haben wir aktuell?	1	Datum	◻	◻
	2	Jahr	◻	◻
	3	Jahreszeit	◻	◻
	4	Wochentag	◻	◻
	5	Monat	◻	◻
	6	Bundesland	◻	◻
	7	Landkreis/Stadt	◻	◻
	8	Stadt/Stadtteil	◻	◻
	9	Krankenhaus/Klinik/Praxis	◻	◻
	10	Station/Etage	◻	◻
Punkte (max. 10)		Summe:		

II. Merkfähigkeit

Hinweis	Nr.	Punkte (richtig: 1 | falsch: 0)	1	0
Nennung der Gegenstände und Aufforderung zur Wiederholung	11	Apfel	◻	◻
	12	Pfennig	◻	◻
	13	Tisch	◻	◻
Punkte (max. 3)		Summe:		

III. Aufmerksamkeit / Rechenfähigkeit

Hinweis	Nr.	Antworten — Punkte (richtig: 1 | falsch: 0)	1	0
Ziehen Sie von 100 jeweils 7 ab oder buchstabieren Sie „STUHL“ rückwärts.	14	„93“ oder „L“	◻	◻
	15	„86“ oder „H“	◻	◻
	16	„79“ oder „U“	◻	◻
	17	„72“ oder „T“	◻	◻
	18	„65“ oder „S“	◻	◻
Punkte (max. 5)		Summe:		

IV. Erinnerungsfähigkeit

Hinweis	Nr.	Punkte (richtig: 1 | falsch: 0)	1	0
Was waren die Dinge, die Sie sich vorher gemerkt haben?	19	Apfel	◻	◻
	20	Pfennig	◻	◻
	21	Tisch	◻	◻
Punkte (max. 3)		Summe:		

V. Sprache

Hinweis	Nr.	Punkte (richtig: 1 | falsch: 0)	1	0
Was ist das? Zeigen von zwei Gegenstän-den und Aufforderung zur Benennung	22	Armbanduhr	◻	◻
	23	Bleistift / Kugelschreiber	◻	◻
Aufforderung zum Nachsprechen (3 Versuche)	24	„Sie leiht ihm kein Geld mehr.“	◻	◻
Kommandos befolgen ▪ mündlich	25	Nehmen Sie bitte das Papier in die Hand,	◻	◻
	26	falten Sie das Papier in der Mitte und	◻	◻
	27	lassen Sie das Papier auf den Boden fallen.	◻	◻
▪ schriftlich	28	Bitte schließen Sie die Augen.	◻	◻
	29	Schreiben Sie bitte einen vollständigen Satz.	◻	◻
	30	Kopieren Sie bitte die Zeichnung (zwei Fünfecke).	◻	◻
Punkte (max. 9)		Summe:		

Ergebnis des MMSE-Tests

Gesamtsumme (max. 30 Punkte):	

◻	≥ 24 Punkte	Der/die Patient/in kann an der HeiKo-Studie teilnehmen.
◻	< 24 Punkte	Der/die Patient/in kann **nicht** an der HeiKo-Studie teilnehmen.

Bitte schließen
Sie die Augen.

Kopieren Sie bitte die folgende Zeichnung:

2 sich überschneidende Fünfecke:

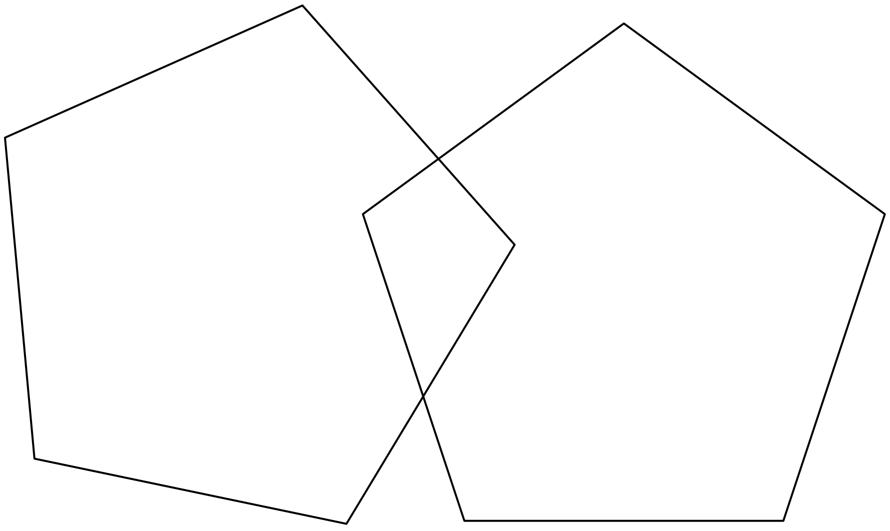

Abgezeichnet:

Fragebogen zur Studie:
„Individuelles Heimtraining für Patienten mit Kopf-Halstumoren –
eine multizentrische Studie zur Verbesserung der Lebensqualität“

Auszufüllen vom ärztlichen Personal

Code Studienteilnehmer: ___________________________

Diagnose: ____

Datum /Jahr der Erstdiagnose: ____ . ____ .______

Stadium bei Diagnose: ________________

aktuelle Tumorsituation:

☐1 partielle Remission
☐2 komplette Remission
☐3 sonstiges: ______________________________

bisherige medizinische Therapie(n):

☐1 Operation ☐ neck dissection
☐2 Strahlentherapie ☐ primäre ☐ adjuvant
☐3 primäre Radio-/Chemotherapie
☐4 Radio-/Immuntherapie
☐5 sonstiges: ____

Sonstige Hinweise / Ergänzungen:

____
____
____
____

____
Unterschrift Arzt

Fragebogen zur Studie:
„Individuelles Heimtraining für Patienten mit Kopf-Halstumoren –
eine multizentrische Studie zur Verbesserung der Lebensqualität“

Sehr geehrte Studienteilnehmerin, sehr geehrter Studienteilnehmer,

wir freuen uns, dass Sie sich dazu entschlossen haben, an unserer o. g. Studie teilzunehmen. Bitte füllen Sie die vorliegenden Fragebögen vollständig aus. Kreuzen Sie jeweils die Antwort an, die auf Sie zutrifft bzw. die Ihrer Vorstellung am ehesten entspricht. Es gibt keine „richtigen“ oder „falschen“ Antworten. Tragen Sie Ihre Antworten bitte leserlich in die dafür vorgesehenen Felder bzw. markieren Sie die gültigen bzw. korrigierten Antworten wie folgt: gültige Antwort: ☒ bei Korrekturen die ungültige Antwort: ☒.

Wir versichern Ihnen, dass die Daten vertraulich und nur für wissenschaftliche Zwecke verwendet werden. Die Rückverfolgung zu Ihrer Person ist für die Studienleitung und das Datenmanagement ausgeschlossen. Zu diesem Zweck haben wir einen Code generiert, über den die Zusammenführung Ihrer Daten pseudonymisiert über die verschiedenen Erhebungszeitpunkte erfolgt.

Code: ____________________________

Datum: _______. _______. __________

A. Allgemeine Angaben zu Ihrer Person

1. Geschlecht

   ☐$_1$ weiblich ☐$_2$ männlich

2. Alter [Jahre] _______

3. Größe [cm] _______

4. Gewicht [kg] _______

5. Familienstand (bitte nur eine Antwort ankreuzen)

   ☐$_1$ ledig / alleinstehend

   ☐$_2$ verheiratet / mit festem Partner zusammenlebend

   ☐$_3$ sonstiges: _______________________________

6. höchster Schulabschluss

   ☐$_1$ kein Abschluss

   ☐$_2$ Volks-/ Hauptschule

   ☐$_3$ mittlere Reife/ Realschule/ POS

   ☐$_4$ Fachoberschule

   ☐$_5$ Abitur

   ☐$_6$ anderer Abschluss: _________________________

7. gegenwärtiger Berufsstatus

   ☐$_1$ berufstätig _____%

   ☐$_2$ in Ausbildung/ Umschulung

   ☐$_3$ arbeitslos

   ☐$_4$ ausschließlich Hausfrau/ -mann

   ☐$_5$ in Rente/ pensioniert

   ☐$_6$ sonstiges: _____________________________________

8. Besuchen Sie regelmäßig eine Selbsthilfegruppe?

   ☐$_1$ ja

   ☐$_2$ nein

9. Wie sind Sie als Erstes auf diese Studie aufmerksam geworden?

   ☐$_1$ durch Selbsthilfegruppe ☐$_2$ Flyer/Aushänge

   ☐$_3$ behandelnder Arzt ☐$_4$ andere Studienteilnehmer

   ☐$_5$ Internetrecherche (Webseite) ☐$_6$ sonstiges: ____________________________

B. Angaben zum Nikotin- und Alkoholkonsum

1. Angaben zum Nikotinkonsum

☐$_1$ Nichtraucher/Nichtdampfer

☐$_2$ Ex-Raucher/Ex-Dampfer, etwa wie lange nicht mehr (Monate /Jahre)? ___________

☐$_3$ Raucher/Dampfer, ich rauche zirka _____ (E-)Zigaretten pro Tag.

2. Angaben zum aktuellen Alkoholkonsum

2. a. Wie oft nehmen Sie alkoholische Getränke zu sich?

☐$_0$ Nie → bitte weiter mit Teil C, Frage 1.

☐$_1$ ≤ 1mal im Monat

☐$_2$ 2-4 mal im Monat

☐$_3$ 2-3 mal pro Woche

☐$_4$ ≥ 4 mal pro Woche

2. b. Wie viele alkoholische Getränke* trinken Sie normalerweise pro Tag?
* z. B. 1 Bier (0,25 l = 1 kleines Glas) oder 1 Wein/Sekt (0,1 l = 1 halbes Weinglas) oder
1 Schnaps/Likör (4 cl = 1 „Doppelter“)

☐$_0$ 1-2 ☐$_1$ 3-4 ☐$_2$ 5-6 ☐$_3$ 7-9 ☐$_4$ ≥ 10

C. Angaben zur Sportvergangenheit

1. Waren Sie vor Ihrer Krebserkrankung sportlich aktiv (Schulsport ausgenommen)? Wenn ja, in welchem Bereich und in welchem Alter?

☐$_1$ Nein, ich war noch nie sportlich aktiv.

☐$_2$ Ja, ich war vor der Erkrankung sportlich aktiv, und zwar im Alter von _____ bis _____.
Folgende Sportarten / Bewegungsformen habe ich betrieben:

____

____

2. a. Üben Sie zurzeit eine *intensive sportliche Aktivität* regelmäßig aus, d. h. für jeweils **20 min an mindestens 3 Tagen** pro Woche?

☐$_1$ Nein.

☐$_2$ Ja, aber erst seit weniger als 6 Monaten.

☐$_3$ Ja, seit mehr als 6 Monaten.

b. Wenn Sie bereits sportlich aktiv sind, geben Sie bitte an, was Sie seit wann machen!

☐1 ____, seit ______________________________

☐2 ____, seit ______________________________

3. Haben Sie <u>während</u> Ihrer Krebstherapie an speziellen Bewegungsprogrammen (z. B. onkologische Trainings- und Bewegungstherapie, Physiotherapie, Vereinssport) teilgenommen?

☐1 Nein.

☐2 Ja. (Bitte ergänzen Sie, <u>was</u> Sie <u>wie lange</u> gemacht haben)

____

____

Haben Sie die Übungen, die Sie dort erlernt haben, eigenständig fortgeführt?

☐1 Nein. Was sind die Gründe: ____

☐2 Ja, aber nur für eine gewisse Zeit. Abbruchsgründe: ______________________________

☐3 Ja, diese Übungen mache ich immer noch regelmäßig.

4. Haben Sie <u>nach</u> Ihrer Krebstherapie an speziellen Bewegungsprogrammen (z. B. Rehabilitationssport, onkologische Trainings- und Bewegungstherapie, Physiotherapie, Vereinssport) teilgenommen?

☐1 Nein.

☐2 Ja. (Bitte ergänzen Sie, <u>was</u> Sie <u>wie lange</u> gemacht haben)

____

____

Haben Sie die Übungen, die Sie dort erlernt haben, eigenständig fortgeführt?

☐1 Nein. Was sind die Gründe: ____

☐2 Ja, aber nur für eine gewisse Zeit. Abbruchsgründe: ______________________________

☐3 Ja, diese Übungen mache ich immer noch regelmäßig.

Vielen Dank für Ihre Teilnahme!

Code: _______________ Datum: _______________

EORTC QLQ 30 (Version 3.0)

Wir sind an einigen Angaben interessiert, die Sie und Ihre Gesundheit betreffen. Bitte beantworten Sie die folgenden Fragen selbst, indem Sie die Zahl ankreuzen, die am besten auf Sie zutrifft. Es gibt keine „richtigen" oder „falschen" Antworten. Ihre Angaben werden streng vertraulich behandelt.

	Überhaupt nicht	Wenig	Mäßig	Sehr
1. Bereitet es Ihnen Schwierigkeiten sich körperlich anzustrengen (z.B. eine schwere Einkaufstasche oder einen Koffer zu tragen?)	1	2	3	4
2. Bereitet es Ihnen Schwierigkeiten, einen <u>längeren</u> Spaziergang zu machen?	1	2	3	4
3. Bereitet es Ihnen Schwierigkeiten, eine <u>kurze</u> Strecke außer Haus zu gehen?	1	2	3	4
4. Müssen Sie tagsüber im Bett liegen oder in einem Sessel sitzen?	1	2	3	4
5. Brauchen Sie Hilfe beim Essen, Anziehen, Waschen oder Benutzen der Toilette?	1	2	3	4

Während der letzten Woche:

	Überhaupt nicht	Wenig	Mäßig	Sehr
6. Waren Sie bei Ihrer Arbeit oder bei anderen tagtäglichen Beschäftigungen eingeschränkt?	1	2	3	4
7. Waren Sie bei Ihren Hobbys oder anderen Freizeitbeschäftigungen eingeschränkt?	1	2	3	4
8. Waren Sie kurzatmig?	1	2	3	4
9. Hatten Sie Schmerzen?	1	2	3	4
10. Mussten Sie sich ausruhen?	1	2	3	4
11. Hatten Sie Schlafstörungen?	1	2	3	4
12. Fühlten Sie sich schwach?	1	2	3	4
13. Hatten Sie Appetitmangel?	1	2	3	4
14. War Ihnen übel?	1	2	3	4
15. Haben Sie erbrochen?	1	2	3	4

Während der letzten Woche:

	Überhaupt nicht	Wenig	Mäßig	Sehr
16. Hatten Sie Verstopfung?	1	2	3	4
17. Hatten Sie Durchfall?	1	2	3	4
18. Waren Sie müde?	1	2	3	4
19. Fühlten Sie sich durch Schmerzen in Ihrem alltäglichen Leben beeinträchtigt?	1	2	3	4
20. Hatten Sie Schwierigkeiten sich auf etwas zu konzentrieren, z.B. auf das Zeitunglesen oder das Fernsehen?	1	2	3	4
21. Fühlten Sie sich angespannt?	1	2	3	4
22. Haben Sie sich Sorgen gemacht?	1	2	3	4
23. Waren Sie reizbar?	1	2	3	4
24. Fühlten Sie sich niedergeschlagen?	1	2	3	4
25. Hatten Sie Schwierigkeiten, sich an Dinge zu erinnern?	1	2	3	4
26. Hat Ihr körperlicher Zustand oder Ihre medizinische Behandlung Ihr Familienleben beeinträchtigt?	1	2	3	4
27. Hat Ihr körperlicher Zustand oder Ihre medizinische Behandlung Ihr Zusammensein oder Ihre gemeinsamen Unternehmungen mit anderen Menschen beeinträchtigt?	1	2	3	4
28. Hat Ihr körperlicher Zustand oder Ihre medizinische Behandlung für Sie finanzielle Schwierigkeiten mit sich gebracht?	1	2	3	4

Bitte kreuzen Sie bei den folgenden Fragen die Zahl zwischen 1 und 7 an, die am besten auf Sie zutrifft

29. Wie würden Sie insgesamt Ihren Gesundheitszustand während der letzten Woche einschätzen?

1	2	3	4	5	6	7
sehr schlecht						ausgezeichnet

30. Wie würden Sie insgesamt Ihre Lebensqualität während der letzten Woche einschätzen?

1	2	3	4	5	6	7
sehr schlecht						ausgezeichnet

Bitte schauen Sie noch einmal, ob Sie alle Fragen beantwortet haben!

Vielen Dank für Ihre Teilnahme!

Code: ________________ Datum: ________________

EORTC QLQ - H&N35

Patienten berichten manchmal die nachfolgend beschriebenen Symptome oder Probleme. Bitte beschreiben Sie, wie stark Sie diese Symptome oder Probleme während der letzten Woche empfunden haben.

Während der letzten Woche:		Überhaupt nicht	Wenig	Mäßig	Sehr
31.	Hatten Sie Schmerzen im Mund ?	1	2	3	4
32.	Hatten Sie Schmerzen im Kiefer ?	1	2	3	4
33.	Hatten Sie wunde Stellen im Mund ?	1	2	3	4
34.	Hatten Sie Halsschmerzen ?	1	2	3	4
35.	Hatten Sie Probleme, Flüssiges zu schlucken ?	1	2	3	4
36.	Hatten Sie Probleme, weiche (pürrierte) Kost zu schlucken ?	1	2	3	4
37.	Hatten Sie Probleme, feste Kost zu schlucken ?	1	2	3	4
38.	Hatten Sie sich verschluckt ?	1	2	3	4
39.	Hatten Sie Probleme mit den Zähnen ?	1	2	3	4
40.	Hatten Sie Probleme, den Mund weit zu öffnen ?	1	2	3	4
41.	Hatten Sie einen trockenen Mund ?	1	2	3	4
42.	Hatten Sie klebrigen Speichel ?	1	2	3	4
43.	Hatten Sie Probleme mit Ihrem Geruchssinn ?	1	2	3	4
44.	Hatten Sie Probleme mit Ihrem Geschmackssinn ?	1	2	3	4
45.	Mußten Sie husten ?	1	2	3	4
46.	Waren Sie heiser ?	1	2	3	4
47.	Fühlten Sie sich krank ?	1	2	3	4
48.	Hat Sie Ihr Aussehen gestört ?	1	2	3	4

Während der letzten Woche:		Überhaupt nicht	Wenig	Mäßig	Sehr
49.	Hatten Sie Schwierigkeiten beim Essen ?	1	2	3	4
50.	Hatten Sie Schwierigkeiten, im Beisein Ihrer Familie zu essen ?	1	2	3	4
51.	Hatten Sie Schwierigkeiten , im Beisein anderer Leute zu essen ?	1	2	3	4
52.	Hatten Sie Schwierigkeiten, Ihre Mahlzeiten zu genießen?	1	2	3	4
53.	Hatten Sie Schwierigkeiten, mit anderen Leuten zu sprechen ?	1	2	3	4
54.	Hatten Sie Schwierigkeiten, am Telefon zu sprechen ?	1	2	3	4
55.	Hatten Sie Schwierigkeiten im Umgang mit Ihrer Familie ?	1	2	3	4
56.	Hatten Sie Schwierigkeiten im Umgang mit Ihren Freunden ?	1	2	3	4
57.	Hatten Sie Schwierigkeiten,unter die Leute zu gehen ?	1	2	3	4
58.	Hatten Sie Schwierigkeiten beim körperlichen Kontakt in der Familie oder mit Freunden ?	1	2	3	4
59.	Hatten Sie weniger Interesse an Sexualität ?	1	2	3	4
60.	Konnten Sie Sexualität weniger genießen ?	1	2	3	4

Während der letzten Woche:		Nein	Ja
61.	Haben Sie Schmerzmittel eingenommen ?	1	2
62.	Haben Sie Ihre Nahrung durch Zusatzstoffe ergänzt (außer Vitamine) ?	1	2
63.	Haben Sie eine Ernährungssonde verwendet ?	1	2
64.	Haben Sie an Gewicht abgenommen ?	1	2
65.	Haben Sie an Gewicht zugenommen ?	1	2

Bitte schauen Sie noch einmal, ob Sie alle Fragen beantwortet haben!

Vielen Dank für Ihre Teilnahme!

Code: ________________ Datum: ________________

GSLTPAQ

Wie oft üben Sie aktuell während einer typischen 7-Tage-Periode (eine Woche) im Durchschnitt die folgenden Aktivitäten in Ihrer Freizeit **mehr als 15 Minuten** aus? (Schreiben Sie in jede Zeile die entsprechende Zahl).

	**Häufigkeit pro Woche**	**Durchschnittliche Dauer pro Termin?**
**Anstrengende körperliche Aktivität** (erhöhte Anstrengung und Schwitzen) z. B. intensives Schwimmen, Jogging, Fußballspielen, Radsport	___ mal pro Woche	____ min
**Mäßige körperliche Aktivität** (kaum erhöhte Anstrengung und leichtes Schwitzen) z. B. schnelles Gehen, langsames Radfahren, langsames Schwimmen	___ mal pro Woche	____ min
**Leichte körperliche Aktivität** (keine erhöhte Anstrengung und kein Schwitzen) z. B. Golf, leichtes Gehen, Angeln	___ mal pro Woche	____ min

Score: ___________ ___________

Auswertung:
Summe aus (Häufigkeit anstrengende körperliche Aktivität x 9) + (Häufigkeit mäßige körperliche Aktivität x 5) + (Häufigkeit leichte körperliche Aktivität x 3)

Protokoll für die Funktionsdiagnostik im Rahmen der Studie

„Individuelles Heimtraining für Patienten mit Kopf-Halstumoren – eine multizentrische Studie zur Verbesserung der Lebensqualität"

Code: ____________________

Datum der Untersuchung: ________. ________. __________ Uhrzeit: ________: ________

- ☐ Eingangsuntersuchung
- ☐ Ausgangsuntersuchung
- ☐ Follow Up

Name des Untersuchers: ____

POMS (Profile of Mood States) – VAS

Im Folgenden finden Sie eine Liste von Wörtern, die verschiedene Stimmungen beschreiben. Bitte gehen Sie die Wörter der Liste nacheinander durch und machen Sie bei jedem Wort auf der vorgegebenen Linie ein Kreuz, das die **augenblickliche** Stärke Ihrer Stimmung am besten beschreibt.

Bitte beachten Sie dabei folgende Punkte: In der Liste sind mehrere Adjektive enthalten, die möglicherweise dieselbe oder eine ähnliche Stimmung beschreiben. Lassen Sie sich dadurch nicht verwirren, und geben Sie Ihre Antwort bei jedem Adjektiv unabhängig davon, wie Sie bei einem anderen Adjektiv geantwortet haben. Beurteilen Sie nur, wie Sie sich **augenblicklich** fühlen, nicht wie Sie sich im Allgemeinen oder gelegentlich fühlen. Wenn Ihnen die Antwort schwerfallen sollte, geben Sie die Antwort, die am ehesten zutrifft.

Geben Sie bitte bei jedem Wort ein Urteil ab und lassen Sie keines der Wörter aus.

		**überhaupt nicht** — **sehr stark**
01.	lustlos	|———————————|
02.	erschöpft	|———————————|
03.	müde	|———————————|
04.	abgeschlafft	|———————————|
05.	ermattet	|———————————|
06.	entkräftet	|———————————|
07.	träge	|———————————|

<u>Teil 1</u>: Bioimpedanzanalyse (BIA)

☐ BIA konnte nicht durchgeführt werden

Körpergröße: ________ m

Körpergewicht: ________ kg

Taillenumfang: ________ cm

Skelettmuskelmasse: _______ kg

Fettmasse: _______ kg oder Körperfettanteil: ______ %

<u>Teil 2</u>: aktives Bewegungsausmaß der Schultergelenke und der Halswirbelsäule

A) Schultergelenke

	Ante- / Retroversion	Ab- / Adduktion	Außen- / Innenrotation
**rechts**	**____/ 0 /____**	**____/ 0 /____ (max. 90°)**	**____/ 0 /____**
**links**	**____/ 0 /____**	**____/ 0 /____ (max. 90°)**	**____/ 0 /____**

Alternative Testung der Außen- / Innenrotation, falls der Pat duktion nicht oder nur unter Schmerzen erreicht:

rechts **____/ 0 /____**

links **____/ 0 /____**

Hat der Patient Schmerzen beim Bewegen des Armes?

rechter Arm ☐ nein ☐ ja, bei der ______________________________

linker Arm ☐ nein ☐ ja, bei der ______________________________

Fühlt sich der Patient im Bewegungsausmaß der Schultergelenke eingeschränkt?

rechter Arm ☐ nein ☐ ja, bei der ______________________________

linker Arm ☐ nein ☐ ja, bei der ______________________________

Sind Sie als Therapeut der Meinung, dass die Beweglichkeit der Schultergelenke eingeschränkt ist?

rechter Arm ☐ nein ☐ ja, bei der ____

linker Arm ☐ nein ☐ ja, bei der ____

B) Halswirbelsäule

**Flexion / Extension**	**Rechts- / Linksneigung**	**Rechts- / Linksrotation**
____/ 0 /____	____/ 0 /____	____/ 0 /____

Hat der Patient Schmerzen beim Bewegen des Kopfes?

☐ nein ☐ ja, bei der ____

Fühlt der Patient Einschränkungen beim Bewegen des Kopfes?

☐ nein ☐ ja, bei der ____

Sind Sie als Therapeut der Meinung, dass die Beweglichkeit des Kopfes eingeschränkt ist?

☐ nein ☐ ja, bei der ____

Teil 3: Kinn-Brustbein-Abstand (Kinnspitze – Incisura jugularis)

a) bei Inklination _______ cm

a) bei Reklination _______ cm

Teil 4: Interzahnabstand bei maximaler Mundöffnung

_______ cm (Schneidekantendistanz)

Teil 5: Rumpfflexibilität (Stand and reach Test)

_______ cm

Teil 6: Short Physical Performance Battery

Gesamtpunktzahl:

Teil 7: 6-Minuten-Gehtest

Zurückgelegte Strecke [m]: ____________

BORG-Skala: ____________

CR-10 Skala: ____________

Anmerkungen

____

____

____

____

____

____

____

____

____

____

____

____

____

____

____

BORG-Skala

Wie anstrengend war der 6-Minuten Gehtest für Sie?

Bitte beurteilen Sie das **Anstrengungsempfinden!**

**6**	
**7**	**Sehr, sehr leicht**
**8**	
**9**	**Sehr leicht**
**10**	
**11**	**Recht leicht**
**12**	
**13**	**Etwas anstrengender**
**14**	
**15**	**Anstrengend**
**16**	
**17**	**Sehr anstrengend**
**18**	
**19**	**Sehr, sehr anstrengend**
**20**	

CR-10 Skala – subjektive Bewertung des Belastungsschmerzes

Sie werden heute einen 6-Minuten-Gehtets durchführen. Die Skala vor Ihnen umfasst Zahlen von 0-10. Sie werden diese Skala verwenden, um den wahrgenommenen Belastungsschmerz in den Beinen während und nach der Belastung zu bewerten. Unter- oder überschätzen Sie das Ausmaß des empfundenen Belastungsschmerzes nicht, sondern versuchen Sie die Bewertung so ehrlich und objektiv wie möglich vorzunehmen. Die Zahlen auf der Skala beschreiben die Schmerzintensität von 0.5 (*„extrem schwach“*) bis 10 (*„extrem intensiv – nahezu unerträglich“*). Wenn Sie während der Belastung keine Schmerzen in den Beinen wahrnehmen, dann wählen Sie die „0“. Sollten Sie während der Belastung extrem starke Schmerzen in den Beinen verspüren, die nahezu unerträglich sind, dann sollten Sie die „10“ wählen. Bitte achten Sie darauf, dass Sie Ihre Bewertung nur auf den Belastungsschmerz in den Beinen beziehen und andere Wahrnehmungen (Lärm o. ä.) nicht in Ihrer Bewertung berücksichtigt werden. Ihre Bewertung sollte weder Ausdruck Ihres Ermüdungszustandes noch Ihrer Erleichterung nach Beendigung der Aufgabe sein.

Zusammenfassung:

(I) Bewerten Sie nur den empfundenen Belastungsschmerz in den Beinen;
(II) Ihre Bewertungen sollten so genau wie möglich sein;
(III) Unter- oder überschätzen Sie das Ausmaß des Belastungsschmerzes nicht, sondern bewerten Sie so ehrlich wie möglich.

Haben Sie noch Fragen?

CR-10 Skala – subjektive Bewertung des Belastungsschmerzes

0	überhaupt kein Schmerz
0.3	
0.5	extrem schwach<br>(gerade so spürbar)
1	sehr schwach
1.5	
2	schwach
2.5	
3.0	moderat
4	relativ stark
5	stark
6	
7	sehr stark
8	
9	
10	extrem intensiv<br>(nahezu unerträglich)
®	unerträglicher Schmerz

Trainingstagebuch

Code: ___________________ Datum Trainingsstart: ____________________

Wir empfehlen, das **individuell zusammengestellte Übungsprogramm** mind. an **3 Tagen pro Woche** mit einer Dauer von **15-30 min** zu absolvieren. Sie können die Übungszeit auch in kleine Einheiten, z. B. 2x10 min pro Tag aufteilen. Zusätzlich zum individuellen Programm sollten Sie versuchen 2-3mal pro Woche für ca. 30 min ein **Ausdauertraining** zu absolvieren, z. B. in Form von (Nordic-)Walking, Rad fahren, einem Spaziergang, Tanzen o. ä.

Um eine Überlastung durch das Training zu vermeiden, empfehlen wir zur Steuerung des Trainings die BORG-Skala. Die Borg-Skala von 6 bis 20 misst den **individuell empfundenen Grad der Anstrengung beim Kraft- und Ausdauertraining**.

Die optimale Anstrengung liegt zwischen den Werten **11 und 15**. Wird die sportliche Aktivität als "etwas anstrengend" empfunden, reicht dies aus, um positive Trainingseffekte zu erzielen.

Bitte beachten Sie, dass in bestimmten Situationen anstrengende körperliche Aktivitäten nicht durchgeführt werden sollten und ein **Training an diesen Tagen nicht möglich** ist.

- Bei Übelkeit bzw. Erbrechen
- Bei starken Schmerzen
- Bei Bewusstseinseinschränkungen und Verwirrtheit
- Bei Schwindel und Kreislaufbeschwerden
- Bei Fieber bzw. Temperatur > 38°C bzw. bei starkem Infekt

Bitte notieren Sie solche Zustände in Ihrem Trainingstagebuch!

Bitte notieren Sie **jeden Tag**, ob Sie trainiert haben. Machen Sie ein Kreuz bei den Inhalten, die Bestandteil des Trainings waren und tragen Sie dahinter die **Dauer Ihres Trainings** ein.

Wir wünschen Ihnen viel Spaß beim Training!

Woche 1						
**Tag**	**Training ja/nein**	**Individuelles Programm**	**Dauer [min]**	**Ausdauer [Inhalt]**	**Dauer [min]**	**Bemerkungen (z. B. Krankheit)**
**Mo**						
**Di**						
**Mi**						
**Do**						
**Fr**						
**Sa**						
**So**						

Woche 2						
**Tag**	**Training ja/nein**	**Individuelles Programm**	**Dauer [min]**	**Ausdauer [Inhalt]**	**Dauer [min]**	**Bemerkungen (z. B. Krankheit)**
**Mo**						
**Di**						
**Mi**						
**Do**						
**Fr**						
**Sa**						
**So**						

Woche 3						
**Tag**	**Training ja/nein**	**Individuelles Programm**	**Dauer [min]**	**Ausdauer [Inhalt]**	**Dauer [min]**	**Bemerkungen (z. B. Krankheit)**
**Mo**						
**Di**						
**Mi**						
**Do**						
**Fr**						
**Sa**						
**So**						

Woche 4						
Ta g	Training ja/nein	Individuelles Programm	Dauer [min]	Ausdauer [Inhalt]	Dauer [min]	Bemerkungen (z. B. Krankheit)
Mo						
Di						
Mi						
Do						
Fr						
Sa						
So						

Woche 5						
Tag	Training ja/nein	Individuelles Programm	Dauer [min]	Ausdauer [Inhalt]	Dauer [min]	Bemerkungen (z. B. Krankheit)
Mo						
Di						
Mi						
Do						
Fr						
Sa						
So						

Woche 6						
Tag	Training ja/nein	Individuelles Programm	Dauer [min]	Ausdauer [Inhalt]	Dauer [min]	Bemerkungen (z. B. Krankheit)
Mo						
Di						
Mi						
Do						
Fr						
Sa						
So						

Woche 7						
Tag	Training ja/nein	Individuelles Programm	Dauer [min]	Ausdauer [Inhalt]	Dauer [min]	Bemerkungen (z. B. Krankheit)
Mo						
Di						
Mi						
Do						
Fr						
Sa						
So						

Woche 8						
Tag	Training ja/nein	Individuelles Programm	Dauer [min]	Ausdauer [Inhalt]	Dauer [min]	Bemerkungen (z. B. Krankheit)
Mo						
Di						
Mi						
Do						
Fr						
Sa						
So						

Woche 9						
Tag	Training ja/nein	Individuelles Programm	Dauer [min]	Ausdauer [Inhalt]	Dauer [min]	Bemerkungen (z. B. Krankheit)
Mo						
Di						
Mi						
Do						
Fr						
Sa						
So						

Woche 10						
**Tag**	**Training ja/nein**	**Individuelles Programm**	**Dauer [min]**	**Ausdauer [Inhalt]**	**Dauer [min]**	**Bemerkungen (z. B. Krankheit)**
**Mo**						
**Di**						
**Mi**						
**Do**						
**Fr**						
**Sa**						
**So**						

Woche 11						
**Tag**	**Training ja/nein**	**Individuelles Programm**	**Dauer [min]**	**Ausdauer [Inhalt]**	**Dauer [min]**	**Bemerkungen (z. B. Krankheit)**
**Mo**						
**Di**						
**Mi**						
**Do**						
**Fr**						
**Sa**						
**So**						

Woche 12						
**Tag**	**Training ja/nein**	**Individuelles Programm**	**Dauer [min]**	**Ausdauer [Inhalt]**	**Dauer [min]**	**Bemerkungen (z. B. Krankheit)**
**Mo**						
**Di**						
**Mi**						
**Do**						
**Fr**						
**Sa**						
**So**						

Bitte nehmen Sie das ausgefüllte Trainingstagebuch mit zur Ausgangsuntersuchung!

Vielen Dank, dass Sie an der Studie teilnehmen!

Telefonleitfaden und -protokoll zur HeiKo-Studie:

„Individuelles Heimtraining für Patienten mit Kopf-Halstumoren – eine multizentrische Studie zur Verbesserung der Lebensqualität“

Während der 12-wöchigen Trainingsintervention im Home-based Setting sollen die Studienteilnehmer **einmal wöchentlich** angerufen und der **Status quo** erfragt und protokolliert werden. Das Telefonat dient primär der **Motivation des Patienten**. Gleichzeitig sollen Trainingsumfänge, unerwünschte Ereignisse (Adverse Events) während des Trainings, das Wohlbefinden und die aktuelle Motivation der Patienten erfasst werden. Bei Motivationsmangel sollen mögliche Gründe erfragt werden. Haben die Probanden Fragen zum Training, sollen diese bestmöglich beantwortet werden.

Telefonleitfaden

1. Erfragen des **aktuellen Wohlbefindens** auf einer Skala von 0 (sehr schlecht) – 10 (sehr gut)

0 sehr schlecht | 1 | 2 | 3 | 4 | 5 „mittelmäßig“ | 6 | 7 | 8 | 9 | 10 sehr gut

2. Erfragen der **Trainingshäufigkeit** und **-dauer** in der letzten bzw. aktuellen Woche
   - Loben für jedes Training, unabhängig wie oft und lange; auch wenn nicht trainiert wurde (z. B. wegen Krankheit) aufmuntern, Zuspruch

3. Erfassung von **Adverse Events** (z. B. Übelkeit, Schwindel) beim Training
   - falls ja, fragen was und wobei (Trainingsprogramm oder Ausdauertraining)
   - ggf. Hinweise zum Training geben (Inhalt, Intensität, Dauer)

4. Erfragen der **Motivation** auf einer Skala von 1 (überhaupt nicht) – 4 (sehr motiviert)

1 überhaupt nicht | 2 wenig | 3 mäßig | 4 sehr

   - ggf. Gründe erfragen und Hinweise geben

5. Fragen der Studienteilnehmer zum Training und **Sonstiges (evtl. Abbruchsgrund)**

Ausfüllhilfe Protokoll:

Woche	Datum	Wohlbefinden 0-10	Trainings-häufigkeit	Trainings-dauer	Adverse Events	Motivation 1-4	Sonstiges (Abbruchsgrund)
1	22.02.22	7	3x	15-25`	Übelkeit beim Tanzen	3	kommt gut zurecht

Telefonprotokoll **Patient:** ______________________________ **Code:** ____________________________

Vor Versand Namen schwärzen!

Trainingsstart: _____. _____. _________ **KW:** _________

Telefonat: bevorzugte/r Tag/e: _________________________ bevorzugte Zeit: __________________________

Woche	Datum	Wohlbefinden 0-10	Trainings-häufigkeit	Trainings-dauer	Adverse Events	Motivation 1-4	Sonstiges (evtl. Abbruchsgrund)
1							
2							
3							
4							
5							
6							
7							
8							
9							
10							
11							
12							

Fragebogen 2 zur Studie:
„Individuelles Heimtraining für Patienten mit Kopf-Halstumoren – eine multizentrische Studie zur Verbesserung der Lebensqualität"

Sehr geehrte Studienteilnehmerin, sehr geehrter Studienteilnehmer,

wir freuen uns, dass Sie an der Trainingsstudie teilgenommen haben.
Bitte füllen Sie für die Auswertung den vorliegenden Fragebogen vollständig aus. Kreuzen Sie jeweils die Antwort an, die auf Sie zutrifft bzw. die Ihrer Vorstellung am ehesten entspricht. Es gibt keine „richtigen" oder „falschen" Antworten. Markieren Sie die gültigen bzw. korrigierten Antworten wie folgt: gültige Antwort: ☒; bei Korrekturen die ungültige Antwort: ~~☒~~.

Wir versichern Ihnen, dass die Daten vertraulich und nur für wissenschaftliche Zwecke verwendet werden. Die Rückverfolgung zu Ihrer Person ist ausgeschlossen. Zu diesem Zweck haben wir einen Code generiert, über den die Zusammenführung Ihrer Daten pseudonymisiert über die verschiedenen Erhebungszeitpunkte erfolgt.

Code: ___________________

Datum: __________________

1. Haben Ihnen das individuelle Heimtrainingsprogramm und die Trainingsempfehlungen geholfen, sich in folgenden Bereichen zu verbessern?

	**nein**	**etwas**	**ziemlich**	**sehr**
Allgemeine Leistungsfähigkeit	☐1	☐2	☐3	☐4
Körperliches Wohlbefinden	☐1	☐2	☐3	☐4
Kraftfähigkeiten	☐1	☐2	☐3	☐4
Ausdauerleistungsfähigkeit	☐1	☐2	☐3	☐4
Beweglichkeit	☐1	☐2	☐3	☐4
Koordination	☐1	☐2	☐3	☐4
Stressabbau	☐1	☐2	☐3	☐4
Körperwahrnehmung	☐1	☐2	☐3	☐4
Selbstwertgefühl	☐1	☐2	☐3	☐4
allgemeine Stimmung	☐1	☐2	☐3	☐4
______________________________	☐1	☐2	☐3	☐4

2. Wie schwer war es im Durchschnitt für Sie, sich für das Training zu motivieren?

☐1 überhaupt nicht ☐2 wenig ☐3 mäßig ☐4 sehr

3. Hatten die wöchentlichen Telefonate einen positiven Einfluss auf Ihre Motivation?

☐1 nein ☐2 etwas ☐3 ziemlich ☐4 sehr

4. Haben Sie Ihr Training zusammen mit anderen (z. B. Familienangehörigen, Freunden) durchgeführt?

☐0 Nein, ich habe immer allein trainiert.

☐1 Ja.

☐2 Zum Teil.

5. Glauben Sie, dass Sie das Training auch nach der Studie aufrechterhalten werden?

☐1 Ja, ich werde das Training beibehalten.

☐2 Ich weiß nicht, bin mir nicht ganz sicher.

☐3 Nein. Warum nicht: ____

6. Waren die Informationen, die Sie zum Trainingsprogramm erhalten haben, ausreichend?

☐1 Ja.

☐2 Nein. Ich hätte mir mehr Informationen gewünscht, z. B. zu ____________________

____

____

7. Welche Materialien haben Sie für Ihr Training genutzt? (Mehrfachnennung möglich)

☐ das Übungshandbuch

☐ die individuelle Übungszusammenstellung auf der DVD

☐ die bereitgestellten Kleingeräte

8. Sofern Sie eine *intensive sportliche Aktivität* (d.h. für jeweils **20 min an mindestens 3 Tagen pro Woche**) bereits vor der Studienteilnahme regelmäßig ausgeübt haben, haben Sie diese während der Studienteilnahme weitergeführt?

☐1 Ja.

☐2 Zeitweise ja.

☐3 Nein. Warum nicht: ____

9. Haben Sie weitere Hinweise / Anmerkungen für uns, wie wir Patienten mit Kopf-Hals-Tumoren für ein Training motivieren bzw. unterstützen können?

____

____

____

____

____

____

Aufgaben des Therapeuten und Übersicht der Dokumente für die Eingangs-, Ausgangs- und Follow Up-Untersuchungen im Rahmen der HeiKo-Studie:

„Individuelles Heimtraining für Patienten mit Kopf-Halstumoren –
eine multizentrische Studie zur Verbesserung der Lebensqualität“

	**Eingangs-untersuchung**	**Ausgangs-untersuchung**	**Follow Up**
**vom Therapeuten durchzuführen:**			
**Pseudonymisierung** der rekrutierten Patienten (Einwilligung muss vorliegen)<br>(Klarliste führen und getrennt von anderen Unterlagen aufbewahren)<br>Code: Stadt_Nr. fortlaufend ab 1<br>z.B. Rostock_01			
Festlegung der **Untersuchungstermine** (ggf. Erinnerung)	x	x	x
**Code** (vor Ausgabe) auf allen Dokumenten notieren	x	x	x
Kontrolle der ausgefüllten **Patientenfragebögen** (s.u.)	x	x	x
Einholung der **krankheitsspezifischen Angaben** vom Ärzteteam	x		
Durchführung **Funktionsdiagnostik** entsprechend dem Protokoll	x	x	x
**Datenweiterleitung** an die Studienzentrale der Universitätsmedizin Rostock und an die Studienleiterin	x	x	x
**Einweisung** des Patienten in das **Training** / Nutzung der Unterlagen	nach Zusendung der individuellen Übungszusammenstellung		
Wöchentliches **Telefonat** und Führen des **Telefonprotokolls**	Während der Intervention: einmal wöchentlich Status quo erfragen und protokollieren		
**vom Patienten auszufüllen:**			
Eingangsfragebogen (personenspezifische Daten)	x		
EORTC-QLQ-30	x	x	x
EORTC-QLQ-H&N35	x	x	x
GSLTPAQ	x	x	x
Ausgangsfragebogen		x	
Trainingstagebuch	Während der Intervention: täglich das Training notieren<br>Abgabe des Trainingstagebuches bei der Ausgangsuntersuchung		

Kontaktdaten

xxx

Patienteninformation zur Studie:

„Individuelles Heimtraining für Patienten mit Kopf-Halstumoren –
eine multizentrische Studie zur Verbesserung der Lebensqualität" **(HeiKo-Studie)**

Sehr geehrte Patientin, sehr geehrter Patient,

Sie haben mit Ihrer Ärztin/Ihrem Arzt bzw. mit der/dem Untersuchungsleiter/in besprochen, dass Sie an der bevorstehenden Studie teilnehmen. Die Ärztin/der Arzt bzw. die/der Untersuchungsleiter/in hat Sie über den Ablauf der Studie informiert und lädt Sie ein, an dieser teilzunehmen. Bevor sie sich entscheiden, ist es für Sie wichtig zu verstehen, was diese Studie beinhaltet und warum diese durchgeführt wird. Bitte nehmen Sie sich genügend Zeit, um diese Information sorgfältig zu lesen und zögern Sie nicht, evtl. verbleibende Fragen zu besprechen.

Die Teilnahme an dieser Studie ist freiwillig und kann jederzeit ohne Angabe von Gründen durch Sie beendet werden.

Bitte unterschreiben Sie die Einwilligungserklärung nur,

- wenn Sie Art und Ablauf der Studie vollständig verstanden haben
- wenn Sie bereit sind, der Teilnahme zuzustimmen
- wenn Sie sich über Ihre Rechte als Teilnehmer an dieser Studie im Klaren sind.

Diese Studie, die Patienteninformation, Einwilligungs- und Datenschutzerklärung wurden von der zuständigen Ethikkommission geprüft und genehmigt.

Was sind die Ziele dieser Studie?

Ziel dieser Studie ist es, den Ansatz des individuellen Heimtrainings bei Patienten mit Kopf-Halstumoren in einer multizentrischen Studie (Teilnahme mehrerer Kliniken und Praxen) bezüglich seiner Machbarkeit und seiner Effekte auf verschiedenen Grade der Lebensqualität und Funktionalität zu untersuchen. Dafür werden den teilnehmenden Patienten individuelle Trainingsprogramme zur Verfügung gestellt. Bei der Erstellung der Trainingsprogramme werden die individuellen krankheits- und therapiebedingten Nebenwirkungen berücksichtigt.

Ablauf der Befragungen / Untersuchungen und Inhalte des Trainingsprogrammes

1. Fragebögen

Vor Trainingsbeginn werden mittels Fragebogen Angaben zur Person und zur Sportvergangenheit eingeholt. Die Lebensqualität wird mittels zweier Fragebögen (EORTC-QLQ-30, EORTC-H&N35) erhoben. Zusätzlich wird die körperliche Aktivität erfasst. Die Fragebogenerhebung wird nach dem 12-wöchigen Trainingsprogramm und nach weiteren 12 Wochen (Follow Up) wiederholt. Nach Absolvierung des Trainingsprogrammes werden zusätzlich Angaben zur Zufriedenheit und Motivation eingeholt.

Während des 12-wöchigen Trainingsprogramms führen die Patientin ein Trainingstagebuch.

2. Untersuchungen

Zur objektiven Beurteilung der physischen Leistungsfähigkeit, der Funktionalität und der Körperzusammensetzung kommen verschiedene Untersuchungen zum Einsatz. Zu diesen zählen:

a) Messung des Interzahnabstandes bei maximaler Mundöffnung
b) Messung des Bewegungsausmaßes

   Mit Hilfe eines Goniometers (manuelles Winkelmessgerät) wird das aktive Bewegungsausmaß der Schultergelenke und der Halswirbelsäule erfasst. Der Patient gibt zudem an, ob er Schmerzen bei den Bewegungen hat und sich in den Bewegungen eingeschränkt fühlt.
c) Messung der Flexibilität des Rumpfes mittels „stand and reach Test“ (Rumpftiefbeuge mit gestreckten Knien)
d) Short Physical Performance Battery

   Diese Testbatterie umfasst insgesamt drei Tests. Als erstes werden Gleichgewichtstests durchgeführt. Im Anschluss wird die Gehgeschwindigkeit über eine Strecke von 4 m bestimmt. Als letztes wird die Zeit gemessen, die der Teilnehmer benötigt um 5-mal nacheinander von einem Stuhl aufzustehen und sich wieder hinzusetzen.
e) 6-Minuten-Gehtest

   Die Patienten erhalten die Aufgabe in 6 Minuten eine möglichst weite Strecke auf einem steigungslosen Rundkurs (oder Gang) zu schaffen. Laufen, Tempoänderungen und Pausen sind dabei erlaubt. Gemessen wird die Wegstrecke in Metern. Die wahrgenommene Belastung bzw. der wahrgenommene Belastungsschmerz wird mittels zweier Skalen beurteilt.

Die Gesamtdauer der Untersuchungen beträgt zirka 30 min. Die Tests werden vor und nach dem 12-wöchigem Trainingsprogramm sowie 12 Wochen nach Beendigung des Trainingsprogramms (Follow Up) durchgeführt.

Alle erhobenen Daten werden anonymisiert verwendet, so dass kein offensichtlicher Rückschluss auf eine Person möglich ist.

3. Trainingsprogramm

Das Training erfolgt im Home-based Setting (Heimtrainingsprogramm) über einen Zeitraum von 12 Wochen. Die individuelle Übungszusammenstellung erfolgt durch Sportwissenschaftler und/oder Physiotherapeuten auf Grundlage der Ergebnisse der Eingangsdiagnostik. Entsprechend den individuellen Bedürfnissen/Einschränkungen werden Übungen zur Mobilisation, Koordination, Kräftigung und Dehnung zusammengestellt. Zudem werden Empfehlungen zum Ausdauertraining, zum Trainingsumfang und zur Trainingsintensität gegeben. Alle Übungen, die vorgeschlagen werden, finden sich im „Übungshandbuch für Patienten mit Mund-, Kiefer-, Gesichts- und Halstumoren", welches die Studienteilnehmer erhalten. Zusätzlich werden auf Wunsch die ausgewählten Übungen als Video auf einen Datenträger (DVD/Stick) gespeichert, den der Patient ebenfalls erhält. Materialen wie Gummibänder und Gymnastikbälle, die für die Übungen benötigt werden, werden dem Patienten kostenlos zur Verfügung gestellt. Während des 12-wöchigen Heimtrainingsprogrammes wird der Patient einmal wöchentlich von einem Therapeuten angerufen und der Status quo (Trainingshäufigkeit, -umfang, Befinden, Motivation) erfragt. Eventuell auftretende Fragen zum Training oder zu den Übungen können dabei besprochen werden. Während der 12 Wochen protokolliert der Teilnehmer sein durchgeführtes Training in einem Trainingstagebuch.

Mögliche Risiken und Nachteile

Auch wenn alle oben aufgeführten Untersuchungen und das Training nicht-invasiv sind, können auf Grund der ungewohnten Belastung, speziell beim Ausdauertraining, Zwischenfälle (z. B. Übelkeit, Erbrechen, muskuläre Beschwerden, Kreislaufbeschwerden) auftreten. Generell wird das Training onkologischer Patienten im Rahmen von klinischen Studien und in der Routineversorgung als gut durchführbar und sicher bewertet, da die Wahrscheinlichkeit für das Auftreten eines „unerwünschten Ereignisses" als niedrig eingeschätzt werden kann (laut aktueller Studienlage). Um Verletzungen und Unfälle zu vermeiden, ist es zwingend notwendig den Anweisungen der Therapeuten unbedingt Folge zu leisten. Zudem sind alle Fragen in Bezug auf Krankheiten, Gesundheitszustand und Medikamenteneinnahme wahrheitsgemäß zu beantworten. Auch bei regelgerechter Übungsdurchführung kann es zu unvorhergesehenen Ereignissen und Zwischenfällen kommen.

Hinweis: Auf dem Weg zu den Untersuchungsterminen und für das Training besteht kein gesonderter Versicherungsschutz (hier greift im Fall eines (Wege-)Unfalls Ihre persönliche Unfallversicherung.)

Nutzen der Untersuchungen und Trainingsintervention

Die hierbei gewonnenen Daten liefern detaillierte Erkenntnisse über

a) die Abbruchquote einer Heimtrainingsprogrammen bei Patienten mit Mund-, Kiefer- Gesichts- und Halstumoren in der Nachsorge.

b) die Auswirkungen eines 12-wöchigen Heimtrainingsprogramms auf die Lebensqualität und Funktionalität, Körperzusammensetzung und Alltagsaktivität von Patienten mit Mund-, Kiefer-Gesichts- und Halstumoren in der Nachsorge.
c) die Zusammenhänge zwischen Trainingsumfang, Lebensqualität, Funktionalität, Körperzusammensetzung und Alltagsaktivität.

Vorzeitige Beendigung der Untersuchung

Sie können jeder Zeit, auch ohne Angabe von Gründen, Ihre Teilnahme ablehnen oder widerrufen und aus der Untersuchung ausscheiden, ohne dass Ihnen dadurch Nachteile entstehen.
Ihr Arzt/Therapeut wird Sie umgehend über alle neuen Erkenntnisse informieren, die in Bezug zu dieser Untersuchung bekannt werden und für Sie wichtig sein könnten.

Verwendung der gesammelten Daten

Sofern gesetzlich nicht etwas Anderes vorgesehen ist, haben nur der Untersuchungsleiter und dessen Mitarbeiter Zugang zu den vertraulichen Daten, in denen Sie namentlich genannt werden. Diese Personen unterliegen der Schweigepflicht. Die Daten werden pseudonymisiert online über eine verschlüsselte Verbindung an die Studienzentrale der Universitätsmedizin Rostock versendet. Die Weitergabe der Daten erfolgt ausschließlich zu statistischen Zwecken und Sie werden ausnahmslos nicht namentlich darin genannt. Auch in etwaigen Veröffentlichungen der Daten dieser Studie werden Sie nicht namentlich genannt.

Kosten

Für die Teilnahme an dieser Untersuchung wird keine finanzielle Vergütung gezahlt.

Weitere Fragen

Für weitere Fragen im Zusammenhang mit dieser Untersuchung stehen Ihnen die Studienleiterin und ihre Mitarbeiter/innen gern zur Verfügung. Auch Fragen, die Ihre Rechte als Teilnehmer/in an dieser Studie betreffen, werden Ihnen gerne beantwortet.

Studienleiterin:
Dr. phil. Sabine Felser
xxx

Ansprechpartner vor Ort:
Name des Prüfers oder verantwortlichen Therapeuten
Adresse
Telefonnummer

Einwilligungserklärung über die Teilnahme an der Studie: „Individuelles Heimtraining für Patienten mit Kopf-Halstumoren – eine multizentrische Studie zur Verbesserung der Lebensqualität“ (HeiKo-Studie)

bitte ankreuzen

- Ich bin in einem persönlichen Gespräch ausführlich und verständlich über das Wesen und die Bedeutung dieser multizentrischen Interventionsstudie aufgeklärt worden. Alle Fragen, die ich im Zusammenhang mit dieser Studie gestellt habe, sind zu meiner Zufriedenheit beantwortet worden. Der Arzt/Therapeut hat mir erklärt, dass ich mich jederzeit an ihn wenden kann, wenn sich für mich neue Fragen ergeben. Eine Kopie der Probanden-information und der Einwilligungserklärung habe ich erhalten.

  ☐ ja

  ☐ nein

- Ich willige in die Teilnahme an der Home-based Trainingsintervention ein und bin damit einverstanden, dass die im Rahmen der Studie bei mir erhobenen (Krankheits-)Daten aufgezeichnet und daran gewonnene Ergebnisse in pseudo-nymisierter Form ausgewertet werden.

  ☐ ja

  ☐ nein

Alle von mir gemachten Angaben und die Untersuchungsergebnisse unterliegen der ärztlichen Schweigepflicht. Sie werden für die Auswertung nur in pseudonymisierter Form verwendet.
Die Einwilligung kann ich jederzeit und ohne Angabe von Gründen widerrufen, ohne dass mir daraus Nachteile entstehen.

________________________________	______________
Name, Vorname Studienteilnehmer	Geburtsdatum
________________________________	________________________________
Ort, Datum<br>*(vom Studienteilnehmer einzutragen)*	Unterschrift des Studienteilnehmers
________________________________	________________________________
Ort, Datum	Unterschrift Arzt/Therapeut

Datenschutzerklärung zur Studie „Individuelles Heimtraining für Patienten mit Kopf-Halstumoren – eine multizentrische Studie zur Verbesserung der Lebensqualität“ (HeiKo-Studie)

Nachfolgend möchten wir Sie umfassend darüber informieren, was mit Ihren persönlichen Daten im Rahmen der HeiKo-Studie geschieht und wie mit ihnen umgegangen wird.

Wer ist verantwortlich für die Datenverarbeitung und -speicherung? An wen kann ich mich bei Fragen zum Datenschutz wenden?

Die HeiKo-Studie wird multizentrisch im Rahmen der Ostdeutschen Studiengruppe Hämatologie und Onkologie (OSHO e. V.) durchgeführt. Sponsor ist die Universitätsmedizin Rostock. Die Universitätsmedizin sowie alle teilnehmenden Zentren/Kliniken sind damit für den Schutz Ihrer personenbezogenen Daten verantwortlich (Art. 26 DSGVO).

Der lokale, für die Studie zuständige Arzt und Therapeut sind dafür verantwortlich, dass Ihre Daten richtig und ordnungsgemäß erhoben und in entsprechende Datenblätter eingetragen sowie gepflegt werden (Erhebungs- und Pflegeabschnitt).

Die Universitätsmedizin Rostock ist dabei verantwortlich, dass alle Daten verfügbar bleiben und die Daten sicher aufgehoben werden (Verfügbarkeitsabschnitt).

Im Rahmen der datenschutzrechtlichen Verantwortlichkeit ist die Universitätsmedizin Rostock für die Verarbeitung der personenbezogenen Daten im *Erhebungs- und Pflegeabschnitt* und für die Verarbeitung der personenbezogenen Daten im *Verfügbarkeitsabschnitt* zuständig.

Sie macht den betroffenen Personen die gemäß Art. 13 und 14 DSGVO erforderlichen Informationen in präziser, transparenter, verständlicher und leicht zugänglicher Form in einer klaren und einfachen Sprache unentgeltlich zugänglich.

<u>Verantwortliche Einrichtung</u>

Universitätsmedizin Rostock - rechtsfähige Teilkörperschaft der Universität Rostock
xxx
18057 Rostock

Ihr Ansprechpartner zur HeiKo-Studie
Zentrum für Innere Medizin, Medizinische Klinik III - Hämatologie, Onkologie, Palliativmedizin
Dr. phil. Sabine Felser
Dr. med. Christina Große-Thie,
xxxx

Name des Prüfzentrums
Straße, Hausnr.
PLZ, Ort
Internetauftritt

Ihr Ansprechpartner zur HeiKo-Studie in xxx
Bitte ergänzen (s.o.)
Bitte ergänzen (s.o.)
E-Mail:
Tel.: Fax:

Bei Fragen zum Datenschutz stehen Ihnen zusätzlich die Datenschutzbeauftragten der Einrichtungen zur Verfügung:

Datenschutzbeauftragter der Universitätsmedizin Rostock	Datenschutzbeauftragter [des Prüfzentrums]
Der Datenschutzbeauftragte<br>xxx	

Möchten Sie von Ihrem Beschwerderecht über rechtswidrige Datenverarbeitungen Gebrauch machen, wenden Sie sich bitte an die zuständige Aufsichtsbehörde:

Datenschutzaufsichtsbehörde für die Universitätsmedizin Rostock	Datenschutzaufsichtsbehörde für [das Prüfzentrum]
Der Landesbeauftragte für Datenschutz und Informationsfreiheit M-V<br>xxx	

Welche Daten werden erhoben und zu welchem Zweck? Auf welcher Grundlage erfolgt die Datenverarbeitung?

Zum einen werden allgemeine Angaben zu Ihrer Person (wie Alter und Geschlecht) erfasst. Zum anderen werden aber auch besonders sensible und schützenswerte Informationen zu Ihrem Gesundheitszustand benötigt. Diese Informationen werden nachfolgend als **Gesundheitsdaten** zusammengefasst. Die Gesundheitsdaten der HeiKo-Studie setzen sich insbesondere aus folgenden Angaben zu Ihrer Person zusammen:

- Diagnose(jahr)
- Daten zu Ihrer Behandlung
- Daten zu Ihrer Sporthistorie
- Daten der Leistungsdiagnostik
- Daten der Trainingstherapie (inkl. Erfassung unerwünschter Zwischenfälle)

Der Zweck der Weiterverwendung und somit Verarbeitung Ihrer personenbezogenen Daten, insbesondere **Gesundheitsdaten**, ist die Entwicklung und Umsetzung individueller Heimtrainingsprogramme, die sich positiv auf die Lebensqualität von Krebspatienten mit Tumoren im Kopf-Halsbereich auswirken.

Die Datenverarbeitung erfolgt auf Grundlage Ihrer Einwilligung gem. Art. 9 Abs. 2 lit. a DSGVO und zu jeder Zeit nur im erforderlichen Umfang.

Wer erhebt meine Daten und werden sie auch weitergegeben oder veröffentlicht? Wie werden meine Daten geschützt?

Während der Studie werden medizinische Befunde und persönliche Informationen von Ihnen durch den Arzt und den Therapeuten erhoben. Danach werden Ihre Daten einer sogenannten Pseudonymisierung unterzogen. Dies dient dem Schutz Ihrer Person, Ihrer Persönlichkeitsrechte und Ihrer Daten. Der Vorgang der Pseudonymisierung ist eine Art Verschlüsselung Ihrer Daten. Dabei werden alle persönlichen Identifikationsmerkmale (wie z. B. Name, Geburtsdatum, Anschrift) durch eine Nummer (einen Code) ersetzt. Nur der für diese Studie zuständige Arzt und Therapeut verfügt über eine Zuordnungsliste, mit deren Hilfe er den Code wieder entschlüsseln und die pseudonymisierten Daten zu Ihrer Person wieder zuordnen kann. Eine Entschlüsselung erfolgt nur dann, wenn es erforderlich ist bzw. ein Gesetz dies erlaubt. Somit ist für andere Einrichtungen nicht mehr nachzuvollziehen, zu welcher Person der Datensatz gehört.

Diese personenbezogenen Daten werden in pseudonymisierter (verschlüsselter) Form für die wissenschaftliche Auswertung der Studie an die Leiterin und die stellvertretende Leiterin der Studie weitergegeben.

Nachfolgend erhalten Sie eine Übersicht der möglichen Datenempfänger bei der Teilnahme an der HeiKo-Studie:

Empfänger	Art der Datenverarbeitung	Hinweise	Grundlage
**Universitätsmedizin Rostock** studienzuständige Arzt / Therapeut	Erhebung, Speicherung, Übermittlung zur wissenschaftlichen Auswertung	Hauptverantwortlicher und Auftraggeber für die Erhebung, Speicherung und Verarbeitung personenbezogener Daten	Ihre Einwilligung
**Universitätsmedizin Rostock** Leiterin und stellvertretende Leiterin der Studie	Speicherung und Verarbeitung, Übermittlung zur wissenschaftlichen Auswertung	Hauptansprechpartner für Anfragen und Auskünfte (Erstkontakt)	Ihre Einwilligung

Eine Veröffentlichung Ihrer Daten findet nicht statt. Alle beteiligten Institutionen und Personen sind per Gesetz dazu verpflichtet, Ihre Daten und Persönlichkeitsrechte zu achten und zu schützen. Dies bedeutet, dass alle Institutionen und Personen, welche Ihre Daten verarbeiten, sicherstellen müssen, dass die Daten vor Missbrauch und unbefugtem Zugriff geschützt sind. Weiterhin wird jeder beteiligte Partner nur jeweils diejenigen Daten verarbeiten und speichern, die für den eigentlichen Zweck erforderlich sind. Wenn personenbezogene Daten übermittelt werden, geschieht dies stets unter Einhaltung der gültigen Datenschutzgesetze. Es werden bei allen Datenübermittlungen zusätzliche technische Sicherheitsmaßnahmen eingesetzt.

Wo und wie lange werden meine Daten gespeichert?

Die im Rahmen der HeiKo-Studie erhobenen Daten werden zunächst pseudonymisiert und für die Dauer von 15 Jahren gespeichert. Die medizinischen Daten, die im Rahmen Ihrer allgemeinen Behandlung erhoben wurden, unterliegen gesetzlichen Aufbewahrungsfristen von bis zu 30 Jahren. Nähere Auskünfte dazu erhalten Sie von Ihrem behandelnden Arzt.

Welche Datenschutzrechte habe ich bei einer Teilnahme an der HeiKo-Studie und wie kann ich sie wahrnehmen?

Gemäß den geltenden Datenschutzbestimmungen steht Ihnen ein Beschwerderecht über rechtswidrige Datenverarbeitungen bei der zuständigen Aufsichtsbehörde zu. Daneben verfügen Sei selbst grundsätzlich über ein Recht auf *Auskunft* über gespeicherte Daten, *Berichtigung* unzutreffender Daten, *Löschung* von Daten, *Einschränkung der Verarbeitung* von Daten, *Widerspruch* gegen unzumutbare Datenverarbeitung und über ein Recht auf *Datenübertragbarkeit* der zu Ihrer Person gespeicherten Daten. Ebenso haben Sie das Recht Ihre *abgegebene Einwilligung zu widerrufen*.

Diese Rechte können Sie jederzeit gegenüber der verantwortlichen Einrichtungen ausüben. Wir bitten Sie jedoch sich stets **zuerst an Ihren Arzt oder Therapeuten** zu wenden, da diese Sie am besten und schnellsten bei Ihrem Anliegen unterstützen können. Es wird dann im Einzelfall geprüft, inwieweit Ihrem Begehren nachgegangen werden kann.

Der Antrag auf Auskunft kann formlos erfolgen. Wir empfehlen jedoch, den **Antrag schriftlich** zu stellen, zu datieren und konkrete Auskunftswünsche zu benennen. Unter Umständen müssen Sie Ihre Identität sowie Ihren Anspruch auf Auskunftserteilung erneut nachweisen (z. B. durch Vorlage Ihrer unterschriebenen Einwilligungserklärung zur Registerteilnahme; Personaldokument).

Hinsichtlich der von bestimmten Datenempfängern (z. B. Aufsichts- und Zulassungsbehörden) gespeicherten Daten bestehen im Rahmen der gültigen Gesetze nur eingeschränkte oder gar keine Auskunftsansprüche Ihrerseits.

Ich erkläre mich damit einverstanden, dass die Universitätsmedizin Rostock, wie in der Information beschrieben, personenbezogene Daten von mir erhebt und speichert.

Ich bin darüber aufgeklärt worden, dass ich meine Einwilligung gegenüber der Universitätsmedizin Rostock ohne Angabe von Gründen jederzeit widerrufen kann.

____

Name des Probanden in Druckbuchstaben

__________________________ __________________________________

Ort, Datum
(vom Probanden einzutragen)

Unterschrift des Probanden
