# Supplementary material for: Feasibility of individualized home exercise programs for patients with head and neck cancer–study protocol and first results of a multicentre single-arm intervention trial (OSHO #94)
Source: PLoS One. 2024 Aug 22;19(8):e0301304. doi: 10.1371/journal.pone.0301304 (PMC11341025; doi:10.1371/journal.pone.0301304)
Supplement: S2 File — (PDF) [file pone.0301304.s004.pdf]

Individual home exercise for patients with head and neck tumours - A multicentre study to improve quality of life

HeiKo-Study (OSHO #94)

Version 1 from 30/09/2020

**Sponsor:**	Prof. Dr. Christian Junghanß<br>xxx
**Study leader:**	Dr. phil. Sabine Felser<br>xxx
**Deputy leader of study:**	Dr. med. Christina Große-Thie<br>xxx
**Data management:**	Head: Dr. med. Brigitte Kragl<br>xxx

The information in this protocol is strictly confidential. It is intended solely for the information of the sponsor, the investigators, the study staff, the ethics committee, the authorities and the patients. This protocol may not be passed on to third parties without the consent of the sponsor or the project leader.

I. Signatures

Dr. phil. Sabine Felser ______________________ 08.10.2020
(Study leader) Signature Date

Dr. med. Christina Große-Thie ______________________ 08.10.2020
(Deputy leader of study) Signature Date

II. Synopsis

**Study title**	Individual home exercise for patients with head and neck tumours -<br>A multicentre study to improve quality of life
**Study leader**	**Study leader: Dr. phil. Sabine Felser**<br>xxx<br>**Deputy leader of study**: **Dr. med. Christina Große-Thie**<br>xxx
**Participating institutions**	• Clinic III (Haematology, Oncology, Palliative Care), (Director: Prof. Dr. med. Christian Junghanß)<br>• Clinic and Polyclinic for Oral and Maxillofacial Plastic Surgery, Rostock University Medical Centre (Contact person: Dr. med. Jan Liese)<br>• Department of Otorhinolaryngology, Head and Neck Surgery "Otto Koerner", Rostock University Medical Center, Rostock, Germany. (Contact person: Dr. Daniel Strüder)<br>• Study centre of the Clinic III (Haematology, Oncology, Palliative Care), (Head: Dr. med. Brigitte Kragl)<br>• Institute of Biostatistics and Informatics in Medicine, Rostock University Medical Center, (Contact person: PD Dr. Änne Glass)<br>• Co-operation partners: OSHO e. V. clinics/practices
**Questions**	HNSCC patients often suffer from a high chronic symptom burden due to the location of the tumour and the intensive local treatment procedures, which impairs quality of life (QoL). There is strong evidence of the benefits of physical activity in terms of symptom relief and compensation for dysfunction. Physical activity can have a positive impact on body composition, muscular fitness, pain, flexibility and QoL. It is known from surveys that many HNSCC patients prefer to exercise at home, alone and at moderate intensity. In order to evaluate the feasibility and effects of individualised home training in HNSCC patients at different levels of QoL, the following questions will be answered in this study:<br><br>**Primary outcome:**<br>Phase A (Feasibility)<br>• Percentage of patients who complete the training intervention in a home-based setting over 12 weeks<br>Phase B (Quality of life)<br>• Change in global QoL score (EORTC-QLQ-30) after a 12-week training intervention in a home-based setting<br><br>**Secondary outcomes:**<br>• Reasons for premature discontinuation of the 12-week training intervention<br>• Frequency and duration of training in the home-based setting on average per week (training volume)<br>• Medium-term change in QoL after a 12-week training intervention in the home-based setting<br>• Short-term change in mobility after a 12-week training intervention in the home-based setting

	• Medium-term change in mobility after a 12-week training intervention in a home-based setting<br>• Short-term change in physical activity after a 12-week training intervention in a home-based setting<br>• Medium-term change in physical activity after a 12-week training intervention in a home-based setting<br>• Short-term change in balance ability/gait safety after a 12-week training intervention in a home-based setting<br>• Medium-term change in balance ability/gait safety after a 12-week training intervention in a home-based setting<br>• Short-term change in aerobic endurance capacity after a 12-week training intervention in a home-based setting<br>• Medium-term change in aerobic endurance capacity after a 12-week training intervention in a home-based setting<br>• Short-term changes in body composition after a 12-week training intervention in a home-based setting<br>• Medium-term changes in body composition after a 12-week training intervention in a home-based setting<br>• Correlations between the training volume during the 12-week training intervention and the effects with regard to QoL<br>• orrelations between the training volume during the 12-week training intervention and the effects on functionality<br>• Correlations between the training volume during the 12-week training intervention and the effects on body composition<br>• Relationships between daily activity and QoL<br>• Relationships between daily activity and functionality<br>• Correlations between daily activity and body composition<br>• Differences in the perception of the subjective effects of home training depending on gender
**Study duration**	21 months (12 months recruitment period)
**Study type, study design**	• single-arm prospective longitudinal study<br>• multicentre intervention study including questionnaire and functional diagnostics before and after completion of the intervention and 3 months follow-up
**Methodological approach**	• Local recruitment of study participants at the locations of the participating clinics/practices<br>• If the inclusion/exclusion criteria are met (physician), study information and consent is obtained (physician/therapist/documentarian)<br>• Initial diagnostics: General questionnaire to record personalised data, recording of QoL using the EORTC QLQ-30 and EORTC H&N35 questionnaires, recording of physical activity using the GSLTPAQ questionnaire<br>• Recording of disease-specific data (doctor)<br>• Performance diagnostics pre: recording of the current actual condition using functional diagnostics<br>• Data forwarding to the UMR study centre<br>• Centralised training planning and exercise composition, taking into account the patient's individual performance capacity

	• Transmission of individual training plans including exercise videos, exercise manual and small equipment to local therapists<br>• Local instruction of patients in the training programme and distribution of materials, exercise manual, individual training videos and training diary<br>• Implementation of the home training programme over 12 weeks, including documentation of the training (recording of the scope of training) and weekly call from the local therapist (questioning status quo, motivation, recording of adverse events)<br>• After completion of intervention: initial questionnaire to record motivation and questionnaire survey (EORTC QLQ-30, EORTC H&N35 and GSLT-PAQ) and performance diagnostics post analogue to diagnostics pre<br>• Follow-up examination after a further 12 weeks analogue pre/post<br>• Recording of the participants included in the study and the number of those who completed the 12-week training programme in full<br>• Recording/recording the reason for discontinuation
**Data basis**	Volume of training, patient reported outcomes and functional diagnostics
**Patients**	**Inclusion criteria:**<br>• Age ≥ 18 years<br>• Patients with tumour disease in the mouth, jaw, face and neck area in follow-up care, at least 4 months after antineoplastic therapy (radiotherapy, surgery, chemotherapy or immunotherapy) or after completion of follow-up treatment<br>• able to walk<br>• proficiency in written and spoken German<br>• Patient must be physically and psychosocially able to participate in the study (medical assessment)<br><br>**Exclusion criteria:**<br>Patients who fulfil at least one of the following criteria will not be included in this study:<br>• non-consenting patients<br>• not fulfilling all inclusion criteria<br>• clinically relevant heart failure (NYHA III and IV),<br>• myocardial infarction within the last 4 weeks<br>• unstable angina pectoris<br>• higher-grade valvular disease (in the medical history),<br>• uncontrolled cardiac arrhythmia,<br>• chronic obstructive pulmonary disease (GOLD III and IV)<br>• peripheral arterial occlusive disease (≥Stage III according to Fontaine)<br>• diseases that could seriously impair cognitive performance (e.g. dementia, stroke, Wernicke-Korsakoff syndrome)<br>• < 24 points on the Mini-Mental State Examination (MMSE)<br>• known alcohol dependency
**Sample size**	N = 60 (oh which 25 Phase A)
**Data analysis**	Personal data: Gender, age, height, weight, marital status, school-leaving qualification, professional status, attendance of self-help group

	<u>Nicotine and alcohol consumption</u><br><u>Disease-specific data:</u> Diagnosis incl. stage, year of diagnosis, medical treatment(s)<br><u>Data on sports history</u><br><u>Data on the intervention:</u> Adverse events, motivation, satisfaction with support and documentation, amount of training, possible reason for and time of drop-out<br>→ Estimation of the dropout rate<br>→ Descriptive statistics<br><br><u>Questionnaires (pre/post/follow up):</u> Information on QoL (EORTC QLQ-30, EORTC H&N35), physical activity (GSLTPAQ), assessment of current fatigue (POMS-F) on the day of the performance assessment<br>→ Calculation of the mean individual differences and testing for zero; t-test for dependent samples/ Wilcoxon/ McNemar<br>→ Analysis of variance with repeated measures<br><br><u>Functional diagnostics (pre/post/follow up)</u>:<br>Inter-tooth distance at maximum mouth opening, range of motion (ROM) of the shoulder joints and cervical spine, flexibility of the trunk (stand and reach test), Short Physical Performance Battery (balance, walking speed, leg strength), 6-minute walk test (cardiovascular and pulmonary performance) incl. BORG and CR-10 scale (perception of exertion and pain on exertion), optional bioimpedance analysis (BIA)<br>→ Calculation of the mean individual differences and testing for zero; t-test for dependent samples/ Wilcoxon/ McNemar<br>→ Distribution test for equality $\chi 2$ test; risk assessment or determination of association measures and testing for zero;<br>→ Analysis of variance with repeated measures<br>→ Correlation/regression analyses<br><br>The data is analysed in collaboration with IBIMA (Institute of Biostatistics and Informatics in Medicine)

III. Table of contents

I. Signatures 3
II. Synopsis 4
III. Table of contents 8
IV. List of abbreviations 10
1. Introduction 11
2. Aims oft he study 11
2.1. Rationale 11
2.2. Own preliminary work 11
2.3. Aim oft he study 12
2.4. Primary outcome 12
2.5. Secondary otcomes 12
3. Organisational structure 14
3.1. Sponsor 14
3.2. Participating institutions 14
3.3. Finanzierung 14
4. Studiendurchführung 15
4.1. Allgemeines Studiendesign 15
4.2. Discussion oft he study design 17
4.3. Selection oft he study population 17
4.3.1. Inclusion and exclusion criteria 17
4.4. Subsequent exclusion of study participants 18
4.5. Intervention 18
4.6. Dokumentation 19
4.6.2. Data management 20
4.6.3. Data analysis 20
4.6.4. Archiving 20
5. Ethical and regulatory aspects 21
5.1. Independent ethics committees 21
5.2. Ethical conduct of the clinical trial 21
5.2.1. Legal provisions and guidelines taken into account 21
5.3. Information and consent of the test subjects 21
5.4. Subject insurance 22
5.5. Data protection 22
5.5.1. Protection of personal data 22
5.5.2. Storage and disclosure of pseudonymised data 22

6. Statistical methods and determination of the sample size 23
6.1. Statistical and analytical plan 23
6.1.1. Study populations 23
6.1.2. Description of the patient collective 23
6.1.3. Primary outcome 23
6.1.4. Secondary outcomes 23
6.1.5. Subgroup analyses 24
6.2. Determination of the sample size 24
7. Safety 25
7.1. Definitions for adverse events 25
7.1.1. Adverse event (AE) 25
7.2. Documentation and tracking of AEs/SAEs 26
8. Use of the data and publication 27
8.1. Reports 27
8.1.1. Interims report 27
8.1.2. Final report 27
8.2. Publication 27
9. Changes of the study protocol 27
10. Literatur 28

IV. List of abbreviations

Abbrevations	Meaning
AE	Adverse Event
BIA	Bioimpedance analysis
BORG-Skala	Scale for recording the subjective perception of exertion
CIPN	Chemotherapy-induced peripheral neuropathy
EORTC QLQ-30	European Organization for Research and Treatment of Cancer Quality of Life Questionnaire
GSLTPAQ	Godin Leisure-Time Physical Activity Questionnaire
HNSCC	Head Neck Sarcoma Cell Cancer
IBIMA	Institute of Biostatistics and Informatics in Medicine
MMSE	Mini-Mental State Examination
OSHO	East German Haematology and Oncology Study Group e.V.
POMS-F	Profile of Moods States Fatigue
PRO	Patient Reported Outcome
QoL	Quality of life
ROM	Range of Motion
SAE	Serious Adverse Event
SPPB	Short Physical Performance Battery
TTB	Training diary
UMR	Rostock University Medical Centre
VAS	Visual analogue scale

1. Introduction

Worldwide, head and neck tumours (HNSCC) account for more than 685,000 cases with 375,000 deaths per year [1]. The Robert Koch Institute's forecast for 2020 shows around 20,000 new cases in Germany [2]. The therapeutic approaches are interdisciplinary due to the close collaboration between surgeons, radiotherapists, oncologists and other disciplines. Due to the location of the tumour and the intensive local therapy procedures, these patients often have a high chronic symptom burden. Many patients suffer from dysphonia, dysphagia, lymphoedema, weight loss, muscle atrophy, fatigue, chemotherapy-induced peripheral neuropathy (CIPN), pain and limited mobility, all of which affect quality of life (QoL) [3-6]. Therefore, interdisciplinary rehabilitation including treatment by physicians, psychologists, speech and movement therapists, nutritionists and occupational therapists is sought for HNSCC patients [7]. In general, rehabilitation aims to alleviate symptoms and compensate for dysfunction [3]. There is strong evidence for the benefits of exercise/physical activity in terms of body composition, muscular fitness, pain, flexibility and QoL for HNSCC patients [7-14]. Thus, exercise therapy is a key factor in patient well-being/outcome.

2. Aims oft he study

2.1. Rationale

Many previous intervention studies with HNSCC patients have focused on standardised training during aftercare. This often involved the use of large exercise machines, which are expensive or require specialised training facilities. Furthermore, most studies focussed on a single training method (e.g. progressive strength training) that only focused on one symptom [8,10-13]. However, as most patients suffer from multiple impairments (movement restriction, pain, muscle atrophy, CIPN, fatigue), there is a need for more comprehensive holistic training programmes. In addition, surveys show that many HNSCC patients prefer to exercise at home, alone and at moderate intensity [15,16]. Consequently, HNSCC patients need an exercise programme that can be carried out at home, without large/expensive exercise equipment and taking into account their physical and mental impairments. Home training programmes also offer the advantage that theoretically all HNSCC patients can benefit from them. The training programmes can be tailored to the patient's individual needs and can be carried out flexibly in terms of time and space. They are also not limited in time like conventional rehabilitation programmes.

2.2. Own preliminary work

An initial pilot study (A 2018-0153) was carried out at the UMR (ZIM III) in 2018/19 to develop and evaluate a home training programme for HNSCC without the use of large equipment. The first step was to put together a training programme with exercises that are suitable for independent training at home for HNSCC patients. This training programme was then evaluated by means of a 12-week training intervention with regard to patient compliance, the effects on physical functionality and QoL. In this study, the patients completed the training programme under supervision in small groups. The intervention comprised a total of 24 training sessions of 50 minutes each. On average, each session consisted of 15 minutes of warm-up/mobilisation (functional gymnastics), 10 minutes of coordination exercises, 15 minutes of strengthening exercises and 10 minutes of stretching/relaxation exercises. The training was carried out at low to medium intensity.

A total of 12 patients (6 male, 6 female, 68 ± 9 years) were included in the study. Ten (83%) completed the study successfully. The attendance rate of the participants was 83% (20 ± 3 of 24 units). The functional tests showed various significant improvements in the range of motion (ROM) of the shoulder joints and the cervical spine. At the end of the intervention, all participants scored full marks in the Short Physical Performance Battery (SPPB, test includes balance, leg strength and walking speed). In the 6-minute walk test, patients covered an additional distance of 43 metres (+8.3%) after the intervention, whereby the subjective perception of exertion was significantly lower. In order to evaluate changes in QoL, the EORTC-QLQ-30 and EORTC H&N35 questionnaires were completed by the participants before and after the intervention. The analysis showed a trend towards an improvement in global QoL ($p = 0.059$). The effect size corresponds to a medium effect ($d = 0.626$). Positive changes were seen in three of the five functional scales of the EORTC-QLQ-30: physical function (+6.7 points, $p = 0.008$, $d = 1.077$), cognitive function (+48.3 points, $p = 0.015$, $d = 0.829$) and social function (+23.4 points, $p = 0.031$, $d = 0.604$). There were no statistically significant changes in the three symptom scales and six single items. In the symptom scales of the H&N35, only sexuality showed a significant improvement (-16.5 points, $p = 0.031$, $d = 0.928$). The "social contact" scale showed a tendency towards improvement (-8.6 points, $p = 0.68$, $d = -0.542$) [17,18].

The good compliance of the participants, the positive effects in terms of physical functionality and QoL provide an excellent basis for the transfer to a home training programme (home-based setting).

As part of the pilot study, an "Exercise manual for patients with mouth, jaw, face and neck tumours" was written (ISBN: 978-1099096082). It contains a total of 90 mobilisation, coordination, strengthening and stretching exercises for training at home. Videos of the exercises have been created to accompany the book. Both the book and the videos will be made available to the HNSCC patients participating in the study for independent practice at home.

2.3. Aim oft he study

The aim of this study is to evaluate the approach of individual home exercise training in HNSCC patients in a multicentre study with regard to its feasibility and its effects on different degrees of QoL.

The following primary and secondary outcomes are defined in order to adequately fulfil all objectives of the study:

2.4. Primary outcome

Phase A (Feasibility)
Percentage of patients who complete the training intervention in a home-based setting over 12 weeks

Phase B (Quality of life)
Change in global QoL score (EORTC-QLQ-30) after a 12-week training intervention in a home-based setting

2.5. Secondary otcomes

- Reasons for premature discontinuation of the 12-week training intervention
- Frequency and duration of training in the home-based setting on average per week (training volume)

- Medium-term change in QoL after a 12-week training intervention in the home-based setting
- Short-term change in mobility after a 12-week training intervention in the home-based setting
- Medium-term change in mobility after a 12-week training intervention in a home-based setting
- Short-term change in physical activity after a 12-week training intervention in a home-based setting
- Medium-term change in physical activity after a 12-week training intervention in a home-based setting
- Short-term change in balance ability/gait safety after a 12-week training intervention in a home-based setting
- Medium-term change in balance ability/gait safety after a 12-week training intervention in a home-based setting
- Short-term change in aerobic endurance capacity after a 12-week training intervention in a home-based setting
- Medium-term change in aerobic endurance capacity after a 12-week training intervention in a home-based setting
- Short-term changes in body composition after a 12-week training intervention in a home-based setting
- Medium-term changes in body composition after a 12-week training intervention in a home-based setting
- Correlations between the training volume during the 12-week training intervention and the effects with regard to QoL
- orrelations between the training volume during the 12-week training intervention and the effects on functionality
- Correlations between the training volume during the 12-week training intervention and the effects on body composition
- Relationships between daily activity and QoL
- Relationships between daily activity and functionality
- Correlations between daily activity and body composition
- Differences in the perception of the subjective effects of home training depending on gender

3. Organisational structure

3.1. Sponsor

Prof. Dr. med. Christian Junghanß
xxx

3.2. Participating institutions

- Clinic III (Haematology, Oncology, Palliative Care), (Director: Prof. Dr. med. Christian Junghanß)
- Clinic and Polyclinic for Oral and Maxillofacial Plastic Surgery, Rostock University Medical Centre (Contact person: Dr. med. Jan Liese)
- Department of Otorhinolaryngology, Head and Neck Surgery "Otto Koerner", Rostock University Medical Center, Rostock, Germany. (Contact person: Dr. Daniel Strüder)
- Study centre of the Clinic III (Haematology, Oncology, Palliative Care), (Head: Dr. med. Brigitte Kragl)
- Institute of Biostatistics and Informatics in Medicine, Rostock University Medical Center, (Contact person: PD Dr. Änne Glass)
- Co-operation partners: OSHO e. V. clinics/practices
    - Helios Kliniken Schwerin
    - Universitätsmedizin Halle (Saale) / Krukenberg-Krebszentrum Halle
    - Ernst von Bergmann Klinikum Potsdam
    - Städtisches Klinikum Dresden
    - Universitätsklinikum Leipzig
    - Other OSHO e. V. centres, if applicable

3.3. Finanzierung

The study is financed by grants from OSHO e.V. (start-up funding), partly from third-party funds from the project "Exercise therapy during and after cancer therapy - a project to improve quality of life" funded by the Krebsgesellschaft M-V e. V. and from third-party funds from Clinic III (haematology, oncology, palliative medicine).

4. Studiendurchführung

4.1. Allgemeines Studiendesign

The study is a multi-centre intervention study including a questionnaire and functionality assessment before and after completion of the intervention as well as a three-month follow-up (prospective longitudinal study). The study procedure is shown schematically in Fig. 1.

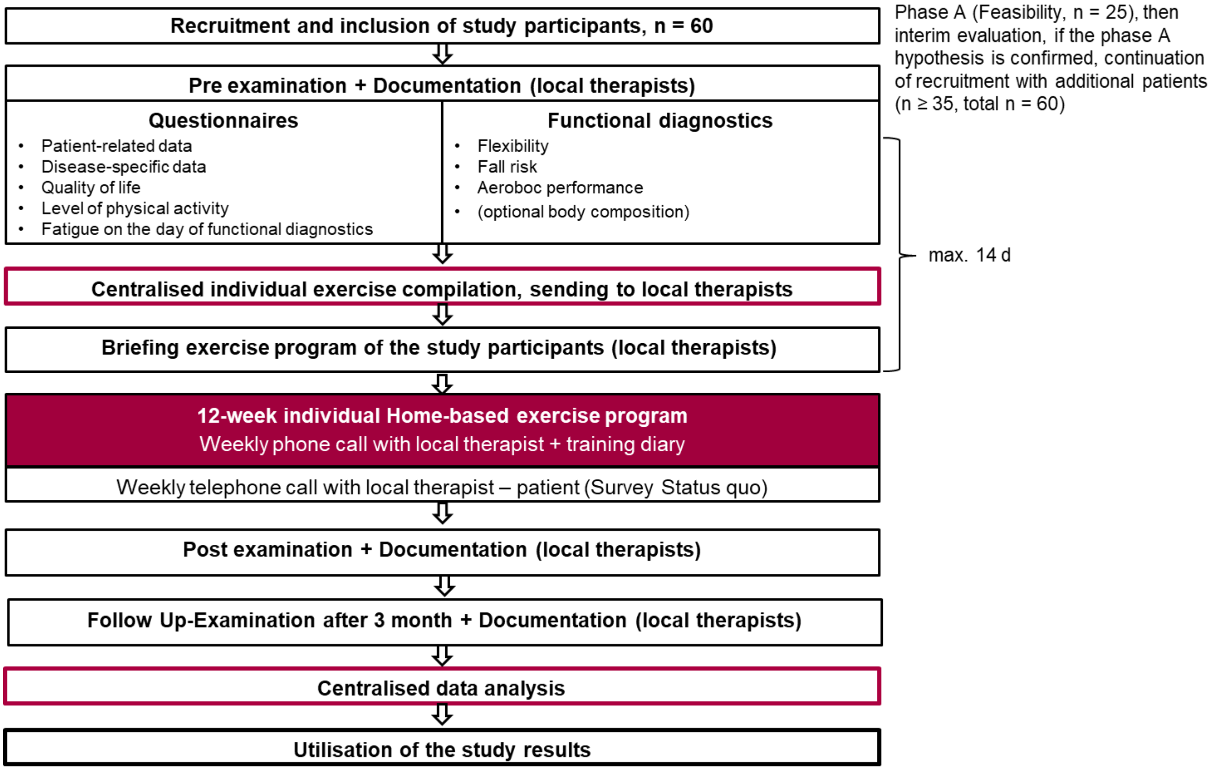

Fig. 1 Flowchart of the study process

Before recruiting the study participants at the individual sites, all therapists who are responsible for the initial and baseline diagnostics and for instructing the patient in the training programme are instructed in the questionnaires, measurements, protocols and exercise materials by a project employee at the UMR site. The therapists receive an overview of all tasks and documents that are to be handed out or completed at the respective examination times. During the entire study period, contact will be established between local therapists (doctors) and the UMR study management (by e-mail, telephone, video conference) if necessary (e.g. in case of questions).
Study participants are recruited locally at the locations of the participating centres/clinics/practices. If the inclusion/exclusion criteria are met, patient information is provided by a doctor/therapist or documentary. If the patient consents to the study, the initial diagnostics (pre-test) follows. This consists of two parts:

Part 1 „Patient Reported Outcomes" (PROs), and
Part 2 Functional diagnostics.

The PROs include:

a) Collection of disease-specific data regarding the tumour disease (treating/including physician)
b) Recording of person-specific data (e.g. side effects)
c) Recording of QoL using the EORTC QLQ-30 and EORTC H&N35 questionnaires
d) Recording of physical activity using the GSLTPAQ questionnaire.

The functional diagnostics to determine the current actual state includes

a) Measurement of the interdental distance at maximum mouth opening
b) Measurement of the ROM of the shoulder joints and the cervical spine
c) Measurement of the flexibility of the trunk using the "stand and reach test"
d) Performance of the "Short Physical Performance Battery" (balance, walking speed, leg strength),
e) Completion of the 6-minute walk test incl. BORG and CR-10 scale (perception of exertion and pain on exertion) and
f) optionally carrying out a bioimpedance analysis (BIA).

The collected data is then forwarded pseudonymised to the UMR study centre. This is where the individual training plan and exercise programme are compiled centrally, taking into account the patient's current performance/restrictions. Once the training plan, including exercise compilation (video), has been sent to the local therapist, the patient is instructed locally in the training programme and materials. The patient is given instructions on home training (frequency, duration, intensity, information on when training should not be carried out), an exercise manual, a personalised training video and the necessary materials (rubber band, exercise ball). In addition, the therapist explains how to complete the training diary (TTB).
The aim is for there to be a maximum of 14 days between the initial diagnosis and training instruction.
After instruction, the training is carried out in a home-based setting over a period of 12 weeks. The patient documents the training duration daily in the TTB. The local therapist calls once a week to enquire about the status quo (well-being, training frequency and duration, occurrence of adverse events during training (AEs/SAEs), motivation). The therapist records the information in a telephone log. At the same time, the call is intended to serve as motivation. If patients discontinue the intervention prematurely, the reason for discontinuation is asked.
The 12-week training intervention is followed by the initial diagnosis (post-test). This is carried out in the same way as the initial diagnosis. Only the general questionnaire is replaced by an exit questionnaire. This questionnaire measures satisfaction with the initiation and the materials as well as motivation to continue training. On the day of the initial diagnosis, the patient hands over their TTB to the therapist. All data, including the TTB, is then forwarded to the UMR study centre in pseudonymised form.
Three months after the initial diagnosis, the patient receives an appointment for the follow-up examination. The questionnaires are sent to the patient approximately one week before the appointment. PROs and performance diagnostics are carried out in the same way as the pre- and post-tests. After the examination, all data is forwarded pseudonymised to the UMR study centre, where the central data collection and evaluation takes place.

Patient-related:

1. information and consent
2. pre examination
3. intervention: 12 weeks in a home-based setting after admission
4. post examination
5. follow-up examination after 3 months

Study-related:

Recruitment period: 12 months
Total duration of intervention incl. follow-up (data collection): 18 months
Total duration incl. final report: 21 months

Inclusion of first patient	01.01.2021
Inclusion of last patient	31.12.2021
End of examination of the last patient	30.06.2022
Integrated final report	30.09.2022

Table 1 Schedule of the study

4.2. Discussion oft he study design

Primarily, the short and medium-term effect of home training on the QoL of the patients is analysed. This means that the focus is on the intra-individual change in QoL between the time points pre (before the intervention), post (after the intervention) and follow-up. A control group is therefore not absolutely necessary.

4.3. Selection oft he study population

Only patients with a diagnosis coded with a final digit according to ICD: C00 - C14, C30 - C32 Malignant neoplasms of the lip, oral cavity, pharynx, upper respiratory tract ("head and neck tumours") are included. No persons in need of special protection are included.

In order to be able to assess the extent of training and the effects of home training in relation to gender, men and women should be included in the study in equal proportions if possible. However, it must be taken into account that three times as many men as women suffer from head and neck tumours [2], participation in the study is voluntary and depends, among other things, on the motivation of the patients as well as their state of health (inclusion and exclusion criteria).

4.3.1. Inclusion and exclusion criteria

The recruitment of study participants is subject to certain inclusion and exclusion criteria, which are listed below:

Inclusion criteria:

- Age ≥ 18 years
- Patients with tumour disease in the mouth, jaw, face and neck area in follow-up care, at least 4 months after antineoplastic therapy (radiotherapy, surgery, chemotherapy or immu-notherapy) or after completion of follow-up treatment

- able to walk
- proficiency in written and spoken German
- Patient must be physically and psychosocially able to participate in the study (medical assessment)

Exclusion criteria:
Patients who fulfil at least one of the following criteria will not be included in this study:

- non-consenting patients
- not fulfilling all inclusion criteria
- clinically relevant heart failure (NYHA III and IV),
- myocardial infarction within the last 4 weeks
- unstable angina pectoris
- higher-grade valvular disease (in the medical history),
- uncontrolled cardiac arrhythmia,
- chronic obstructive pulmonary disease (GOLD III and IV)
- peripheral arterial occlusive disease (≥Stage III according to Fontaine)
- diseases that could seriously impair cognitive performance (e.g. dementia, stroke, Wernicke-Korsakoff syndrome)
- < 24 points on the Mini-Mental State Examination (MMSE)
- known alcohol dependency

4.4. Subsequent exclusion of study participants

Subsequent exclusion from the study takes place if a patient withdraws their consent.

4.5. Intervention

The training intervention takes place in a home-based setting over a period of 12 weeks. Patients are recommended to do the individually tailored exercises at least three days a week. The training duration per day should be 15 - 30 minutes. The patient is also recommended (in addition to training the exercises) to do endurance training 2 to 3 times a week for around 30 minutes, e.g. in the form of a walk, (Nordic) walking, cycling, swimming, dancing or similar. In order to avoid overloading through training, the BORG scale (see Appendix 6) is recommended for controlling the training. The Borg scale from 6 ("very, very easy") to 20 ("very, very strenuous") measures the individually perceived level of exertion during strength and endurance training. The optimum effort lies between the values 11 and 15.

The individual exercise programme is put together by sports scientists and/or physiotherapists based on the results of the initial diagnostics. Various mobilisation, coordination, strengthening and stretching exercises are used according to the patient's performance capacity/restrictions, which are believed to have positive effects on physical performance, functionality and QoL. All recommended exercises can be found in the "Exercise manual for patients with mouth, jaw, face and neck tumours", which the study participants receive. In addition, the selected exercises are saved as a video on a data carrier (DVD/stick), which the patient also receives. Depending on the feedback from the participants, it is possible to recommend further exercises after the first training sessions and make them available as video files (e.g. more demanding or more difficult exercises to improve performance). Materials such as elastic bands and exercise balls, which are required for the exercises, are provided to the patient free of charge.

The patient records their training in a training diary. Once a week, a local therapist calls the patient to enquire about the status quo and records this.

4.6. Dokumentation

The following questionnaires are used for the pre, post and follow-up examinations:

- Questionnaire for recording disease-specific data (Appendix 1)
- General questionnaire for the collection of personal data (only initial examination) (Appendix 2)
- EORTC-QLQ-30 and EORTC Module H&N 35: Recording of QoL (Appendix 3)
- Godin Leisure-Time Physical Activity Questionnaire (GSLTPAQ): Assessment of physical activity (Appendix 4)
- Final questionnaire: assessment of satisfaction with training programme and media/small equipment provided and supervision, current motivation of study participants with regard to continuation of the training programme (final examination only) (Appendix 5)

Various tests are used to objectively assess physical performance, functionality and body composition. These include:

- Profile of moods states fatigue scale (POMS-F): recording the current mood
- Measurement of the incisal edge difference (SKD): recording the mobility of the temporomandibular joint
- Range of motion (ROM) measurement of the cervical spine and shoulder joints using manual goniometers (objective measurements) and assessment of the extent of movement/restrictions to movement from the patient's and therapist's perspective (subjective information)
- Stand and reach test: assessment of the mobility of the back muscles
- SPPB: Test battery to assess balance ability, leg strength and walking speed
- 6-minute walking test incl. BORG and CR-10 scale: Assessment of aerobic endurance capacity, recording of the subjective feeling of exertion and subjective exertion pain
- Optional BIA: measurement of body composition

The total duration of the performance diagnostics is approximately 30 minutes. The results are documented in a protocol (Appendix 6).

The examinations listed are carried out both before (pre-test) and after the home-based intervention (post-test) as well as three months follow-up.

For all patients, the extent of training is documented in a TTB (Appendix 7). In the event of a drop-out, the reason for the cancellation is recorded.

The weekly telephone calls on the status quo (scope of training, motivation) are recorded in writing (Appendix 8).

4.6.1. Data collection

Both personal and disease-specific data are collected as part of this study. Information on QoL is collected by means of questionnaires. Performance diagnostics data and information on the intervention are also collected. The data collected in detail is summarised below:

Personal data: Gender, age, height, weight, marital status, school-leaving qualification, professional status, attendance of self-help group
Nicotine and alcohol consumption
Disease-specific data: Diagnosis incl. stage, year of diagnosis, medical treatment(s)
Data on sports history
Data on the intervention: Adverse events, motivation, satisfaction with support and documentation, amount of training, possible reason for and time of dropout

Questionnaires (pre/post/follow up): Information on QoL (EORTC QLQ-30, EORTC H&N35), physical activity (GSLTPAQ), assessment of current fatigue (POMS-F) on the day of the performance assessment

Functional diagnostics (pre/post/follow up): Inter-tooth distance at maximum mouth opening, range of motion (ROM) of the shoulder joints and cervical spine, flexibility of the trunk (stand and reach test), Short Physical Performance Battery (balance, walking speed, leg strength), 6-minute walk test (cardiovascular and pulmonary performance) incl. BORG and CR-10 scale (perception of exertion and pain on exertion), optional bioimped-ance analysis (BIA)

4.6.2. Data management

The data is collected in the participating centres/clinics/practices and sent pseudonymised online via an encrypted connection to the UMR study centre. No personal data (e.g. name, date of birth, address, telephone number, etc.) will be sent. As a result, it is not possible to assign the data to a person, even if the wrong recipient is sent by mistake.

4.6.3. Data analysis

This study is a prospective longitudinal study. The data will be analysed by estimating the drop-out rate, descriptive statistics, correlation tests (correlation and regression analysis) and multi-factorial methods for testing differences (e.g. analysis of variance with repeated measures). The data will be analysed in cooperation with the IBIMA of the UMR.
The evaluation with regard to phase I (feasibility) will take place after 25 patients have been included in the study. In addition, ad hoc analyses will be performed. In the case of rapid recruitment, this will be paused at 60 patients included in the study and, if necessary, continued depending on the results of the analyses.

4.6.4. Archiving

The patient identification lists as well as all documentation forms, consent forms and other important trial documents are stored at the respective trial centres for at least 15 years in accordance with Section 13 (10) GCP-V.

5. Ethical and regulatory aspects

5.1. Independent ethics committees

A favourable assessment of the study is obtained from the responsible ethics committee. Only then is the study started. In each additional trial centre, the study is only conducted after the responsible ethics committee involved has determined the suitability of the trial site and the qualifications of the investigators.

5.2. Ethical conduct of the clinical trial

This protocol and any subsequent amendments to the protocol have been or will be drawn up in accordance with the Declaration of Helsinki as amended in October 1996 (48th General Assembly of the World Medical Association, Somerset West, Republic of South Africa).

5.2.1. Legal provisions and guidelines taken into account

The present study will be conducted in accordance with the published principles of the Good Clinical Practice (ICH-GCP) guideline and the applicable legal requirements (in particular the GCP regulation). These principles include ethics committee procedures, patient information and informed consent, protocol compliance, administrative documents, data collection and record keeping. To protect personal data, this study will be conducted in accordance with the DSGVO.

5.3. Information and consent of the test subjects

A patient can only be included in the study if they have given their consent to this after being informed verbally and in writing by a doctor, therapist or documentary about the nature, significance and scope of the study in an appropriate and comprehensible manner. At the same time as giving their consent, they must have declared that they agree to the data being recorded as part of the study and to it being reviewed by the responsible supervisory or federal authority. It must be clear to them that they can withdraw their consent at any time without giving reasons and without suffering any disadvantages as a result.

The original written consent will be kept in the study folder of the trial centre. The patient will be given a copy of the written patient information and the declaration of consent.

Patient information and consent form are attached.

The patient information and consent form, all other documents received by participants and any recruitment advertisements are submitted to the responsible ethics committee for approval before use.

Public media, notices and flyers in ENT and maxillofacial clinics/practices, oncology centres and publications on the homepage of the participating clinics/practices will be used to draw attention to the study and publicise it (Appendix 10). The study will also be publicised via posters and flyers, which will also be sent to self-help groups. Patients in follow-up care who qualify for the study will be approached directly about the study by the attending physician. All patients who are potentially eligible as study participants and would like to take part in the study will be informed and educated about the study. Participation in the study is voluntary. The confidentiality of the data is always observed and guaranteed.

5.4. Subject insurance

This study is not a study subject to compulsory insurance within the meaning of the AMG, the MPG or the Radiation Protection or X-ray Ordinance. No special (commuting) accident insurance will be taken out.

5.5. Data protection

5.5.1. Protection of personal data

The principal investigator protects the right of patients to their personal data and ensures that it is handled responsibly. All persons who have patient contact within the scope of the study or who have access to disease data or other data are obliged to maintain confidentiality. Data is only passed on in pseudonymised form and published in aggregated form. This means that tracing back to individual persons is largely excluded. All study participants must ensure that documents with names (e.g. the signed consent forms and the patient identification list) are treated confidentially and kept protected from unauthorised access. Only the pseudonymised patient identification is stated on documentation forms and any other documents that are forwarded to study participants.

5.5.2. Storage and disclosure of pseudonymised data

As part of the information provided for the study, patients are informed that the data collected will be forwarded to the study centre of Clinic III, Haematology, Oncology and Palliative Medicine at the UMR. Patients are informed that, as part of the study, all data will be forwarded to the UMR study centre exclusively in pseudonymised form, stored there and used for scientific evaluations and publications. No personal data will be sent. Persons who do not consent to their data being passed on cannot take part in the study.

6. Statistical methods and determination of the sample size

6.1. Statistical and analytical plan

The drop-out rate is estimated to determine the primary outcome of phase A (feasibility).

The statistics of the primary and secondary outcomes (phase B) include: descriptive statistics, calculation of the mean individual differences and testing for zero; t-test for dependent samples/Wilcoxon/McNemar), distribution test for equality $\chi 2$-test; risk assessment or determination of association measures and testing for zero; correlation/regression and analysis of variance with repeated measures.

6.1.1. Study populations

Only patients with a diagnosis coded with a final digit according to ICD: C00 - C14, C30 - C32 Malignant neoplasms of the lip, oral cavity, pharynx, upper respiratory tract ("head and neck tumours") are included. No persons requiring special protection are included.

6.1.2. Description of the patient collective

If possible, men and women should be included in the study in the same proportion. Antineoplastic therapy (radiotherapy, surgery, chemotherapy or immunotherapy) must have been completed for at least 4 months (or after completion of follow-up treatment).

6.1.3. Primary outcome

This study consists of two phases.

Phase A (feasibility & quality of life, n = 25):

The hypothesis is tested that the proportion of patients completing the training intervention in the home-based setting over 12 weeks is ≥ 70%.

Phase B (quality of life, n = 35, total n = 60):

The hypothesis will be tested that the global QoL score (EORTC-QLQ-30) will increase significantly after a 12-week training intervention in the home-based setting.

6.1.4. Secondary outcomes

Due to the complexity of the study, there are several secondary outcomes, which are listed below:

- Reasons for premature discontinuation of the 12-week training intervention
- Frequency and duration of training in the home-based setting on average per week (training volume)
- Medium-term change in QoL after a 12-week training intervention in the home-based setting
- Short-term change in mobility after a 12-week training intervention in the home-based setting

- Medium-term change in mobility after a 12-week training intervention in a home-based setting
- Short-term change in physical activity after a 12-week training intervention in a home-based setting
- Medium-term change in physical activity after a 12-week training intervention in a home-based setting
- Short-term change in balance ability/gait safety after a 12-week training intervention in a home-based setting
- Medium-term change in balance ability/gait safety after a 12-week training intervention in a home-based setting
- Short-term change in aerobic endurance capacity after a 12-week training intervention in a home-based setting
- Medium-term change in aerobic endurance capacity after a 12-week training intervention in a home-based setting
- Short-term changes in body composition after a 12-week training intervention in a home-based setting
- Medium-term changes in body composition after a 12-week training intervention in a home-based setting
- Correlations between the training volume during the 12-week training intervention and the effects with regard to QoL
- orrelations between the training volume during the 12-week training intervention and the effects on functionality
- Correlations between the training volume during the 12-week training intervention and the effects on body composition
- Relationships between daily activity and QoL
- Relationships between daily activity and functionality
- Correlations between daily activity and body composition
- Differences in the perception of the subjective effects of home training depending on gender

6.1.5. Subgroup analyses

Effects of the home-based intervention on QoL, functionality and physical activity will be analysed by means of subgroup analyses depending on gender and training volume.

6.2. Determination of the sample size

The case number calculation of phase I is based on the calculation that with n = 24, a 30% dropout rate can be estimated with an accuracy of ± 18% at a confidence level of 1-α = .95 (2-sided) (nQuery® Advisor 7.0 Statistical Solutions Ltd., Boston, MA, USA).

The sample size calculation for the trial (phase B) is based on the results of Felser et al. (28): global QoL pre: 50.1 ± 16.4; post: 58.3 ± 16.2; r = 0.618 resulting in an effect size of 0.5755, which is detectable on a confidence level of 1 - α = .95 (2-sided) with n = 42 patients (nQuery® Advisor 7.0 Statistical Solutions Ltd., Boston, MA, USA). Assuming a 30% dropout rate based on our experience, a total of N = 60 patients is required to be included in the study.

7. Safety

To date, there is little data available on the occurrence of adverse events (AEs) in the routine care of oncological patients. To date, the occurrence of AEs in the training of oncological patients has mainly been recorded in the context of clinical studies. Based on previous results, training with oncological patients is considered to be feasible and safe [19,20], as the probability of an "adverse event" occurring can be considered low [21].

No medication is administered as part of this study. All examinations as part of the performance diagnostics are non-invasive. The training takes place in a home-based setting. The recommended exercises are adapted to the individual patient's state of health and current performance capacity. The training times, training volume and training intensity are determined independently by the study participants. It is expressly pointed out that training should not be carried out in the event of nausea/vomiting, severe pain, impaired consciousness and confusion, dizziness, circulatory problems, fever > 38° or severe infection. Nevertheless, incidents (e.g. nausea/vomiting, muscular complaints, circulatory problems) cannot be ruled out due to the unaccustomed strain.

In order to record any incidents that may occur, the patients are called once a week by the local therapist during the home-based intervention and the status quo is enquired about and incidents that were supposedly related to the home-based training are documented. A distinction is made between AEs and SAEs.

7.1. Definitions for adverse events

There is currently no clear definition of adverse events during training with oncological patients. The term "adverse events", originally established in medicine, is used to record and analyse adverse events during drug trials. In the field of oncological exercise therapy, the definition of adverse events is different than in drug trials. To classify different degrees of severity, serious adverse events (SAEs) and non-serious adverse events (AEs) are defined as follows:

7.1.1. Adverse event (AE)

„These are health incidents that lead to a short-term impairment of physical functioning or health and are at most clarified by a doctor, but do not require treatment" [21].

The AE does not necessarily have to be causally related to the training. All events that occur during training (including performance diagnostics) are recorded as AEs.

AEs include, for example, dizziness, nausea or vomiting occurring during training or performance diagnostics. They also include sprains, ligament strains, torn muscle fibres and the like caused by training.

7.1.2. Serious adverse event (SAE)

"These are health incidents during training that result in a medium or long-term impairment of physical functioning or health and result in medical treatment (death, cardiac arrest requiring resuscitation, broken bones, incisional fractures and injuries)." [21]

SAEs during the performance of the study are incidents that are

a) are fatal or life-threatening,

b) require unscheduled hospitalisation and/or

c) require medium-term health impairment with medical treatment (e.g. conventionally treated bone fracture)

The SAE does not necessarily have to be causally related to the training. Events are considered life-threatening in the above context if there was a risk of death at the time of the event.

Any inpatient stay of a study participant that lasted at least one night (10 pm - 6 am) is considered a hospitalisation.

7.2. Documentation and tracking of AEs/SAEs

During the 12-week training intervention, which takes place in a home-based setting, all study participants are called once a week by their local therapist. The therapist asks about any adverse events (AEs and SAEs) that occurred during the training programme and documents these in a "telephone log" (Appendix 8). It should also be recorded during which the event occurred (endurance training, strength training, balance training, etc.).

After completion of the training intervention, the protocols are sent to the study management/study centre of the UMR, where all documented AEs/SAEs are collated.

During the follow-up period (12-week follow-up), the study participants inform their therapist of any incidents that occurred during training. In such cases, the therapist notifies the study centre of Clinic III, Haematology, Oncology and Palliative Medicine at UMR by fax or e-mail after being informed.

8. Use of the data and publication

8.1. Reports

8.1.1. Interims report

An interim report will be prepared for 25 patients included in the study and submitted to the project leader and all investigators of the participating trial centres.

8.1.2. Final report

A final report will be written up to three months after the end of the study and submitted to the project leader and all investigators of the participating trial centres.

8.2. Publication

It is planned to present the results of the study in due course in scientific journals and/or at German and international congresses. For all publications, data protection will be ensured for all data of the persons concerned as well as for the data of the participating investigators.

Registration of the study in a public register in accordance with the recommendations of the International Committee of Medical Journal Editors (ICMJE) is also planned.

Publication or presentation of the results of this study, including publication or presentation by a single trial centre, is subject to prior acknowledgement, comment and approval by the sponsor. The number and order of authors for publications is/will be regulated in the cooperation agreement.

9. Changes of the study protocol

For the purpose of ensuring largely comparable conditions in the interest of proper data evaluation, no changes to the agreed test conditions laid down in the test plan are envisaged. In exceptional cases, however, changes to the trial conditions are possible. These will only be made after mutual agreement between the sponsor, the sponsor representative and all signatories (authors) of this protocol. Any change to the study procedures provided for in the protocol must be made in writing, stating the reasons for the change, and must be signed by all authors of this protocol (amendment).

Subsequent amendments requiring approval according to § 10 para. 1 and 4 GCP-V will be submitted to the Ethics Committee for approval and will only be implemented after their approval. This does not apply to amendments that are necessary to avert immediate danger.

10. Literatur

[1] B. W. Stewart and C. P. Wild, "World Cancer Report 2014," EBL-Schweitzer, International Agency for Research on Cancer/World Health Organization, Lyon, 2014.

[2] Robert Koch-Institut (RKI), "Bericht zum Krebsgeschehen in Deutschland 2016,"

[3] E. J. Borghardt, "Rehabilitation bei Patienten mit Kopf-Halstumoren," In: H.-J. Schmoll, Ed., *Indikationen, Therapiekonzepte und spezielle Therapiemodalitäten*. 4, Springer Medizin Verl., Heidelberg, 2006, pp. 1363–1372.

[4] M. Couch, V. Lai, T. Cannon, D. Guttridge, A. Zanation, J. George et al., "Cancer cachexia syndrome in head and neck cancer patients: part I. Diagnosis, impact on quality of life and survival, and treatment," *Head & neck*, Vol. 29, No. 4, 2007, pp. 401–411. doi:10.1002/hed.20447.

[5] J. B. Epstein, M. Robertson, S. Emerton, N. Phillips and P. Stevenson-Moore, "Quality of life and oral function in patients treated with radiation therapy for head and neck cancer," *Head & neck*, Vol. 23, No. 5, 2001, pp. 389–398.

[6] J. A. Langendijk, P. Doornaert, I. M. Verdonck-de Leeuw, C. R. Leemans, N. K. Aaronson and B. J. Slotman, "Impact of late treatment-related toxicity on quality of life among patients with head and neck cancer treated with radiotherapy," *Journal of clinical oncology*, Vol. 26, No. 22, 2008, pp. 3770–3776. doi:10.1200/JCO.2007.14.6647.

[7] M. Eades, J. Murphy, S. Carney, S. Amdouni, J. Lemoignan, M. Jelowicki et al., "Effect of an interdisciplinary rehabilitation program on quality of life in patients with head and neck cancer: review of clinical experience," *Head & neck*, Vol. 35, No. 3, 2013, pp. 343–349. doi:10.1002/hed.22972.

[8] L. C. Capozzi, K. R. Boldt, H. Lau, L. Shirt, B. Bultz and S. N. Culos-Reed, "A clinic-supported group exercise program for head and neck cancer survivors: managing cancer and treatment side effects to improve quality of life," *Sports medicine*, Vol. 23, No. 4, 2015, pp. 1001–1007. doi:10.1007/s00520-014-2436-4.

[9] L. C. Capozzi, K. C. Nishimura, M. L. McNeely, H. Lau and S. N. Culos-Reed, "The impact of physical activity on health-related fitness and quality of life for patients with head and neck cancer: a systematic review," *British journal of sports medicine*, Vol. 50, No. 6, 2016, pp. 325–338. doi:10.1136/bjsports-2015-094684.

[10] S. Lønbro, U. Dalgas, H. Primdahl, J. Overgaard and K. Overgaard, "Feasibility and efficacy of progressive resistance training and dietary supplements in radiotherapy treated head and neck cancer patients-the DAHANCA 25A study," *Acta oncologica*, Vol. 52, No. 2, 2013, pp. 310–318. doi:10.3109/0284186X.2012.741325.

[11] M. L. McNeely, M. Parliament, K. S. Courneya, H. Seikaly, N. Jha, R. Scrimger et al., "A pilot study of a randomized controlled trial to evaluate the effects of progressive resistance exercise training on shoulder dysfunction caused by spinal accessory neurapraxia/neurectomy in head and neck cancer survivors," *Head & neck*, Vol. 26, No. 6, 2004, pp. 518–530. doi:10.1002/hed.20010.

[12] M. L. McNeely, M. B. Parliament, H. Seikaly, N. Jha, D. J. Magee, M. J. Haykowsky et al., "Effect of exercise on upper extremity pain and dysfunction in head and neck cancer survivors: a randomized controlled trial," *Cancer*, Vol. 113, No. 1, 2008, pp. 214–222. doi:10.1002/cncr.23536.

[13] N. Pauli, B. Fagerberg-Mohlin, P. Andréll and C. Finizia, "Exercise intervention for the treatment of trismus in head and neck cancer," *Acta oncologica*, Vol. 53, No. 4, 2014, pp. 502–509. doi:10.3109/0284186X.2013.837583.

[14] L. Sammut, M. Ward and N. Patel, “Physical activity and quality of life in head and neck cancer survivors: a literature review,” *International journal of sports medicine*, Vol. 35, No. 9, 2014, pp. 794–799. doi:10.1055/s-0033-1363984.

[15] A. W. Midgley, D. Lowe, A. R. Levy, V. Mepani and S. N. Rogers, “Exercise program design considerations for head and neck cancer survivors,” *European archives of oto-rhino-laryngology*, Vol. 275, No. 1, 2018, pp. 169–179. doi:10.1007/s00405-017-4760-z.

[16] L. Q. Rogers, J. Malone, K. Rao, K. S. Courneya, A. Fogleman, A. Tippey et al., “Exercise preferences among patients with head and neck cancer: prevalence and associations with quality of life, symptom severity, depression, and rural residence,” *Head & neck*, Vol. 31, No. 8, 2009, pp. 994–1005. doi:10.1002/hed.21053.

[17] S. Felser, M. Behrens, Strüder D, Liese J, Rohde K, Junghanss C et al., “Feasibility and Effects of a Supervised Exercise Program Suitable for Independent Training at Home on Physical Function and Quality of Life in Head and Neck Cancer Patients: A Pilot Study,” *Integrative cancer therapies*, Vol. 19, 2019, 1-12. doi:10.1177/15347354209189.

[18] S. Felser, M. Behrens, K. Rhode, Liese J, D. Strüder, C. Junghanss et al., “Effects of a 12-week low to moderate intensity exercise intervention on physical function and quality of life in head and neck cancer survivors.,” *P388, Abstract-USB-Stick Jahrestagung der Deutschen, Österreichischen und Schweizerischen Gesellschaften für Hämatologie und Medizinische Onkologie*, 2019, ISSN 1863-1819.

[19] R. Heywood, A. L. McCarthy and T. L. Skinner, “Safety and feasibility of exercise interventions in patients with advanced cancer: a systematic review,” *Supportive care in cancer official journal of the Multinational Association of Supportive Care in Cancer*, Vol. 25, No. 10, 2017, pp. 3031–3050. doi:10.1007/s00520-017-3827-0.

[20] B. Singh, R. R. Spence, M. L. Steele, C. X. Sandler, J. M. Peake and S. C. Hayes, “A Systematic Review and Meta-Analysis of the Safety, Feasibility, and Effect of Exercise in Women With Stage II+ Breast Cancer,” *Archives of physical medicine and rehabilitation*, Vol. 99, No. 12, 2018, pp. 2621–2636. doi:10.1016/j.apmr.2018.03.026.

[21] D. Clauss, F. Quirmbach, J. Wiskemann and F. Rosenberger, “Adverse Events beim Training mit onkologischen Patienten: Wie sicher ist das Training außerhalb klinischer Studien?,” Vol. 2019, No. 04, 35, pp. 194–201. doi:10.1055/a-9057-1883.
